# Head circumference and intelligence, schooling, employment, and income: a systematic review

**DOI:** 10.1186/s12887-024-05159-2

**Published:** 2024-11-07

**Authors:** Marina B O Freire, Rebeccah Slater, Thiago M Santos, Bruna G C da Silva, Luke Baxter, Ana M B Menezes

**Affiliations:** 1 Postgraduate Program in Epidemiology, Federal University of Pelotas, R Mal. Deodoro, 1160 - Centro, Pelotas, RS 96020-220 Brazil; 2 Department of Paediatrics, University of Oxford, Oxford, UK; 3 Gender and Women’s Health Unit, Nossal Institute for Global Health, School of Population and Global Health, University of Melbourne, Unit 2/32 Lincoln Square N, Carlton VIC 3053, Melbourne, Australia

**Keywords:** Head circumference, Intelligence, Cognition, Schooling, Academic performance, Educational attainment, Employment, Income, Systematic review

## Abstract

**Background:**

No consensus exists about the role of head circumference in identifying children at risk of suboptimal development. The objective of this study was to evaluate the association between head circumference and intelligence, schooling, employment, and income. The review 1) summarizes the overall evidence and 2) restricts the evidence to a subset of articles that met minimum quality criteria.

**Methods:**

PubMed, Web of Science, PsycINFO, LILACS, CINAHL, WHO Institutional Repository for Information Sharing and UNICEF Innocenti were searched to identify published studies. Cohort, case–control or cross-sectional studies which evaluated the associations of interest in the general population, premature babies, babies with low birth weight or small for gestational age were included; head circumference must have been measured before the age of 20 years. Two reviewers independently performed study selection, data extraction and quality assessments.

**Results:**

Of 2521 records identified, 115 were included and 21 met the minimum quality criteria. Ninety studies investigated if early measures of head circumference predict later outcomes and 25 studies measured head circumference and the outcome at the same timepoint; 78 studies adjusted the head circumference for age and sex. We identified large heterogeneity and inconsistency in the effect measures and data reported across studies. Despite the relatively large number of included articles, more than 80% presented serious limitations such as lack of adjustment for confounding and severe selection bias. Considering the subset of articles which met the minimum quality criteria, 12 of 16 articles showed positive association between head circumference and intelligence in the general population. However, in premature babies, 2 of 3 articles showed no clear effect. Head circumference was positively associated with academic performance in all investigated samples (5 of 5 articles). No article which evaluated educational attainment and employment met the minimum quality criteria, but the association between head circumference and these outcomes seems to be positive.

**Conclusions:**

Larger head circumferences are positively associated with higher levels of intelligence and academic performance in the general population, but there is evidence of non-linearity in those associations. Identifying a group of children in higher risk for worse outcomes by a simple and inexpensive tool could provide an opportunity to mitigate these negative effects. Further research is needed for a deeper understanding of the whole distribution of head circumference and its effect in premature babies. Authors should consider the non-linearity of the association in the data analysis.

**Trial registration:**

Association between head circumference and intelligence, educational attainment, employment, and income: A systematic review, CRD42021289998.

**Supplementary Information:**

The online version contains supplementary material available at 10.1186/s12887-024-05159-2.

## Background

The role of intelligence throughout the life course is well established, with higher intelligence quotient (IQ) being associated with academic and employment success, higher income in later life and lower morbidity and mortality rates [1]. Additionally, low socio-economic level in childhood has been identified as the most important predictor of low IQ [2]. Considering this, many children in low-and middle-income countries are at risk of not reaching their developmental potential, which is particularly important in children who experience early life adversities such as poverty and health and nutrition problems [3]. Despite progress in reducing poverty worldwide in the past decades, the prevalence of maternal and child undernutrition in low-and middle-income countries has remained unacceptably high.

In this context, faltering growth occurs largely in the first thousand days of life, during a critical growth period extending from conception to the second birthday [4]. Damage during this time may also compromise brain development, as brain size reaches 55% of its adult volume at two years of age and 90% at age six [5]. While weight and length reflect body size, head circumference appears to be independently related to brain size and acquisition of intelligence [6].

Different factors could affect intrauterine brain growth. Fetal growth restriction (FGR), broadly defined as an estimated fetal weight or abdominal circumference lower than 10th percentile for gestational age, compromises head circumference for gestational age in 20 to 30% of the cases [7, 8]. Previous studies found that infectious diseases during pregnancy were responsible for 10 to 15% of FGR, particularly rubella, cytomegalovirus, varicella-zoster and toxoplasmosis [7]. Other well-known risk factors are smoking and alcohol use during pregnancy, multiple gestation, hypertensive disorders, severe anemia and diabetes mellitus [7]. Similarly, prematurity is associated with a higher risk of intracerebral hemorrhage and white matter disease, which could lead to brain growth restriction and cognitive delay in childhood and adolescence [9]. On the other hand, very large head circumferences can be primary and due to increased brain tissue (megalocephaly), which in most cases is familial and benign, or secondary [10]. The latter may be due to multiple diseases such as hydrocephalus, cerebral edema, focal and pericerebral increased fluid collections, thickened calvarium and brain tumors [10]. Despite the great influence of pre-natal factors on brain growth, the post-natal head growth seems to be a better predictor of later IQ than fetal head growth [11], and head growth during the first year of life seems to be more relevant than head growth during the following years [1].

In this scenario, head circumference has been described as the most simple and inexpensive tool to assess the development of the central nervous system [12], which means the measurement has applicability even in low-resource settings, where both undernutrition and suboptimal development are more prevalent. However, no consensus exists about the role of head circumference as a tool to identify children at risk of suboptimal development [13], with conflicting evidence reported in the literature.

Given this uncertainty, this systematic review aims to investigate the association between head circumference and intelligence, schooling (academic performance and educational attainment), employment, and income. To reach this goal, we: 1) summarized the overall evidence based on all included articles, then 2) restricted the evidence to a subset of articles that meet minimum quality criteria.

## Methods

### Protocol development

The review was pre-registered on PROSPERO on 8 December 2021 (ID: CRD42021289998), prior to commencing data extraction.

### Eligibility criteria

A full list of eligibility criteria is provided in Table 1. We initially piloted the eligibility criteria using 100 randomly selected articles identified from our search strategy (see below). During piloting, two researchers independently screened the articles in three stages: reading of titles, abstracts, and full articles. In case of disagreement, a third researcher assumed an arbiter role. This piloting resulted in a clarification of the exposure description: head circumference must be assessed as the variable of interest rather than assessed as a confounder.

**Table 1:** Eligibility criteria

Inclusion criterion	Exclusion criterion
**Study Design**	
• Cohort, case–control, or cross-sectional studies	• Review article, case report or case series, clinical trial, or ecological study
**Population**	
• Children and adults of the general population or • Sample limited by sex, geographic region, or socioeconomic status or • Studies with siblings/twins or • Studies of children with low birth weight, prematurity or small for gestational age	• Other special populations of the mother or children defined by health criterion not mentioned in the inclusion criteria^a^
**Exposure**	
• Head circumference assessed as the variable of interest and • Head circumference measured before the age of 20 years	• Head circumference investigated as a confounder or • Head circumference evaluated through imaging or autopsy
**Outcome**	
• Intelligence or academic performance: independently of age and test or • Educational attainment, employment or income measured after the age of 16 years	• Evaluation of other domains not directly related to intelligence, such as psychomotor development and attention

^a^The study could have included any cause of abnormal head circumference in the general population (including diseases such as congenital infection), but the sample could not have been restricted to participants who presented any specific disease

### Search strategy

We searched five bibliographic databases to identify relevant published studies: PubMed, Web of Science, PsycINFO, LILACS, and CINAHL. Our search included terms related to head circumference and at least one of the following outcomes: intelligence, schooling, employment, or income. No limitations were applied regarding publication date or language. We additionally searched two grey literature sources for unpublished reports: WHO IRIS (International Repository for Information Sharing) and UNICEF Office of Research – Innocenti. For a full list of search terms used, see the Additional file 1.

### Selection and data extraction

Title and abstract screening, full text screening and data extraction were performed by two independent reviewers, using *Mendeley* and *Google forms*. Disagreements were resolved by consultation with a third researcher.

### Risk of bias assessment

We applied the Joanna Briggs Institute (JBI) and Critical Evaluation Tools [14] for risk of bias assessment. The JBI tools are designed for specific study designs, with specific checklists for cohort, case–control, and cross-sectional studies. Each checklist presents a predetermined set of items which should be answered with a response of “yes,” “no,” “unclear,” or “not applicable”. We provided a quantitative score for each item: 0 for “no”, 1 for “unclear” and 2 for “yes”. The item scores were then tallied to provide a total score [15]. Since the number of items varied accordingly to study design, the overall quality score was presented as a proportion: the total score obtained out of the highest score possible in the corresponding checklist. Two researchers applied the tool independently and disagreements were resolved via discussion. The articles' quality assessments using JBI tools are available in the Supplementary Materials (see Additional file 2).

### Minimum quality criteria used for restricted analysis

After the inclusion of the articles accordingly to the protocol, we identified that most articles presented severe limitations that compromised the confidence of the findings. Therefore, we defined criteria à posteriori with which the evidence was narrowed down to ensure a minimum quality of results. While this change in quality assessment was a protocol deviation, we believed it was necessary and sensible to allow appropriate appraisal of included studies.

We limited the synthesis to articles which presented all of the following criteria: 1) a representative sample of a well-defined population group; 2) measurements of the head circumference standardized for age and sex or inclusion of these variables in the adjusted analysis; 3) inclusion of socioeconomic level and maternal IQ or maternal schooling in the adjusted analysis; 4) non-inclusion of well-defined mediators (i.e., schooling, intelligence, or subsequent head circumference measured between exposure and outcome of interest) as confounders in the adjusted analysis; 5) a measure of association between head circumference and the outcome of interest.

The first criterion was established because some studies included a sample that was difficult to interpret even for the hospital or clinic where the participants were selected. This was the case of the study conducted by Kitchen and collaborators [16], which selected a group of children with birth weight between 500 and 1500 g and a control group with birth weight larger than 2500 g. However, the two groups were combined for the analyses, making it not possible to define for which population the effect measure obtained is valid.

Similarly to other anthropometric measurements, head circumference is highly dependent on age and sex; the growth curves proposed by the World Health Organization (WHO) and Centers for Disease Control and Prevention (CDC) are stratified for these variables [17]. Consequently, the second criterion addresses the need to adjust the head circumference measure or the final model for age and sex.

Previous studies showed that head circumference growth is greater in children whose mother completed higher education or from a higher social class [18]. Furthermore, a large proportion of IQ variance is explained by maternal education and IQ [11], and socioeconomic level is considered as one of the main predictors of low IQ in childhood [2]. Therefore, the evaluation of socioeconomic level and maternal IQ or education should be considered mandatory when examining the effects of any predictor of intelligence, and lack of adjustment for these factors may bias estimates and promote spurious associations [11].

Because we are interested in the total effect of the head circumference on the outcome of interest, another requirement was the non-inclusion of well-defined mediators in the adjustment model, under risk of underestimating the measure of association. Finally, a measure of association or effect, such as risk ratio, prevalence ratio, odds ratio, and risk difference, should have been calculated.

The articles' assessments using the minimum quality criteria used for restricted analysis are available in the Supplementary Materials (see Additional file 3).

### Results synthesis

Evidence analysis was conducted in two stages: 1) Summary of overall evidence: an overview of all included articles, and the main findings for each outcome of interest (*N* = 115); and 2) analysis restricted to a subset of articles, based on the minimum quality criteria (*N* = 21). Owing to the large heterogeneity of findings and the small number of included articles for some of the outcomes, we did not perform a meta-analysis. Even for those outcomes with a larger number of studies, there is a high heterogeneity in terms of study population, outcome and exposure assessment, and measure of association. We conducted a vote counting based on the direction of effect [19] stratified by outcome and populational group. If a larger head circumference or a larger rate of growth of head circumference was associated with better outcomes, the direction of effect was positive; if a larger head circumference or a larger rate of growth of head circumference was associated with poorer outcomes, the direction of effect was negative. The definition of “larger” head circumference followed the criteria adopted by the authors of each study. For example, Camargo-Figueira et al. found that head circumferences lower than 2 standard deviations (SD) below the mean at a single-point during the first year of life were associated with low IQ at 6 years (OR 1.7) [2]. Larger head circumferences (i.e., 2 SD below the mean or larger) were associated with better outcomes, so the direction of effect was positive. Similarly, Malacova and colleagues reported that one additional percentile of head circumference at birth was associated with an increase of 0.46 points in reading scores at third grade [20]. Because the beta reported was positive, the direction of effect was also positive. We reported given direction of effect if 70% or more of the outcomes in an article presented the same direction and no clear effect otherwise [21]. For instance, Gale and collaborators reported 7 different measures of association. Because larger head circumference was associated with better outcomes in 6 of them (85.7%) [22], the direction of effect reported for this study was also positive.

It is important to note that the measure of association of each study, but not its p-value, was considered in this method. This decision was based on the current guidelines, which highlight the need to avoid inappropriate strategies such as vote counting based on statistical significance [19, 23].

Analyses were performed in R version 4.2.2.

## Results

### Summary of overall evidence

#### Study selection and characteristics

**Fig. 1:**
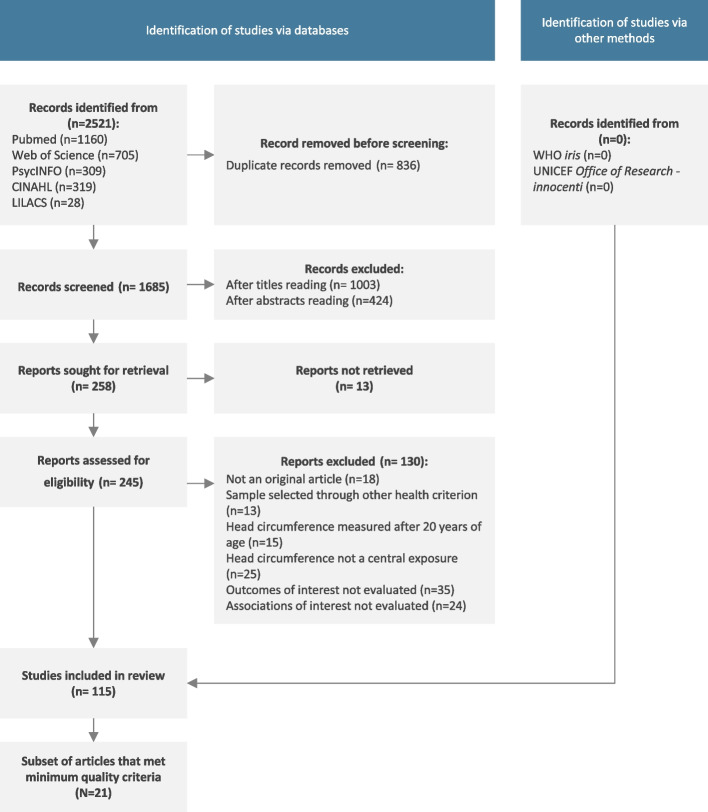
PRISMA Flow chart

The database searches yielded 2,521 articles, 836 of which were duplicates. Of the 1,685 records screened on title and abstract, 115 studies were included in the review. No additional relevant reports were identified from the grey literature (Fig. 1).

**Fig. 2:**
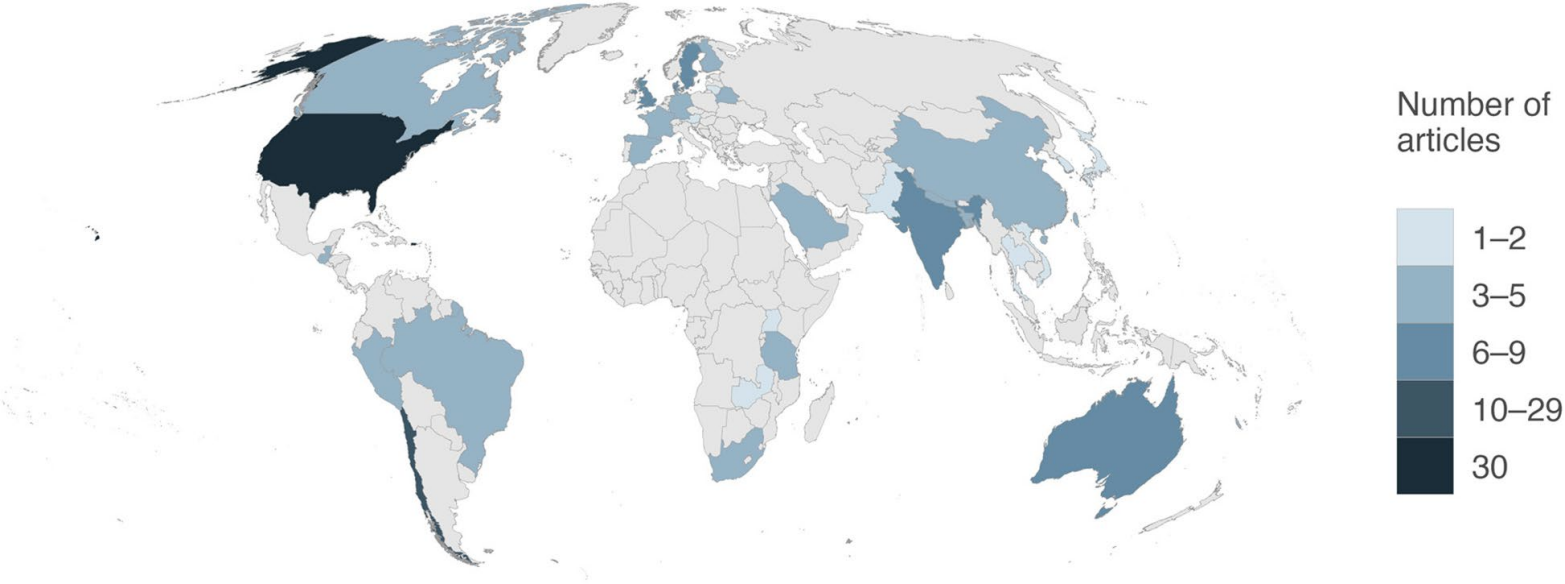
Global distribution map of the included articles according to the country where the data were collected

Figure 2shows a global distribution map of the 115 included articles according to the country where the data were collected. Twenty-three studies investigated the population from low-and-middle income countries [24].

The most frequent study designs were cohort (90 articles) and cross-sectional studies (23 articles). Only one case–control study was included. We were not able to classify one study because of the lack of information provided [25].

We identified large heterogeneity between studies. Most articles evaluated head circumference cross-sectionally at a single time point, but some investigated critical periods based on head growth between successive time points. Studies measured head circumference at different ages: at birth (68 articles), up to two years (54 articles), and between three and 20 years (57 articles). The total number of studies exceeds 115 (which is the number of articles included) because some studies evaluated head circumference more than once during the life course.

Concerning the outcomes of interest, 87 studies looked at intelligence/cognition only, 11 at both intelligence/cognition and academic performance, 13 at academic performance only, 2 at both academic performance and employment, and 2 at educational attainment. No article evaluated the association between head circumference and income.

Almost half of the included studies presented high-risk of selection bias and twenty-five studies conducted no adjustment for confounding. We also identified inaccurate reporting of data collection in some studies, particularly in the exposure measurement process. Reporting who performed the measure, which equipment was used, and if a standard technique was adopted is essential to evaluate information bias; however, few studies included this information [13, 18, 26, 27].

Regarding the list of minimum quality criteria, 108 studies had a sample which were representative of a well-defined group, 33 studies included socioeconomic level and maternal IQ or maternal schooling in the adjusted analysis, 95 studies did not inappropriately include mediators (i.e., schooling and intelligence tests) in the adjusted analysis, 78 studies adjusted the head circumference or the final model for age and sex, and 81 calculated a measure of association between head circumference and the outcome of interest. From the 115 studies included in this review, 21 fulfilled the minimum quality criteria.

Of the 21 studies which were included in the restricted analysis, 20 studies investigated if early measures of head circumference predict later measurements of the outcome, and 5 studies measured head circumference and the outcome at the same timepoint within childhood. The sum exceeds 21 because 4 studies evaluated the association both longitudinally and cross-sectionally. The average number of years between assessment of head circumference and the later follow up assessment of the outcome was 8.5 years (from 1.2 to 20.6 years), and the average age at assessment for the cross-sectional analyses was 6.2 years of age (from 2 to 17 years).

Characteristics of included studies, including details on exposure and outcomes measurements, confounders and main results are provided in the Additional file 4; a summary of findings is shown in Table 2, where all included articles are referenced accordingly with which finding it is relevant for.

**Table 2:** Summary of findings based on all included articles

#	**Main findings**	**Relevant papers**
**Relationship between head circumference and outcomes of interest**		
1	Larger head circumferences are associated with higher levels of intelligence	Álamo-Junquera et al., [28]^a^ Bakhiet et al., [29] Batterjee et al., [30] Bergvall et al., [9]^a^. Bergvall et al., [31]^a^ Boynton et al., [32] Broekman et al., [33] Camargo-Figuera et al., [2]^a^ Camp et al., [34] Caputo et al., [35] Christian et al., [36] Dolk et al., [37] Dupont et al., [38]^a^ Eriksen et al., [11] Ferrer et al., [27]^a^ Flensborg-Madsen et al., [1] Gale et al., [39] Gale et al., [18]^a^ Gale et al., [22]^a^ Gampel et al., [40] Han et al., [41] Hein et al., [42] Heinonen et al., [43] Huang et al., [44] Ivanovic et al., [45] Ivanovic et al., [46] Ivanovic et al., [47] Ivanovic et al., [48] Jaekel et al., [49] Jensen et al., [50] Kirkegaard et al., [51]^a^ Kitchen et al., [16] Klein et al., [52] Koshy et al., [26]^a^ Kroupina et al., [53] Lee et al. [54]^a^, Lei et al., [55]^a^ Lewis et al., [56] Lira et al., [57] Lundgren et al., [58] Lundgren et al., [59] McCall et al., [60]^b^ Miller et al., [61] Muhoozi et al., [11]^b^ Nelson et al., [62] Nicolaou et al., [63]^b^ Park et al., [64] Petersson et al., [65] Pongcharoen et al., [66]^a^ Raikkonen et al., [67] Raikkonen et al., [68] Reolon et al., [69]^a^ Rose et al., [70] Rushton et al., [71] Sandstead et al., [25]^b^ Scharf et al., [72] Sells et al., [73]^b^ Silva et al., [74] Silventoinen et al., [75] Smithers et al., [76] Strauss et al., [77] Veena et al., [78]^a^ Weinberg et al., [79] Wrigh et al. [80]
2	Larger head circumferences are associated with higher levels of academic performance	Bach et al., [81]^a^ Gampel et al., [40] Ivanovic et al., [82] Ivanovic et al., [45] Ivanovic et al., [48] Ivanovic et al., [83] Ivanovic et al., [84] Li et al., [85] Malacova et al., [20]^a^ Sells et al., [73] Smith et al., [86] Toro Diaz et al., [87] Wright et al. [80]
3	Larger head circumferences are associated with higher levels of educational attainment	Ivanovic et al., [88]Pandey et al. [89]
4	Larger head circumferences are associated with an improvement in employment	Dekhtyar et al., [90] Ivanovic et al. [47]
**Critical periods**		
5	The critical period for head growth seems to be the first 1000 days of life	Gale et al., [22]^a^ Heinonen et al., [43] Hickey et al., [91] Kan et al., [82]^a^ Kirkegaard et al., [51]^ab^ McCall et al., [60]^ab^ Pongcharoen et al. [66]^a^
**Nonlinearity of association**		
6	The positive association between head circumference and intelligence is more apparent among people with smaller head circumferences than among those with average-sized heads	Álamo-Junquera et al., [28]^a^ Heinonen et al., [43] Kirkegaard et al., [51]^a^ Koshy et al. [26]^a^
7	There seems to be a weak or no association between head circumference and intelligence when head circumference values fall within a range that is higher than 1 to 1.5 SD above the mean	Álamo-Junquera et al., [28]^a^ Kirkegaard et al. [51]^a^
8	Additional gains in head circumference after the 98th percentile (very large head circumference) are associated with lower levels of intelligence	Álamo-Junquera et al., [28]^a^ Heinonen et al., [43] Lewis et al., [56]^b^ Petersson [65]
**High-risk subpopulations**		
9	Larger head circumferences in premature babies seem to be associated with higher levels of intelligence	Belfort et al., [93]^b^ Bergvall et al., [9]^a^ Cooke et al., [94]^b^ Do et al., [95] Guellec et al., [96] Hickey et al., [91]^ab^ Jaekel et al., [49] Kan et al., [92]^ab^ Kuban et al., [97] Leppanen et al., [98] Lidzba et al., [99] Neubauer et al., [100] Raghuram et al., [101] Raz et al., [102] Raz et al., [103] Selvanathan et al., [104] Yu et al. [105]
10	Larger head circumferences in premature babies are associated with higher levels of academic performance	Charkaluk et al., [106] Guellec et al., [96] Hickey et al., [91]^a^ Kan et al. [92]^a^, Roberts et al. [107]

^a^This study fulfilled the criteria used to restrict the analysis to a subset of articles (*n* = 21)

^b^This study provided evidence against the finding (*n* = 12)

#### Head circumference associations with intelligence in general population

Sixty-seven studies evaluated the association between head circumference and intelligence in the general population. Most used the Wechsler Intelligence Scale [108] or the Bayley Scales of Infant Development [109]. Most studies found positive association between head circumference at a single time point or the rate of head growth and intelligence later in life. In this setting, an important issue to be addressed is the evidence of nonlinearity of this association [26, 28, 43, 51, 56, 65], which is going to be discussed in more detailed in the Sect. " Nonlinear associations with head circumference"*.*

Koshy et al*.*found that head circumference at 2 years more than 3 SD below the mean, but not between 2 and 3 SD below the mean, was a predictor of lower intelligence levels in childhood [26]. Additionally, Alamo-Junquera and colleagues evaluated exposure as a continuous variable and stratified the analysis for head circumference at birth [28]. For children with head circumference below the 10th percentile (*n*= 170), the authors reported that an increase of 1 mm in head circumference was associated with an average increase of 0.47 points (95% CI 0.00 to 0.94) in the Bayley Scale at 14 months [28]. Nonetheless, the same increase in head circumference above the 90th percentile group (*n*= 128) was associated with a decrease of 0.38 points (95% CI -0.93 to 0.18) in the Bayley Scale [28].

Two other studies investigated large, as opposed to small, head circumference for age as exposure [56, 65], using the 98th percentile as a cutoff point. The first study investigated the head circumference at a single point between 3 and 12 years and found no association. It presented small sample size, poor description of sampling and lack of adjustment for confounding [56]. The second found that large head circumference at birth was associated with an increase of 32% in odds of low intellectual performance at military enrollment. Although its sample size was large (*n*= 144,273) and representative of men born in Sweden in the study period, the measure of association was not adjusted for confounding factors [65].

#### Head circumference associations with intelligence in high-risk subpopulations

Thirty seven studies investigated the association in populations of high-risk children, such as premature babies [9, 49, 91–105, 110], babies with low birth weight [16, 35, 49, 57, 98, 99, 110–124] and small for gestational age [125–127]. From those studies, twelve focused on very premature (less than 32 weeks) [49, 93, 94, 96, 98–100, 102, 104, 105, 110] and four on extremely premature babies (less than 28 weeks) [91, 92, 97, 101, 110]. Though most articles found positive association between head circumference and later intelligence in premature babies [49, 95–105, 110], 2 of 3 studies which met the minimum quality criteria presented no clear effects [91, 92]. ﻿Kan et al. found that head growth in the first 2 years, but not at birth nor from 2 to 8 years, was associated with IQ at 8 years [92]. Hickey et al*.*did not investigate head circumference at birth, but also found that head growth in the first 2 years, but not from 2 to 8 years, was associated with improved cognition [91]. Both studies included extremely premature babies.

On the other hand, a study conducted by Bergvall et al*.*calls attention to the interaction effect between head circumference and gestational age [9]. The authors found positive association between head circumference at birth and intelligence at 18 years, with stronger association in premature (less than 37 weeks) than term babies [9].

#### Head circumference associations with academic performance

Academic performance was evaluated using different criteria: national exams, schooling appropriate for age, school difficulties reported by parents, teachers or educational registers, school grades, standardized tests such as the *Wide Range Achievement Test-3* [128] and the *Woodcock Reading Mastery*Test [129], or specifically developed tests for the study. Fourteen articles studied the general population [20, 40, 45, 48, 73, 80, 81, 83–87, 90, 130]. Premature babies [91, 92, 96, 106, 107], low birth weight [107, 114–116] and small for gestational age [127, 131] were also investigated. Most studies found positive associations between head circumference at a single point in time or the rate of head growth and academic performance [20, 40, 45, 48, 73, 80, 81, 83–87, 90–92, 96, 106, 107, 131].

#### Head circumference associations with educational attainment

Two studies investigated educational attainment in the general population [88, 89]. Pandey et al*.*found positive but not statistically significant association between both head circumference at birth and head growth in the first 6 months of life and total years of education at 26–32 years [89]. However, the final sample corresponded to a small fraction of the original cohort (19%) and the complete case analysis included only 37% of the final sample [89]. Ivanovic et al*.*evaluated education situation at 18 years, classified as graduated in high school, delayed, dropout and not located [88]. The outcome was collected 12 years after the measurement of head circumference at a single-point between 5 and 9 years. Despite adjusting for intellectual ability and schooling performance, they found a positive association [88].

#### Head circumference associations with employment

Two cohort studies evaluated employment as the outcome of interest in the general population [47, 90]. Dekhtyar et al*.*applied the Standard International Occupational Prestige Scale to investigate the longest-held occupation in adulthood [90]. The scale emphasizes subjective perceptions of social rewards inherent to different occupations and was developed from averaging scales across 60 countries. On the other hand, Ivanovic et al*.*evaluated job status, a classification that also included educational attainment [47]. Data was collected six years after high school graduation. Both studies found positive associations between head circumference at a single-point and the outcome but lacked adjustment for confounders [47, 90].

#### Nonlinear associations with head circumference

Evidence strongly points to the nonlinearity of the association of interest [26, 28, 43, 51, 56, 65]. In this context, some articles used methodology other than exposure categorization to explore nonlinear relationships between head circumference and intelligence. Kirkegaard and colleagues conducted additional analysis through generating restricted cubic splines. They found positive association between head circumference-for age z-scores and IQ at 5 years, which seemed to plateau when head circumference values were higher than 1 to 1.5 SD [51]. Similarly, Heinonen et al*.*modeled squared terms and polynomial contrasts to test the potential U-shaped relationships [43]. The authors reported that both small and large head circumference at a single-time point, and slower and faster head growth, were associated with lower levels of visual-motor integration at 56 months. However, vulnerable children presented higher follow-up rates and the adjustment model did not include socioeconomic level. Results for general reasoning were not shown [43].

#### Critical periods for head growth

An important issue to be addressed is the evaluation of critical growth periods: evidence suggests that the association between head growth and intelligence closes after the second year of life. Because of the large heterogeneity, we focused on comparison within studies rather than between studies. Moreover, all articles discussed in this section used intelligence as outcome, and did not consider other outcome measures. Two of four studies which adjusted the exposure for its previous measures found negative but non-statistically significant association between head growth after the first [66] or second [43] year of life and intelligence. One study presented results in both directions: i.e., a positive association for head growth between 1 and 4 years and a negative association for head growth between 4 and 8 years [22]. Two studies found positive association [18, 51]; however, one did not adjust head growth for its previous measures [18], and another adjusted only for head circumference at the first year [51].

Comparison between head circumference at birth and head growth during the first two years of life is less clear in the literature [1, 22, 27, 43, 51, 69, 72]. Three studies found stronger association for head growth during the first two years of life [1, 51, 69]. However, all used secondary data for head circumference at birth and primary data for subsequent measurement [1, 51, 69]. Two studies found quite similar results [27] or findings in both directions [22], and one reported stronger association for head circumference at birth [43].

Finally, Scharf et al*.*evaluated body size as a growth construct through monthly measures from study enrollment (means 7 days of life) to 2 years [72]. The study included children from four low-and-middle-income countries and presented a detailed methodology description. They reported two important findings: a) head growth in the second year of life was more positively associated to cognitive scores than constructs in the first year of life and b) head circumference was more strongly associated with cognitive skills than length or weight for age. However, since their aim was to explore a predictive model rather than establish causal relationships, adjustment for confounding was not performed [72]. Zhu and colleagues found similar results in China after adjustment for important confounders. For IQ at 10–14 years, they observed that the point estimate of conditional head circumference growth increased up to 18 months of age and then decreased during the post-18 months [13].

### Analysis restricted to a subset of articles that met minimum quality criteria

#### Vote counting based on direction of effect

**Fig. 3:**
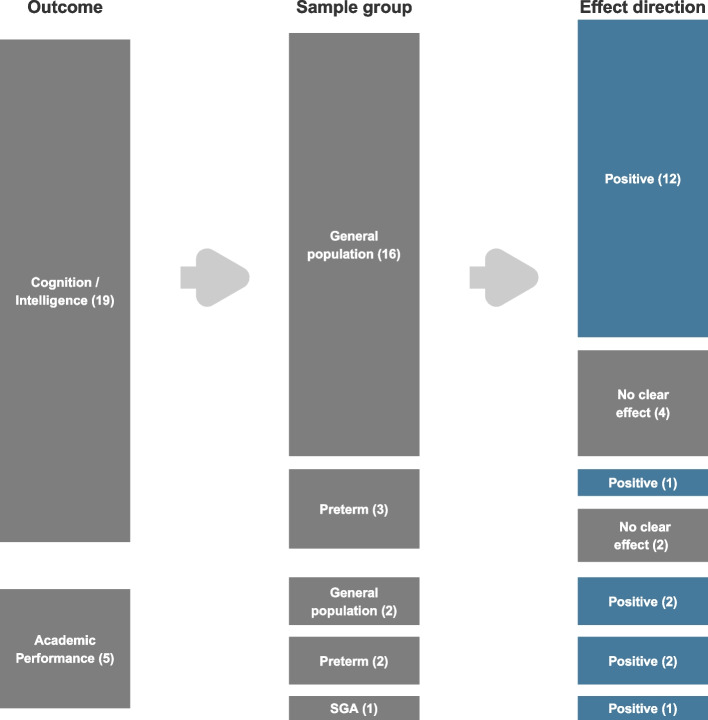
Analysis restricted to a subset of articles that met minimum quality criteria: vote counting based on direction of effect by outcome and sample group

Twenty one of 115 included articles fulfilled the criteria for this restricted analysis (Fig. 1). Figure 3 shows vote counting based on the direction of effect by outcome and sample group. The number of studies does not sum to 21 because some studies investigated more than one outcome or population group.

The number of studies does not sum to 21 because some studies investigated different outcomes or sample groups.

There was evidence that both head circumference at a single point in time and head growth is associated with intelligence in the general population, with 12 of 16 articles showing positive results (75%). However, the association is not consistent in premature babies, with 1 of 3 articles showing positive results (33.3%) and the remaining (66.3%) showing no clear effect.

Head circumference was positively associated with academic performance in all investigated samples: general population (2 of 2 articles), premature babies (2 of 2 articles) and small for gestational age (1 of 1 article). Both head circumference at a single point in time and head growth were investigated.

#### Additional information on confounders

In addition to some confounders that were considered as essential across articles (i.e., socioeconomic level and maternal IQ or maternal schooling), some articles also included maternal variables: e.g. age [9, 18, 20, 22, 27, 28, 31, 51, 55, 131], height [44, 66], weight [44], BMI [27, 28, 51], parity or number of siblings [36, 76, 81], family structure or cohabitation [9, 31, 81], marital status [20, 55], smoking [2, 28, 51, 55, 76, 81], high blood pressure [55, 81], diabetes [81], history of depression or other psychiatric illnesses [18, 22, 81]; and participant variables: skin color or ethnicity [2, 20, 55], weight [20, 31, 44, 51], length or height [9, 20, 26, 31, 51] and BMI [31]. Some articles also included breastfeeding [2, 18, 22, 51, 55] or stimulation [22], i.e., the quality and quantity of stimulation and support available in the home environment. Moreover, other articles conducted different strategies to adjust for confounding. Three articles investigated the association between head circumference and intelligence in twins [54, 79] or siblings [31]. They found positive association in intra-pair analysis [54, 79], which allows the adjustment for shared genetic and environmental factors.

## Discussion

In the general population, small head circumference in the first two years are associated with lower levels of later intelligence [2, 9, 18, 26–28, 31, 38, 69, 78], whereas a weak or no association was reported for head circumference values higher than 1 to 1.5 SD above the mean [26, 28, 43, 51]. Very large head circumference has been scarcely studied, but it also seems to be associated with lower levels of later intelligence [28, 43, 65]. Further research is needed for a deeper understanding of the whole distribution of head circumference considering the non-linearity of its association with later outcomes.

Even though the association between the different patterns of head circumference and later outcomes were not completely understood, the present findings agree with the current recommendation: head circumference should be measured at birth and repeatedly measured throughout infancy and early childhood, particularly in the first 2 years of life [105]. The early recognition of the extremes of head circumference distribution, particularly less than 2 SD below the mean and above the 98th percentile, can lead to referrals to pediatric specialist physicians and provision of family-centered early intervention services [12]. Furthermore, this recommendation is particularly relevant in low-and-middle-income countries. Since recording head circumference measurements is a relatively inexpensive tool capable of identifying children at risk of worse outcomes, it could be implemented in low-resource settings, where both the exposure and the outcomes studied in this review are more prevalent [2, 17]. Large surveys which have been widely conducted in low-and-middle-income countries, such as MICS (Multiple Indicator Cluster Survey) [107] and DHS (Demographic and Health Survey) [108], include only weight and height in the anthropometric measures collected in children under two years of age. Although the majority of included articles came from high-income countries, 20% of the studies was conducted in low-and-middle-income countries, with similar results between the two groups.

In this review, few articles included breastfeeding or stimulation measured after the head circumference measurement as confounders in the analysis model, but no study investigated these variables as interaction factors or in a mediation analysis. Besides the risk of underestimating the measure of association, the current literature has missed the opportunity to investigate the potential consequences of these mitigating factors on the negative effect of small head circumference on later outcomes. The effect of breastfeeding on later outcomes is well-defined in the literature: the duration of total breastfeeding was positively associated with higher IQ, schooling, and income at 30 years [132]. One of the possible pathways which could explain this association is the presence of the docosahexaenoic acid in the breast milk, a component of the central nervous system involved in cell membrane biogenesis, maintenance of cell fluidity, neurogenesis, neurotransmission, and protection against oxidative stress [133]. Additionally, evidence suggests that exclusively breastfed infants present faster rates of head growth in the first month than partially or non-breastfed infants [57], differences that seem to persist up to five years-old [134].

Globally, home environments characterized by responsive caregiving and learning opportunities have been positively associated with child development [136]. Moreover, the critical period to these effects seems to extend throughout childhood and adolescence, suggesting the involvement of mechanisms rather than brain growth [22, 135]. In this context, data from two middle-income countries suggests that early nurturing home environments protect young children against effects of early adversities on adolescent IQ [135]. This study investigated early adversities as a cumulative index which included variables such as income household, maternal schooling, birthweight, and length-for-age [135]. Further research is needed to confirm if the moderation effect of stimulation also extends to the association between head circumference and later outcomes.

Regarding premature babies, the association between head circumference and later intelligence is less clear [91, 92]. Although the overall evidence points to a positive association, two of three studies which fulfilled the minimum quality criteria found no clear results. However, both studies focused on extremely preterm babies and hence were particularly susceptible to survival bias [91, 92]. Defining vulnerability on the basis of preterm birth alone does not account for overlapping between preterm birth (i.e., born too soon) and FGR (i.e., born too small), which represents an increased risk of both mortality and long-term morbity [136]. Some of these babies probably suffered from asymmetric FGR, when baby's system has compensated for some problems in its environment by shifting more of its blood flow to the head and brain. As a result, the head circumference is preserved, length is somewhat affected, and weight is compromised to a greater degree [7]. When these compensatory mechanisms failed, the head circumference is also compromised. Symmetric FGR begins early in pregnancy as a consequence of congenital infections, chromosomal abnormalities, or sustained decrease in nutrient supply, and it is associated with moderate and severe cognitive impairment [OR 1.65 (CI95%:1.01;2.71) and OR 2.61(IC95%: 1.46;4.68)] [7].

Finally, larger head circumferences seem to be associated with better academic performance in both the general population and premature babies. The evidence for educational attainment and employment is more limited, but the association also seems to be positive. Exploring these outcomes in further research is important because in comparison with IQ, education provides a more comprehensive set of skills that are essential for success and fulfillment in day-to-day life, including employment and achieved income in life.

Even though our review included a relatively large number of articles, less than 20% (21 out of 115 studies) constituted the subset of articles with minimum quality to which the analysis was restricted (Fig. 3). The main limitations of the included articles were severe selection bias and lack of adjustment for confounding. Because socio-economic level, parental schooling and maternal IQ have been stated as the most important predictors of IQ in the childhood [2], these covariates must be considered mandatory when examining the effects of any intelligence predictor [11]. Additionally, since head circumference is highly depended on age and sex, taking these variables into account is also crucial. It is important to highlight that, although men have larger head circumferences than women, there is no differences in average IQ between sexes [137].

Another limitation was the inappropriate adjustment for mediator variables. The failure to consider a causal diagram in the analysis led some studies to inadvertently adjust for schooling [1, 39, 42, 48, 53, 138] or previous intelligence tests [1, 48]. Since we are interested in the total effect of head circumference, this approach could cause bias toward the null. Other limitations were attrition bias, non-reporting of loss to follow-up information, failure in considering the non-linearity of association in the analysis and non-adjustment for previous head circumference measure when evaluating head growth.

Our study has some strengths. We used a comprehensive list of search terms and inclusion criteria and no date or language limitations, which increased the sensitivity of our literature search. At the same time, we explored the limitations of individual studies, and restricted the analysis to a subset of articles with minimum quality. Finally, we identified gaps in the literature and provided recommendations to further research.

On the other hand, we are aware that our study also has limitations. The large heterogeneity between studies prevented us from performing a meta-analysis; we used vote counting based on direction of effect due to inconsistency in the effect measures across the studies. Limitations inherent in this method included lack of information on the magnitude of effects, non-accountability for differences in the relative sizes of the study and difficulty to assess consistency of effects. Regardless, the formal application of this method makes the synthesis process more transparent and reproducible and can prevent the use of methods that would be inappropriate in this context, such as vote counting based on statistical significance [19, 23].

## Conclusions

Larger head circumferences are generally associated with increase in intelligence and academic performance, but there is evidence of non-linearity in those associations. The role of head circumference measurement seems to diminish after two years of age. Despite limited evidence, larger head circumferences also seem to be associated with improvement in educational attainment and employment. No article investigated the association between head circumference and income.

Identifying a group of children in higher risk for worse outcomes by a simple and inexpensive measurement intervention tool could provide an opportunity to mitigate these negative effects. Further research is required to better understand the association in premature babies, as well as to explore the role of variables such as stimulation in early childhood, breastfeeding, and socioeconomic level as interaction factors. Studies should consider the non-linear relationship between head circumference and intelligence in the analysis.

## Supplementary Information

Additional file 1. Search terms used in the systematic search. The file presents a full list of search terms used in the systematic searches, by data base.

Additional file 2. Articles' quality assessments using JBI tools. The file shows the articles' quality assessments using JBI (Joanna Briggs Institute) tools.

Additional file 3. Minimum quality criteria used for restricted analysis. The file shows the minimum quality criteria used for restricted analysis applied in all included articles.

Additional file 4. Details of all included articles. Details of the 115 included articles are provided in this file.

## Data Availability

All forms, datasets and analytic codes used in the review are available upon request at deborbamarina@gmail.com.

## Abbreviations

IQ: Intelligence quotient
FGR: Fetal growth restriction
IRIS: International Repository for Information Sharing
JBI: Joanna Briggs Institute
WHO: World Health Organization
CDC: Centers for Disease Control and Prevention
SD: Standard deviations
MICS: Multiple Indicator Cluster Survey
DHS: Demographic and Health Survey
