# Supplementary material for: Head circumference and intelligence, schooling, employment, and income: a systematic review
Source: BMC Pediatr. 2024 Nov 7;24:709. doi: 10.1186/s12887-024-05159-2 (PMC11542250; doi:10.1186/s12887-024-05159-2)
Supplement: Supplementary file 1 — Additional file 1. Search terms used in the systematic search. The file presents a full list of search terms used in the systematic searches, by data base. [file 12887_2024_5159_MOESM1_ESM.pdf]

Systematic searches were conducted on 18 September 2021 and updated on 29 March 2023. No date or language limitations were used, no filter was applied.

Pubmed:

((((((("Cephalometry") OR ("head circumference")) OR ("cephalic perimeter")) OR ("occipitofrontal circumference")) OR ("Anthropometric cranial measures")) OR ("Head measurements")) AND ((((((((((("Intelligence") OR ("Intelligence quotient")) OR ("Intellectual performance")) OR ("Academic Performance")) OR ("Schooling")) OR ("Educational Status")) OR ("Economic Status")) OR ("Income")) OR ("Wealth")) OR ("socioeconomic factors")) OR ("Employment")) OR ("Cognition"))

Web of science:

#2
(((((((((((ALL=("Intelligence")) OR ALL=("Intelligence quotient")) OR ALL=("Intellectual performance")) OR ALL=("Academic Performance")) OR ALL=("Schooling")) OR ALL=("Educational Status")) OR ALL=("Economic Status")) OR ALL=("Income")) OR ALL=("Wealth")) OR ALL=("socioeconomic factors")) OR ALL=("Employment")) OR ALL=("Cognition"))

1
((((((ALL=("Cephalometry")) OR ALL=("head circumference")) OR ALL=("cephalic perimeter")) OR ALL=("occipitofrontal circumference")) OR ALL=("Anthropometric cranial measures")) OR ALL=("Head measurements"))

#1 AND #2

PsycINFO:

((**Any Field**: ("Cephalometry")) *OR* (**Any Field**: ("head circumference")) *OR* (**Any Field**: ("cephalic perimeter")) *OR* (**Any Field**: ("occipitofrontal circumference")) *OR* (**Any Field**: ("Anthropometric cranial measures")) *OR* (**Any Field**: ("Head measurements"))) *AND* ((**Any Field**: ("Intelligence")) *OR* (**Any Field**: ("Intelligence quotient")) *OR* (**Any Field**: ("Intellectual performance")) *OR* (**Any Field**: ("Academic Performance")) *OR* (**Any Field**: ("Schooling")) *OR* (**Any Field**: ("Educational Status")) *OR* (**Any Field**: ("Economic Status")) *OR* (**Any Field**: ("Income")) *OR* (**Any Field**: ("Wealth")) *OR* (**Any Field**: ("socioeconomic factors")) *OR* (**Any Field**: ("Employment")) *OR* (**Any Field**: ("Cognition")))

CINAHL:

(S1 AND S2)

S1

“Cephalometry” OR "head circumference" OR “cephalic perimeter” OR “occipitofrontal circumference" OR “Anthropometric cranial measures” OR "Head measurements"

S2
“Intelligence” OR “Intelligence quotient” OR “Intellectual performance” OR “Academic Performance" OR “Schooling” OR “Educational Status” OR “Economic Status” OR “Income” OR “Wealth” OR “socioeconomic factors” OR “Employment” OR "Cognition"

LILACS:

(((((("OCCIPITO-FRONTAL") or "CEPHALOMETRY") or "PERIMETRO CEFALICO") or "head circumference") or "Anthropometric cranial measures") or "Head measurements") [Palavras] and (((((((((("INTELLIGENCE") or "INTELLIGENCE TEST") or "ACADEMIC PERFORMANCE") or "SCHOOLING") or "ECONOMIC STATUS") or "INCOME") or "WEALTH") or "SOCIOECONOMIC FACTORS") or "EMPLOYMENT") or "Educational Status")) or "COGNITION" [Palavras]

WHO IRIS:

“Cephalometry”, "head circumference", “cephalic perimeter”, “occipitofrontal circumference”,

“Anthropometric cranial measures”, or "Head measurements".

UNICEF Office of Research – Innocenti:

“Cephalometry”, "head circumference", “cephalic perimeter”, “occipitofrontal circumference”,

“Anthropometric cranial measures”, or "Head measurements".

All forms, datasets and analytic codes used in the review are available upon request at *deborbamarina@gmail.com*.
