# Supplementary material for: Head circumference and intelligence, schooling, employment, and income: a systematic review
Source: BMC Pediatr. 2024 Nov 7;24:709. doi: 10.1186/s12887-024-05159-2 (PMC11542250; doi:10.1186/s12887-024-05159-2)
Supplement: Supplementary file 2 — Additional file 2. Articles' quality assessments using JBI tools. The file shows the articles' quality assessments using JBI (Joanna Briggs Institute) tools. [file 12887_2024_5159_MOESM2_ESM.pdf]

Checklist for Cohort Studies

Joanna Briggs Institute Critical Appraisal tools

Code	Observation	Question
c01		Were the two groups similar and recruited from the same population?
c02		Were the exposures measured similarly to assign people to both exposed and unexposed groups?
c03		Was the exposure measured in a valid and reliable way?
c04		Were confounding factors identified?
c05		Were strategies to deal with confounding factors stated?
c06		Were the groups/participants free of the outcome at the start of the study (or at the moment of exposure)?
c07		Were the outcomes measured in a valid and reliable way?
c08	Not applicable	Was the follow up time reported and sufficient to be long enough for outcomes to occur?
c09		Was follow up complete, and if not, were the reasons to loss to follow up described and explored?
c10		Were strategies to address incomplete follow up utilized?
c11		Was appropriate statistical analysis used?

Author	Title	Year	c01	c02	c03	c04	c05	c07	c09	c10	c11	c06	c08
**Alamo-Junquera**	Prenatal head growth and child neuropsychological development at age 14 months	2014	Yes	Yes	Unclear	Yes	Yes	Yes	Yes	Unclear	Yes	Unclear	Yes
**Bach**	Head circumference at birth and school performance: a nationwide cohort study of 536,921 children	2019	Yes	Yes	Unclear	Yes	Yes	Unclear	Yes	Yes	Yes	Unclear	Yes
**Beck**	Prenatal and early childhood predictors of intelligence quotient (IQ) in 7-year-old Danish children from the Odense Child Cohort	2022	Yes	Yes	Unclear	Yes	Yes	Yes	Yes	Yes	Yes	Unclear	Yes
**Belfort**	Infant growth before and after term: effects on neurodevelopment in preterm infants.	2015	Yes	Yes	Yes	Yes	Yes	Yes	Yes	Unclear	Yes	Unclear	Yes

Author	Title	Year	c01	c02	c03	c04	c05	c07	c09	c10	c11	c06	c08
**Bergvall**	Risks for low intellectual performance related to being born small for gestational age are modified by gestational age.	2006	Yes	Yes	Unclear	Yes	Yes	Unclear	Yes	Unclear	Yes	Unclear	Yes
**Bergvall N**	Birth characteristics and risk of low intellectual performance in early adulthood: are the associations confounded by socioeconomic factors in adolescence or familial effects?	2006	Unclear	Yes	Unclear	Yes	Yes	Unclear	Yes	Unclear	Yes	Unclear	Yes
**Brinkis**	Impact of Early Nutrient Intake and First Year Growth on Neurodevelopment of Very Low Birth Weight Newborns	2022	Yes	Yes	Yes	No	Unclear	Yes	Yes	No	Unclear	Unclear	Yes
**Broekman**	The influence of birth size on intelligence in healthy children.	2009	Yes	Yes	Unclear	Yes	Yes	Yes	Yes	Unclear	Yes	Unclear	Yes
**Camargo-Figuera**	Early life determinants of low IQ at age 6 in children from the 2004 Pelotas Birth Cohort: a predictive approach.	2014	Yes	Yes	Unclear	Yes	Yes	Yes	Yes	Yes	Yes	Unclear	Yes
**Camp**	Maternal and neonatal risk factors for mental retardation: defining the 'at-risk' child.	1998	Yes	Yes	Unclear	No	Unclear	Unclear	No	Unclear	Unclear	Unclear	Yes
**Caputo**	An evaluation of various parameters of maturity at birth as predictors of development at one year of life	1974	No	Yes	Unclear	Unclear	Unclear	Yes	No	Unclear	Unclear	Unclear	Yes
**Charkaluk**	Very preterm children free of disability or delay at age 2: predictors of schooling at age 8: a population-based longitudinal study.	2011	Yes	Yes	Unclear	Unclear	Yes	Yes	Yes	Unclear	Yes	Unclear	Yes
**Christian**	Associations between preterm birth, small-for-gestational age, and neonatal morbidity and cognitive function among school-age children in Nepal.	2014	Yes	Unclear	Unclear	Yes	Yes	Yes	Unclear	Unclear	Yes	Unclear	Yes

Author	Title	Year	c01	c02	c03	c04	c05	c07	c09	c10	c11	c06	c08
**Cooke**	Perinatal and postnatal factors in very preterm infants and subsequent cognitive and motor abilities.	2005	Yes	Yes	Unclear	Unclear	Yes	Unclear	Unclear	Unclear	Unclear	Unclear	Yes
**Cooke**	Are there critical periods for brain growth in children born preterm?	2006	Yes	Yes	Unclear	Unclear	No	Unclear	Unclear	Unclear	Unclear	No	Yes
**Dekhtyar**	Associations of head circumference at birth with earlylife school performance and later-life occupational prestige	2015	Yes	Yes	Unclear	Yes	Yes	Unclear	Yes	Yes	Yes	Unclear	Yes
**Do**	Poor Head Growth Is Associated with Later Mental Delay among Vietnamese Preterm Infants: A Follow-up Study.	2020	Yes	Yes	Yes	Unclear	Yes	Yes	Yes	Unclear	Yes	Unclear	Yes
**Dolk**	The predictive value of microcephaly during the first year of life for mental retardation at seven years.	1992	Yes	Yes	No	No	No	Unclear	Unclear	Unclear	Unclear	Unclear	Yes
**Dupont**	The Predictive Value of Head Circumference Growth during the First Year of Life on Early Child Traits.	2018	Yes	Yes	Unclear	Unclear	Yes	Yes	Unclear	Unclear	Yes	Unclear	Yes
**Eriksen**	Predictors of intelligence at the age of 5: family, pregnancy and birth characteristics, postnatal influences, and postnatal growth.	2013	Yes	Yes	Unclear	Yes	Yes	Yes	No	Yes	Yes	Unclear	Yes
**Fattal-Valevski**	Growth patterns in children with intrauterine growth retardation and their correlation to neurocognitive development.	2009	Yes	Yes	Yes	No	No	Unclear	No	No	Unclear	Unclear	Yes
**Ferrer**	Head circumference and child ADHD symptoms and cognitive functioning: results from a large population-based cohort study	2018	Yes	Yes	Yes	Yes	Yes	Yes	Yes	Unclear	Yes	Unclear	Yes
**Flensborg-Madsen**	Early life predictors of intelligence in young adulthood and middle age.	2020	Yes	Yes	Unclear	Yes	Yes	Yes	No	Unclear	Yes	Unclear	Yes

Author	Title	Year	c01	c02	c03	c04	c05	c07	c09	c10	c11	c06	c08
**Frisk**	The importance of head growth patterns in predicting the cognitive abilities and literacy skills of small-for-gestational-age children.	2015	Unclear	Yes	Unclear	Unclear	Unclear	Yes	Unclear	Unclear	Yes	Unclear	Yes
**Gale**	The influence of head growth in fetal life, infancy, and childhood on intelligence at the ages of 4 and 8 years.	2006	Yes	Yes	Yes	Yes	Yes	Unclear	Yes	Unclear	Yes	Unclear	Yes
**Gale**	Critical periods of brain growth and cognitive function in children.	2004	Yes	Yes	Yes	Yes	Yes	Yes	Yes	Yes	Yes	Unclear	Yes
**Gale**	Foetal and postnatal head growth and risk of cognitive decline in old age.	2003	Yes	Yes	No	Unclear	Yes	Yes	Yes	Unclear	Yes	Unclear	Yes
**Gampel**	Short and Long-Term Effects of Compromised Birth Weight, Head Circumference, and Apgar Scores on Neuropsychological Development.	2014	Yes	Yes	Unclear	Unclear	Yes	Yes	Unclear	Unclear	Yes	Unclear	Yes
**Gross**	Newborn head size and neurological status. Predictors of growth and development of low birth weight infants.	1978	Yes	Yes	Unclear	No	No	Yes	Yes	Unclear	Yes	Unclear	Yes
**Guellec**	Intrauterine Growth Restriction, Head Size at Birth, and Outcome in Very Preterm Infants.	2015	Yes	Yes	Unclear	Yes	Yes	Yes	Yes	Unclear	Yes	Unclear	Yes
**Hack**	Very low birth weight infants: effects of brain growth during infancy on intelligence quotient at 3 years of age.	1986	Yes	Yes	Unclear	Yes	Yes	Unclear	Unclear	Unclear	Unclear	Unclear	Yes
**Hack**	Differential effects of intrauterine and postnatal brain growth failure in infants of very low birth weight.	1989	Yes	Yes	Unclear	Unclear	Yes	Unclear	No	Unclear	Yes	Unclear	Yes

Author	Title	Year	c01	c02	c03	c04	c05	c07	c09	c10	c11	c06	c08
**Han**	Functional principal component analysis for identifying multivariate patterns and archetypes of growth, and their association with long-term cognitive development.	2018	Yes	Yes	Unclear	Unclear	Unclear	Unclear	No	Unclear	Yes	Unclear	Yes
**Heinonen**	Prenatal and postnatal growth and cognitive abilities at 56 months of age: A longitudinal study of infants born at term	2008	Yes	Yes	Unclear	Yes	Yes	Yes	Yes	Unclear	Yes	Unclear	Yes
**Hickey**	Extreme prematurity, growth and neurodevelopment at 8 years: a cohort study.	2021	Yes	Yes	Yes	Unclear	Unclear	Unclear	Unclear	Unclear	Yes	Unclear	Yes
**Ivanovic**	Twelve-year follow-up study of the impact of nutritional status at the onset of elementary school on later educational situation of Chilean school-age children.	2008	Yes	Yes	Unclear	Unclear	Yes	Yes	Yes	Unclear	Yes	Unclear	Yes
**Ivanovic**	Impact of anthropometric nutritional parameters on the university selection test in Chile: A multifactorial approach.	2019	Yes	Yes	Yes	Yes	Yes	Unclear	No	Unclear	Yes	Unclear	Yes
**Ivanovic**	Impact of nutritional status at the onset of elementary school on academic aptitude test achievement at the end of high school in a multicausal approach.	2009	Yes	Yes	Yes	Unclear	Unclear	Unclear	Yes	Unclear	Yes	Unclear	Yes
**Ivanovic**	Neuropsychological Parameters Affecting the Academic Aptitude Test (AAT) Achievement at the End of High School in 1996 and Their Impact on Job Status in 2002: A Multifactorial Approach in a Follow-up Study.	2006	Unclear	Yes	Unclear	No	No	Yes	Unclear	No	No	Unclear	Yes

Author	Title	Year	c01	c02	c03	c04	c05	c07	c09	c10	c11	c06	c08
**Jaekel**	Head Growth and Intelligence from Birth to Adulthood in Very Preterm and Term Born Individuals.	2018	Yes	Yes	Yes	Unclear	Yes	Yes	Yes	Unclear	Yes	Unclear	Yes
**Jensen**	Cognitive ability in adolescents born small for gestational age: Associations with fetal growth velocity, head circumference and postnatal growth.	2015	Yes	Yes	Unclear	Yes	Yes	Yes	No	Unclear	Yes		Yes
**Kan**	The association of growth impairment with neurodevelopmental outcome at eight years of age in very preterm children.	2008	Yes	Yes	Yes	Yes	Yes	Yes	Unclear	Unclear	Yes	Unclear	Yes
**Kim**	Cognitive Outcomes of Children with Very Low Birth Weight at 3 to 5 Years of Age.	2020	Yes	Yes	Unclear	Unclear	Unclear	Yes	Unclear	Unclear	Yes	Unclear	Yes
**Kirkegaard**	Associations of birth size, infancy, and childhood growth with intelligence quotient at 5 years of age: a Danish cohort study.	2020	Yes	Yes	Unclear	Yes	Yes	Yes	Yes	Yes	Yes	Unclear	Yes
**Koller**	Patterns of cognitive development in very low birth weight children during the first six years of life.	1997	Yes	Yes	Unclear	No	No	Unclear	No	Unclear	Yes	Unclear	Yes
**Koshy**	Association between head circumference at two years and second and fifth year cognition.	2021	Yes	Yes	Yes	Yes	Yes	Yes	Yes	Unclear	Yes	Unclear	Yes
**Kroupina**	Associations between physical growth and general cognitive functioning in international adoptees from Eastern Europe at 30 months post-arrival	2015	Yes	Yes	Yes	Unclear	Yes	Yes	Yes	Unclear	Yes	Unclear	Yes
**Larroque**	School difficulties in 20-year-olds who were born small for gestational age at term in a regional cohort study.	2001	Yes	Yes	No	Yes	Yes	Yes	Yes	Unclear	Yes	Unclear	Yes

Author	Title	Year	c01	c02	c03	c04	c05	c07	c09	c10	c11	c06	c08
**Lei**	Choosing the Best Newborn Anthropometric Measure Associated With the Risks and Outcomes of Intrauterine Growth Restriction	2015	Yes	Yes	Yes	Yes	Yes	Yes	Unclear	Unclear	Yes	Unclear	Yes
**Leppänen**	Antenatal and postnatal growth and 5-year cognitive outcome in very preterm infants.	2014	Yes	Yes	Yes	Unclear	Yes	Yes	Yes	Unclear	Yes	Unclear	Yes
**Li**	Relative importance of birth size and postnatal growth for women's educational achievement.	2004	Yes	Yes	Yes	Yes	Yes	Yes	Yes	Unclear	Yes	Unclear	Yes
**Lidzba**	Growth in very preterm children: Head growth after discharge is the best independent predictor for cognitive outcome.	2016	Yes	Yes	Yes	Yes	Yes	Unclear	Unclear	Unclear	Yes	Unclear	Yes
**Lira**	Early head growth: relation with IQ at 8 years and determinants in term infants of low and appropriate birthweight.	2009	Yes	Yes	Yes	Yes	Yes	Yes	Yes	Unclear	Yes	Unclear	Yes
**Lorenz**	Indices of body and brain size at birth and at the age of 2 years: relations to cognitive outcome at the age of 16 years in low birth weight infants.	2009	Yes	Yes	Unclear	Yes	Yes	Unclear	Yes	Unclear	Yes	Unclear	Yes
**Lundgren**	Intellectual and psychological performance in males born small for gestational age with and without catch-up growth.	2001	Yes	Yes	Unclear	Unclear	Unclear	Unclear	Yes	Unclear	Yes	Unclear	Yes
**Lundgren**	Short Adult Stature and Overweight Are Associated with Poor Intellectual Performance in Subjects Born Preterm	2011	Yes	Yes	Unclear	Unclear	Yes	Unclear	Yes	Unclear	Yes	Unclear	Yes
**Lundgren**	Birth characteristics and different dimensions of intellectual performance in young males: a nationwide population-based study.	2003	Yes	Unclear	Unclear	Unclear	Yes	Unclear	Yes	Unclear	Yes	Unclear	Yes

Author	Title	Year	c01	c02	c03	c04	c05	c07	c09	c10	c11	c06	c08
**Malacova**	Neighbourhood socioeconomic status and maternal factors at birth as moderators of the association between birth characteristics and school attainment: a population study of children attending government schools in Western Australia.	2009	Yes	Yes	Unclear	Yes	Yes	Unclear	Yes	Unclear	Yes	Unclear	Yes
**Mccall**	Developmental changes in head-circumference and mental-performance growth rates: a test of Epstein's phrenoblysis hypothesis.	1983	Yes	Yes	Yes	No	No	Yes	Unclear	Yes	Yes	Unclear	Yes
**Miller**	Outcomes of children adopted from Eastern Europe.	2009	Unclear	Yes	Unclear	No	No	Yes	Yes	Unclear	Yes	Unclear	Yes
**Nash**	Pattern of growth of very low birth weight preterm infants, assessed using the WHO Growth Standards, is associated with neurodevelopment.	2011	Yes	Yes	Unclear	Unclear	Yes	Yes	Yes	Unclear	Yes	Unclear	Yes
**Nelson**	Head size at one year as a predictor of four-year IQ	1970	Yes	Yes	Yes	Unclear	Unclear	Unclear	No	Unclear	No	Unclear	Yes
**Neubauer**	Poor postdischarge head growth is related to a 10% lower intelligence quotient in very preterm infants at the chronological age of five years.	2016	Yes	Yes	Unclear	No	Unclear	Unclear	Yes	Unclear	Yes	Unclear	Yes
**Nicolaou**	Factors associated with head circumference and indices of cognitive development in early childhood	2020	Yes	Yes	Yes	Unclear	Yes	Yes	Yes	Unclear	Yes	Unclear	Yes
**Ochiai**	Head circumference and long-term outcome in small-for-gestational age infants.	2008	Yes	Yes	Unclear	No	No	Unclear	No	Unclear	Yes	Unclear	Yes
**Pandey**	Childhood Head Growth and Educational Attainment in an Indian Cohort.	2021	Yes	Yes	Yes	Yes	Yes	Yes	Yes	Yes	Yes	Unclear	Yes

Author	Title	Year	c01	c02	c03	c04	c05	c07	c09	c10	c11	c06	c08
**Park**	The impact of nutritional status and longitudinal recovery of motor and cognitive milestones in internationally adopted children.	2011	Yes	Yes	Yes	No	No	Yes	Yes	No	Unclear	Unclear	Yes
**Petersson**	Primary megalencephaly at birth and low intelligence level.	1999	Yes	Yes	Unclear	No	No	Unclear	Yes	Unclear	Yes	Unclear	Yes
**Pongcharoen**	Influence of prenatal and postnatal growth on intellectual functioning in school-aged children.	2012	Yes	Yes	Yes	Yes	Yes	Yes	Unclear	Unclear	Yes	Unclear	Yes
**Raghuram**	Head Growth Trajectory and Neurodevelopmental Outcomes in Preterm Neonates.	2017	Yes	Yes	Unclear	Unclear	Yes	Yes	Unclear	Unclear	Yes	Unclear	Yes
**Raikkonen**	Growth Trajectories and Intellectual Abilities in Young Adulthood	2009	Yes	Yes	Unclear	Unclear	Unclear	Unclear	Unclear	Unclear	Yes	Unclear	Yes
**Raikkonen**	Early life origins cognitive decline: findings in elderly men in the Helsinki Birth Cohort Study.	2013	Yes	Yes	Unclear	Yes	Yes	Yes	Yes	Unclear	Yes	Unclear	Yes
**Raz**	Physical growth in the neonatal intensive-care unit and neuropsychological performance at preschool age in very preterm-born singletons.	2015	Unclear	Yes	Unclear	Yes	Yes	Yes	No	Unclear	Yes	Unclear	Yes
**Raz**	Postnatal growth and neuropsychological performance in preterm-birth preschoolers.	2014	Unclear	Yes	Yes	Yes	Yes	Yes	No	Unclear	Yes	Unclear	Yes
**Reolon**	Influence of intrauterine and extrauterine growth on neurodevelopmental outcome of monozygotic twins.	2008	Yes	Yes	Unclear	Yes	Yes	Yes	Unclear	Unclear	Yes	Unclear	Yes

Author	Title	Year	c01	c02	c03	c04	c05	c07	c09	c10	c11	c06	c08
**Roberts**	A cumulative risk factor model for early identification of academic difficulties in premature and low birth weight infants	2007	Yes	Yes	Unclear	Yes	Yes	Yes	Yes	Unclear	Yes	Yes	Yes
**Rushton**	Cranial size and IQ in Asian Americans from birth to age seven	1997	Yes	Yes	Unclear	No	No	Yes	No	Unclear	Unclear	Unclear	Yes
**Sammallahti**	Infant growth after preterm birth and neurocognitive abilities in young adulthood.	2014	Yes	Yes	Unclear	Yes	Yes	Unclear	Yes	Unclear	Yes	Unclear	Yes
**Scharf**	Early childhood growth and cognitive outcomes: Findings from the MAL-ED study.	2018	Yes	Yes	Yes	Unclear	Unclear	Yes	Yes	Unclear	Yes	Unclear	Yes
**Selvanathan**	Head circumference, total cerebral volume and neurodevelopment in preterm neonates.	2021	Yes	Yes	Unclear	Unclear	Yes	Yes	Unclear	Unclear	Yes	Unclear	Yes
**Silva**	The relative effect of size at birth, postnatal growth and social factors on cognitive function in late childhood.	2006	Yes	Yes	Unclear	Yes	Yes	Unclear	Yes	Yes	Yes	Unclear	Yes
**Smithers**	Impact of neonatal growth on IQ and behavior at early school age.	2013	Yes	Yes	Unclear	Yes	Yes	Yes	Unclear	Yes	Yes	Unclear	Yes
**Stathis**	Head circumference in ELBW babies is associated with learning difficulties and cognition but not ADHD in the school-aged child.	1999	Yes	Yes	Unclear	Yes	Unclear	Unclear	Yes	Unclear	Yes	Unclear	Yes
**Strauss**	Growth and development of term children born with low birth weight: effects of genetic and environmental factors.	1998	Yes	Yes	Yes	No	Unclear	Yes	No	Unclear	Yes	Unclear	Yes

Author	Title	Year	c01	c02	c03	c04	c05	c07	c09	c10	c11	c06	c08
**Veena**	Association of Birthweight and Head Circumference at Birth to Cognitive Performance in 9-to 10-Year-Old Children in South India: Prospective Birth Cohort Study	2010	Yes	Yes	Yes	Yes	Yes	Yes	Yes	Yes	Yes	Unclear	Yes
**Wang**	The growth of very-low-birth-weight infants at 5 years old in Taiwan.	2014	Yes	Yes	Unclear	No	No	Yes	Unclear	No	Unclear	Unclear	Yes
**Wrigh**	Head growth and neurocognitive outcomes.	2015	Yes	Yes	Unclear	No	No	Yes	No	Unclear	Yes	Unclear	Yes
**Yajnik**	Robust determinants of neurocognitive development in children: evidence from the Pune Maternal Nutrition Study	2022	Yes	Yes	Unclear	No	No	Yes	Unclear	No	Unclear	Unclear	Yes
**Yu**	Effect of first-month head-size growth trajectory on cognitive outcomes in preterm infants.	2021	Yes	Yes	Yes	Yes	Yes	Unclear	Unclear	Unclear	Yes	Unclear	Yes
**Zhu**	Head circumference trajectories during the first two years of life and cognitive development, emotional, and behavior problems in adolescence: a cohort study	2022	Yes	Yes	Yes	Yes	Yes	Unclear	Yes	Yes	Unclear	Unclear	Yes

Checklist for Cross-Sectional Studies

Joanna Briggs Institute Critical Appraisal tools

Code	Question
t01	Were the criteria for inclusion in the sample clearly defined?
t02	Were the study subjects and the setting described in detail?
t03	Was the exposure measured in a valid and reliable way?
t04	Were objective, standard criteria used for measurement of the condition?
t05	Were confounding factors identified?
t06	Were strategies to deal with confounding factors stated?
t07	Were the outcomes measured in a valid and reliable way?
t08	Was appropriate statistical analysis used?

Author	Title	Year	t01	t02	t03	t04	t05	t06	t07	t08
Bakhiet	CORRELATIONS BETWEEN INTELLIGENCE, HEAD CIRCUMFERENCE AND HEIGHT: EVIDENCE FROM TWO SAMPLES IN SAUDI ARABIA.	2016	No	No	Unclear	Unclear	Unclear	No	Unclear	Yes
**Batterjee**	Normative data for IQ, height and head circumference for children in Saudi Arabia.	2013	Yes	Unclear	Yes	Yes	No	No	Yes	Yes
**Boynton**	Correlational analyses of the influence of basal chronological age on IQ relationships to specified anthropometric measurements.	1942	Unclear	Unclear	Unclear	Unclear	No	No	Unclear	Unclear
**Hack**	Effect of very low birth weight and subnormal head size on cognitive abilities at school age.	1991	Yes	Yes	Unclear	Unclear	Yes	Yes	Unclear	Yes
**Hein**	Physical growth and nonverbal intelligence: associations in Zambia.	2014	Unclear	Unclear	Unclear	Unclear	Unclear	Yes	Unclear	Yes

Author	Title	Year	t01	t02	t03	t04	t05	t06	t07	t08
**Huang**	Cognition and behavioural development in early childhood: the role of birth weight and postnatal growth.	2013	Yes	Yes	Yes	Yes	Yes	Yes	Yes	Yes
**Ivanovic**	A multifactorial approach of nutritional, intellectual, brain development, cardiovascular risk, socio-economic, demographic and educational variables affecting the scholastic achievement in Chilean students: An eight- year follow-up study.	2019	Yes	Yes	Yes	Yes	Unclear	Unclear	Yes	Yes
**Ivanovic**	Brain development and scholastic achievement in the Education Quality Measurement System tests in Chilean school-aged children.	2014	Yes	Unclear	Yes	Yes	No	No	Yes	Yes
**Ivanovic**	Brain development parameters and intelligence in Chilean high school graduates	2004	Yes	Unclear	Yes	Yes	No	No	Yes	Yes
**Ivanovic**	Head size and intelligence, learning, nutritional status and brain development. Head, IQ, learning, nutrition and brain.	2004	Unclear	Unclear	Yes	Yes	Unclear	Unclear	Yes	Yes
**Ivanovic**	Scholastic achievement: a multivariate analysis of nutritional, intellectual, socioeconomic, sociocultural, familial, and demographic variables in Chilean school-age children.	2004	Yes	Yes	Unclear	Yes	Unclear	Yes	Yes	Yes
**Kitchen**	Very low birth weight and growth to age 8 years. II: Head dimensions and intelligence.	1992	Unclear	Yes	Yes	Unclear	Unclear	Unclear	Unclear	Yes

Author	Title	Year	t01	t02	t03	t04	t05	t06	t07	t08
**Klein**	Is big smart?: The relation of growth to cognition.	1972	Yes	No	Unclear	Unclear	Yes	No	Unclear	Yes
**Kuban**	Developmental correlates of head circumference at birth and two years in a cohort of extremely low gestational age newborns.	2009	Yes	Yes	Yes	Yes	Unclear	Unclear	Yes	Unclear
**Lee**	The causal influence of brain size on human intelligence: Evidence from within-family phenotypic associations and GWAS modeling.	2019	Yes	Unclear	Yes	Yes	Yes	Yes	Unclear	Yes
**Lewis**	Language and motor findings in benign megalencephaly.	1989	No	No	Unclear	Unclear	No	No	Yes	Yes
**Muhoozi**	Nutritional and developmental status among 6- to 8-month-old children in southwestern Uganda: a cross-sectional study.	2016	Yes	Yes	Yes	Yes	Unclear	Unclear	Yes	Yes
**Powls**	Growth impairment in very low birthweight children at 12 years: correlation with perinatal and outcome variables.	1996	Unclear	No	Unclear	Unclear	No	Unclear	Unclear	Yes
**Rose**	Relation between physical growth and information processing in infants born in India.	1994	Yes	Yes	Yes	Yes	Unclear	No	Yes	Yes
**Sells**	Microcephaly in a normal school population.	1977	Unclear	Unclear	Yes	Yes	No	No	Unclear	Yes
**Silventoinen**	Genetic and environmental contributions to the association between anthropometric measures and iq: a study of Minnesota twins at age 11 and 17.	2012	Yes	Unclear	Yes	Yes	No	Unclear	Unclear	Yes

Author	Title	Year	t01	t02	t03	t04	t05	t06	t07	t08
**Toro Diaz**	[Anthropometric assessment and school achievement in school-age children from high school in Valparaiso, Chile].	1998	Yes	Unclear	Unclear	Unclear	No	No	Unclear	Unclear
**Weinberg**	Intelligence, reading achievement, physical size and social class. A study of St. Louis Caucasian boys aged 8-0 to 9-6 years, attending regular schools.	1974	Yes	Unclear	Yes	Yes	Unclear	No	Unclear	Yes

Checklist for Case-Control Studies
Joanna Briggs Institute Critical Appraisal tools

Code	Observation	Question
cc01		Were the groups comparable other than the presence of disease in cases or the absence of disease in controls?
cc02		Were cases and controls matched appropriately?
cc03		Were the same criteria used for identification of cases and controls?
cc04		Was exposure measured in a standard, valid and reliable way?
cc05		Was exposure measured in the same way for cases and controls?
cc06		Were confounding factors identified?
cc07		Were strategies to deal with confounding factors stated?
cc08		Were outcomes assessed in a standard, valid and reliable way for cases and controls?
cc09	Not applicable	Was the exposure period of interest long enough to be meaningful?
cc10		Was appropriate statistical analysis used?

Author	Title	Year	cc01	cc02	cc03	cc04	cc05	cc06	cc07	cc08	cc10
**Smith**	Abnormal head circumference in learning-disabled children	1981	Yes	Yes	Unclear	Unclear	Yes	No	No	Yes	Yes

Joanna Briggs Institute Critical Appraisal tools – **Total Score (Grade)**

Author	Title	Year	Study-design	Yes	Unclear	No	Grade
Alamo-Junquera	Prenatal head growth and child neuropsychological development at age 14 months	2014	Cohort	7	2	0	89%
Bach	Head circumference at birth and school performance: a nationwide cohort study of 536,921 children	2019	Cohort	7	2	0	89%
Bakhiet	CORRELATIONS BETWEEN INTELLIGENCE, HEAD CIRCUMFERENCE AND HEIGHT: EVIDENCE FROM TWO SAMPLES IN SAUDI ARABIA.	2016	Cross-sectional	1	4	3	38%
Batterjee	Normative data for IQ, height and head circumference for children in Saudi Arabia.	2013	Cross-sectional	5	1	2	69%
Beck	Prenatal and early childhood predictors of intelligence quotient (IQ) in 7-year-old Danish children from the Odense Child Cohort	2022	Cohort	8	1	0	94%
Belfort	Infant growth before and after term: effects on neurodevelopment in preterm infants.	2015	Cohort	8	1	0	94%
Bergvall	Risks for low intellectual performance related to being born small for gestational age are modified by gestational age.	2006	Cohort	6	3	0	83%

Author	Title	Year	Study-design	Yes	Unclear	No	Grade
Bergvall N	Birth characteristics and risk of low intellectual performance in early adulthood: are the associations confounded by socioeconomic factors in adolescence or familial effects?	2006	Cohort	5	4	0	78%
Boynton PL	Correlational analyses of the influence of basal chronological age on IQ relationships to specified anthropometric measurements.	1942	Cross-sectional	0	6	2	38%
Brinkis	Impact of Early Nutrient Intake and First Year Growth on Neurodevelopment of Very Low Birth Weight Newborns	2022	Cohort	5	2	2	67%
Broekman	The influence of birth size on intelligence in healthy children.	2009	Cohort	7	2	0	89%
Camargo-Figuera	Early life determinants of low IQ at age 6 in children from the 2004 Pelotas Birth Cohort: a predictive approach.	2014	Cohort	8	1	0	94%
Camp	Maternal and neonatal risk factors for mental retardation: defining the 'at-risk' child.	1998	Cohort	2	5	2	50%
Caputo	An evaluation of various parameters of maturity at birth as predictors of development at one year of life	1974	Cohort	2	5	2	50%
Charkaluk	Very preterm children free of disability or delay at age 2: predictors of schooling at age 8: a population-based longitudinal study.	2011	Cohort	6	3	0	83%

Author	Title	Year	Study-design	Yes	Unclear	No	Grade
Christian	Associations between preterm birth, small-for-gestational age, and neonatal morbidity and cognitive function among school-age children in Nepal.	2014	Cohort	5	4	0	78%
Cooke	Perinatal and postnatal factors in very preterm infants and subsequent cognitive and motor abilities.	2005	Cohort	3	6	0	67%
Cooke	Are there critical periods for brain growth in children born preterm?	2006	Cohort	2	6	1	56%
Dekhtyar	Associations of head circumference at birth with earlylife school performance and later-life occupational prestige	2015	Cohort	7	2	0	89%
Do	Poor Head Growth Is Associated with Later Mental Delay among Vietnamese Preterm Infants: A Follow-up Study.	2020	Cohort	7	2	0	89%
Dolk	The predictive value of microcephaly during the first year of life for mental retardation at seven years.	1992	Cohort	2	4	3	44%
Dupont	The Predictive Value of Head Circumference Growth during the First Year of Life on Early Child Traits.	2018	Cohort	5	4	0	78%
Eriksen	Predictors of intelligence at the age of 5: family, pregnancy and birth characteristics, postnatal influences, and postnatal growth.	2013	Cohort	7	1	1	83%

Author	Title	Year	Study-design	Yes	Unclear	No	Grade
Fattal-Valevski	Growth patterns in children with intrauterine growth retardation and their correlation to neurocognitive development.	2009	Cohort	3	2	4	44%
Ferrer	Head circumference and child ADHD symptoms and cognitive functioning: results from a large population-based cohort study	2018	Cohort	8	1	0	94%
Flensborg-Madsen	Early life predictors of intelligence in young adulthood and middle age.	2020	Cohort	6	2	1	78%
Frisk	The importance of head growth patterns in predicting the cognitive abilities and literacy skills of small-for-gestational-age children.	2015	Cohort	3	6	0	67%
Gale	The influence of head growth in fetal life, infancy, and childhood on intelligence at the ages of 4 and 8 years.	2006	Cohort	7	2	0	89%
Gale	Critical periods of brain growth and cognitive function in children.	2004	Cohort	9	0	0	100%
Gale	Foetal and postnatal head growth and risk of cognitive decline in old age.	2003	Cohort	6	2	1	78%
Gampel	Short and Long-Term Effects of Compromised Birth Weight, Head Circumference, and Apgar Scores on Neuropsychological Development.	2014	Cohort	5	4	0	78%

Author	Title	Year	Study-design	Yes	Unclear	No	Grade
Gross	Newborn head size and neurological status. Predictors of growth and development of low birth weight infants.	1978	Cohort	5	2	2	67%
Guellec	Intrauterine Growth Restriction, Head Size at Birth, and Outcome in Very Preterm Infants.	2015	Cohort	7	2	0	89%
Hack	Very low birth weight infants: effects of brain growth during infancy on intelligence quotient at 3 years of age.	1986	Cohort	4	5	0	72%
Hack	Differential effects of intrauterine and postnatal brain growth failure in infants of very low birth weight.	1989	Cohort	4	4	1	67%
Hack	Effect of very low birth weight and subnormal head size on cognitive abilities at school age.	1991	Cross-sectional	5	3	0	81%
Han	Functional principal component analysis for identifying multivariate patterns and archetypes of growth, and their association with long-term cognitive development.	2018	Cohort	3	5	1	61%
Hein	Physical growth and nonverbal intelligence: associations in Zambia.	2014	Cross-sectional	2	6	0	63%
Heinonen	Prenatal and postnatal growth and cognitive abilities at 56 months of age: A longitudinal study of infants born at term	2008	Cohort	7	2	0	89%

Author	Title	Year	Study-design	Yes	Unclear	No	Grade
Hickey	Extreme prematurity, growth and neurodevelopment at 8 years: a cohort study.	2021	Cohort	4	5	0	72%
Huang	Cognition and behavioural development in early childhood: the role of birth weight and postnatal growth.	2013	Cross-sectional	8	0	0	100%
Ivanovic	Twelve-year follow-up study of the impact of nutritional status at the onset of elementary school on later educational situation of Chilean school-age children.	2008	Cohort	6	3	0	83%
Ivanovic	A multifactorial approach of nutritional, intellectual, brain development, cardiovascular risk, socio-economic, demographic and educational variables affecting the scholastic achievement in Chilean students: An eight- year follow-up study.	2019	Cross-sectional	6	2	0	88%
Ivanovic	Brain development and scholastic achievement in the Education Quality Measurement System tests in Chilean school-aged children.	2014	Cross-sectional	5	1	2	69%
Ivanovic	Brain development parameters and intelligence in Chilean high school graduates	2004	Cross-sectional	5	1	2	69%

Author	Title	Year	Study-design	Yes	Unclear	No	Grade
Ivanovic	Head size and intelligence, learning, nutritional status and brain development. Head, IQ, learning, nutrition and brain.	2004	Cross-sectional	4	4	0	75%
Ivanovic	Impact of anthropometric nutritional parameters on the university selection test in Chile: A multifactorial approach.	2019	Cohort	6	2	1	78%
Ivanovic	Impact of nutritional status at the onset of elementary school on academic aptitude test achievement at the end of high school in a multicausal approach.	2009	Cohort	5	4	0	78%
Ivanovic	Neuropsychological Parameters Affecting the Academic Aptitude Test (AAT) Achievement at the End of High School in 1996 and Their Impact on Job Status in 2002: A Multifactorial Approach in a Follow-up Study.	2006	Cohort	2	3	4	39%
Ivanovic	Scholastic achievement: a multivariate analysis of nutritional, intellectual, socioeconomic, sociocultural, familial, and demographic variables in Chilean school-age children.	2004	Cross-sectional	6	2	0	88%
Jaekel	Head Growth and Intelligence from Birth to Adulthood in Very Preterm and Term Born Individuals.	2018	Cohort	7	2	0	89%

Author	Title	Year	Study-design	Yes	Unclear	No	Grade
Jensen	Cognitive ability in adolescents born small for gestational age: Associations with fetal growth velocity, head circumference and postnatal growth.	2015	Cohort	6	2	1	78%
Kan	The association of growth impairment with neurodevelopmental outcome at eight years of age in very preterm children.	2008	Cohort	7	2	0	89%
Kim	Cognitive Outcomes of Children with Very Low Birth Weight at 3 to 5 Years of Age.	2020	Cohort	4	5	0	72%
Kirkegaard	Associations of birth size, infancy, and childhood growth with intelligence quotient at 5 years of age: a Danish cohort study.	2020	Cohort	8	1	0	94%
Kitchen	Very low birth weight and growth to age 8 years. II: Head dimensions and intelligence.	1992	Cross-sectional	3	5	0	69%
Klein	Is big smart?: The relation of growth to cognition.	1972	Cross-sectional	3	3	2	56%
Koller	Patterns of cognitive development in very low birth weight children during the first six years of life.	1997	Cohort	3	3	3	50%
Koshy	Association between head circumference at two years and second and fifth year cognition.	2021	Cohort	8	1	0	94%

Author	Title	Year	Study-design	Yes	Unclear	No	Grade
Kroupina	Associations between physical growth and general cognitive functioning in international adoptees from Eastern Europe at 30 months post-arrival	2015	Cohort	7	2	0	89%
Kuban	Developmental correlates of head circumference at birth and two years in a cohort of extremely low gestational age newborns.	2009	Cross-sectional	5	3	0	81%
Larroque	School difficulties in 20-year-olds who were born small for gestational age at term in a regional cohort study.	2001	Cohort	7	1	1	83%
Lee	The causal influence of brain size on human intelligence: Evidence from within-family phenotypic associations and GWAS modeling.	2019	Cross-sectional	6	2	0	88%
Lei	Choosing the Best Newborn Anthropometric Measure Associated With the Risks and Outcomes of Intrauterine Growth Restriction	2015	Cohort	7	2	0	89%
Leppänen	Antenatal and postnatal growth and 5-year cognitive outcome in very preterm infants.	2014	Cohort	7	2	0	89%
Lewis	Language and motor findings in benign megalencephaly.	1989	Cross-sectional	2	2	4	38%
Li	Relative importance of birth size and postnatal growth for women's educational achievement.	2004	Cohort	8	1	0	94%

Author	Title	Year	Study-design	Yes	Unclear	No	Grade
Lidzba	Growth in very preterm children: Head growth after discharge is the best independent predictor for cognitive outcome.	2016	Cohort	6	3	0	83%
Lira	Early head growth: relation with IQ at 8 years and determinants in term infants of low and appropriate birthweight.	2009	Cohort	8	1	0	94%
Lorenz	Indices of body and brain size at birth and at the age of 2 years: relations to cognitive outcome at the age of 16 years in low birth weight infants.	2009	Cohort	6	3	0	83%
Lundgren	Intellectual and psychological performance in males born small for gestational age with and without catch-up growth.	2001	Cohort	4	5	0	72%
Lundgren	Short Adult Stature and Overweight Are Associated with Poor Intellectual Performance in Subjects Born Preterm	2011	Cohort	5	4	0	78%
Lundgren	Birth characteristics and different dimensions of intellectual performance in young males: a nationwide population-based study.	2003	Cohort	4	5	0	72%

Author	Title	Year	Study-design	Yes	Unclear	No	Grade
Malacova	Neighbourhood socioeconomic status and maternal factors at birth as moderators of the association between birth characteristics and school attainment: a population study of children attending government schools in Western Australia.	2009	Cohort	6	3	0	83%
Mccall	Developmental changes in head-circumference and mental-performance growth rates: a test of Epstein's phrenoblysis hypothesis.	1983	Cohort	6	1	2	72%
Miller	Outcomes of children adopted from Eastern Europe.	2009	Cohort	4	3	2	61%
Muhoozi	Nutritional and developmental status among 6- to 8-month-old children in southwestern Uganda: a cross-sectional study.	2016	Cross-sectional	6	2	0	88%
Nash	Pattern of growth of very low birth weight preterm infants, assessed using the WHO Growth Standards, is associated with neurodevelopment.	2011	Cohort	6	3	0	83%
Nelson	Head size at one year as a predictor of four-year IQ	1970	Cohort	3	4	2	56%
Neubauer	Poor postdischarge head growth is related to a 10% lower intelligence quotient in very preterm infants at the chronological age of five years.	2016	Cohort	4	4	1	67%

Author	Title	Year	Study-design	Yes	Unclear	No	Grade
Nicolaou	Factors associated with head circumference and indices of cognitive development in early childhood	2020	Cohort	7	2	0	89%
Ochiai	Head circumference and long-term outcome in small-for-gestational age infants.	2008	Cohort	3	3	3	50%
Pandey	Childhood Head Growth and Educational Attainment in an Indian Cohort.	2021	Cohort	9	0	0	100%
Park	The impact of nutritional status and longitudinal recovery of motor and cognitive milestones in internationally adopted children.	2011	Cohort	5	1	3	61%
Petersson	Primary megalencephaly at birth and low intelligence level.	1999	Cohort	4	3	2	61%
Pongcharoen	Influence of prenatal and postnatal growth on intellectual functioning in school-aged children.	2012	Cohort	7	2	0	89%
Powls	Growth impairment in very low birthweight children at 12 years: correlation with perinatal and outcome variables.	1996	Cross-sectional	1	5	2	44%
Raghuram	Head Growth Trajectory and Neurodevelopmental Outcomes in Preterm Neonates.	2017	Cohort	5	4	0	78%
Raikkonen	Growth Trajectories and Intellectual Abilities in Young Adulthood	2009	Cohort	3	6	0	67%

Author	Title	Year	Study-design	Yes	Unclear	No	Grade
Raikkonen	Early life origins cognitive decline: findings in elderly men in the Helsinki Birth Cohort Study.	2013	Cohort	7	2	0	89%
Raz	Physical growth in the neonatal intensive-care unit and neuropsychological performance at preschool age in very preterm-born singletons.	2015	Cohort	5	3	1	72%
Raz	Postnatal growth and neuropsychological performance in preterm-birth preschoolers.	2014	Cohort	6	2	1	78%
Reolon	Influence of intrauterine and extrauterine growth on neurodevelopmental outcome of monozygotic twins.	2008	Cohort	6	3	0	83%
Roberts	A cumulative risk factor model for early identification of academic difficulties in premature and low birth weight infants	2007	Cohort	7	2	0	89%
Rose	Relation between physical growth and information processing in infants born in India.	1994	Cross-sectional	6	1	1	81%
Rushton	Cranial size and IQ in Asian Americans from birth to age seven	1997	Cohort	3	3	3	50%
Sammallahti	Infant growth after preterm birth and neurocognitive abilities in young adulthood.	2014	Cohort	6	3	0	83%

Author	Title	Year	Study-design	Yes	Unclear	No	Grade
Sandstead	Nutritional deficiencies in disadvantaged preschool children. Their relationship to mental development.	1971	Lack of information	-	-	-	-
Scharf	Early childhood growth and cognitive outcomes: Findings from the MAL-ED study.	2018	Cohort	6	3	0	83%
Sells	Microcephaly in a normal school population.	1977	Cross-sectional	3	3	2	56%
Selvanathan	Head circumference, total cerebral volume and neurodevelopment in preterm neonates.	2021	Cohort	5	4	0	78%
Silva	The relative effect of size at birth, postnatal growth and social factors on cognitive function in late childhood.	2006	Cohort	7	2	0	89%
Silventoinen	Genetic and environmental contributions to the association between anthropometric measures and iq: a study of Minnesota twins at age 11 and 17.	2012	Cross-sectional	4	3	1	69%
Smith	Abnormal head circumference in learning-disabled children	1981	Case-control	5	2	2	67%
Smithers	Impact of neonatal growth on IQ and behavior at early school age.	2013	Cohort	7	2	0	89%

Author	Title	Year	Study-design	Yes	Unclear	No	Grade
Stathis	Head circumference in ELBW babies is associated with learning difficulties and cognition but not ADHD in the school-aged child.	1999	Cohort	5	4	0	78%
Strauss	Growth and development of term children born with low birth weight: effects of genetic and environmental factors.	1998	Cohort	5	2	2	67%
Toro Diaz	[Anthropometric assessment and school achievement in school-age children from high school in Valparaiso, Chile].	1998	Cross-sectional	1	5	2	44%
Veena	Association of Birthweight and Head Circumference at Birth to Cognitive Performance in 9-to 10-Year-Old Children in South India: Prospective Birth Cohort Study	2010	Cohort	9	0	0	100%
Wang	The growth of very-low-birth-weight infants at 5 years old in Taiwan.	2014	Cohort	3	3	3	50%
Weinberg	Intelligence, reading achievement, physical size and social class. A study of St. Louis Caucasian boys aged 8-0 to 9-6 years, attending regular schools.	1974	Cross-sectional	4	3	1	69%
Wrigh	Head growth and neurocognitive outcomes.	2015	Cohort	4	2	3	56%

Author	Title	Year	Study-design	Yes	Unclear	No	Grade
Yajnik	Robust determinants of neurocognitive development in children: evidence from the Pune Maternal Nutrition Study	2022	Cohort	3	3	3	50%
Yu	Effect of first-month head-size growth trajectory on cognitive outcomes in preterm infants.	2021	Cohort	6	3	0	83%
Zhu	Head circumference trajectories during the first two years of life and cognitive development, emotional, and behavior problems in adolescence: a cohort study	2022	Cohort	7	2	0	89%
