# Supplementary material for: Head circumference and intelligence, schooling, employment, and income: a systematic review
Source: BMC Pediatr. 2024 Nov 7;24:709. doi: 10.1186/s12887-024-05159-2 (PMC11542250; doi:10.1186/s12887-024-05159-2)
Supplement: Supplementary file 3 — Additional file 3. Minimum quality criteria used for restricted analysis. The file shows the minimum quality criteria used for restricted analysis applied in all included articles. [file 12887_2024_5159_MOESM3_ESM.pdf]

Author, year	***Title***	***Was the sample representative of a well-defined population group?***	***Were socioeconomic level and maternal IQ or maternal schooling included in the adjusted analysis?***	***Were well-defined mediators (i.e., schooling, intelligence, or subsequent head circumference measured between exposure and outcome of interest) included as confounders in the adjusted analysis?***	***Were the measurements of the head circumference standardized for age and sex or were these variables included in the adjusted analysis?***	***Was a measure of association between head circumference and the outcome of interest calculated?***	***Was this article selected for the restricted analysis?***
Alamo Junquera, 2014	Prenatal head growth and child neuropsychological development at age 14 months	Yes	Yes	No	Yes	Yes	Yes
Bach, 2020	Head circumference at birth and school performance: a nationwide cohort study of 536,921 children	Yes	Yes	No	Yes	Yes	Yes
Bakhiet, 2017	Correlations between intelligence, head circumference and height: evidence from two samples in Saudi Arabia.	Yes	No	No	Yes	No	No
Batterjee, 2013	Normative data for IQ, height and head circumference for children in Saudi Arabia.	Yes	No	No	Yes	No	No
Beck, 2022	Prenatal and early childhood predictors of intelligence quotient (IQ) in 7-year-old Danish children from the Odense Child Cohort	Yes	No	Yes	Yes	Yes	No
Belfort, 2011	Infant growth before and after term: effects on neurodevelopment in preterm infants.	Yes	No	No	Yes	Yes	No
Bergvall, 2006	Birth characteristics and risk of low intellectual performance in early adulthood: are the associations confounded	Yes	Yes	No	Yes	Yes	Yes

Author, year	*Title*	*Was the sample representative of a well-defined population group?*	*Were socioeconomic level and maternal IQ or maternal schooling included in the adjusted analysis?*	*Were well-defined mediators (i.e., schooling, intelligence, or subsequent head circumference measured between exposure and outcome of interest) included as confounders in the adjusted analysis?*	*Were the measurements of the head circumference standardized for age and sex or were these variables included in the adjusted analysis?*	*Was a measure of association between head circumference and the outcome of interest calculated?*	*Was this article selected for the restricted analysis?*
	by socioeconomic factors in adolescence or familial effects?						
Bergvall, 2006	Risks for low intellectual performance related to being born small for gestational age are modified by gestational age.	Yes	Yes	No	Yes	Yes	Yes
Boynton, 1942	Correlational analyses of the influence of basal chronological age on IQ relationships to specified anthropometric measurements.	Yes	No	No	Yes	No	No
Brinkis, 2022	Impact of Early Nutrient Intake and First Year Growth on Neurodevelopment of Very Low Birth Weight Newborns	Yes	No	No	Yes	Yes	No
Broekman, 2009	The influence of birth size on intelligence in healthy children.	Yes	No	No	Yes	Yes	No
Camargo-Figuera, 2014	Early life determinants of low IQ at age 6 in children from the 2004 Pelotas Birth Cohort: a predictive approach.	Yes	Yes	No	Yes	Yes	Yes
Camp, 1998	Maternal and neonatal risk factors for mental retardation: defining the 'at-risk' child.	Yes	No	No	No	Yes	No

Author, year	*Title*	*Was the sample representative of a well-defined population group?*	*Were socioeconomic level and maternal IQ or maternal schooling included in the adjusted analysis?*	*Were well-defined mediators (i.e., schooling, intelligence, or subsequent head circumference measured between exposure and outcome of interest) included as confounders in the adjusted analysis?*	*Were the measurements of the head circumference standardized for age and sex or were these variables included in the adjusted analysis?*	*Was a measure of association between head circumference and the outcome of interest calculated?*	*Was this article selected for the restricted analysis?*
Caputo, 1974	An evaluation of various parameters of maturity at birth as predictors of development at one year of life	No	Yes	Yes	Yes	No	No
Charkaluk, 2011	Very preterm children free of disability or delay at age 2: predictors of schooling at age 8: a population-based longitudinal study.	Yes	No	Yes	Yes	Yes	No
Christian, 2014	Associations between preterm birth, small-for-gestational age, and neonatal morbidity and cognitive function among school-age children in Nepal.	Yes	Yes	Yes	Yes	Yes	No
Cooke, 2005	Perinatal and postnatal factors in very preterm infants and subsequent cognitive and motor abilities.	Yes	No	No	Yes	Yes	No
Cooke, 2006	Are there critical periods for brain growth in children born preterm?	Yes	No	No	Yes	No	No
Dekhtyar, 2015	Associations of head circumference at birth with earlylife school performance and later-life occupational prestige	Yes	No	No	Yes	Yes	No
Do, 2021	Poor Head Growth Is Associated with Later	Yes	No	No	Yes	Yes	No

Author, year	*Title*	*Was the sample representative of a well-defined population group?*	*Were socioeconomic level and maternal IQ or maternal schooling included in the adjusted analysis?*	*Were well-defined mediators (i.e., schooling, intelligence, or subsequent head circumference measured between exposure and outcome of interest) included as confounders in the adjusted analysis?*	*Were the measurements of the head circumference standardized for age and sex or were these variables included in the adjusted analysis?*	*Was a measure of association between head circumference and the outcome of interest calculated?*	*Was this article selected for the restricted analysis?*
	Mental Delay among Vietnamese Preterm Infants: A Follow-up Study.						
Dolk, 1991	The predictive value of microcephaly during the first year of life for mental retardation at seven years.	Yes	No	No	Yes	No	No
Dupont, 2018	The Predictive Value of Head Circumference Growth during the First Year of Life on Early Child Traits.	Yes	Yes	No	Yes	Yes	Yes
Eriksen, 2013	Predictors of intelligence at the age of 5: family, pregnancy and birth characteristics, postnatal influences, and postnatal growth.	Yes	No	Yes	Yes	Yes	No
Fattal-Valevski, 2009	Growth patterns in children with intrauterine growth retardation and their correlation to neurocognitive development.	Yes	No	No	No	No	No
Ferrer, 2019	Head circumference and child ADHD symptoms and cognitive functioning: results from a large population-based cohort study	Yes	Yes	No	Yes	Yes	Yes

Author, year	*Title*	***Was the sample representative of a well-defined population group?***	***Were socioeconomic level and maternal IQ or maternal schooling included in the adjusted analysis?***	***Were well-defined mediators (i.e., schooling, intelligence, or subsequent head circumference measured between exposure and outcome of interest) included as confounders in the adjusted analysis?***	***Were the measurements of the head circumference standardized for age and sex or were these variables included in the adjusted analysis?***	***Was a measure of association between head circumference and the outcome of interest calculated?***	***Was this article selected for the restricted analysis?***
Flensborg-Madsen, 2020	Early life predictors of intelligence in young adulthood and middle age.	Yes	No	Yes	Yes	Yes	No
Frisk, 2002	The importance of head growth patterns in predicting the cognitive abilities and literacy skills of small-for-gestational-age children.	Yes	No	No	No	No	No
Gale, 2003	Foetal and postnatal head growth and risk of cognitive decline in old age.	No	No	Yes	Yes	No	No
Gale, 2004	Critical periods of brain growth and cognitive function in children.	Yes	Yes	No	Yes	Yes	Yes
Gale, 2006	The influence of head growth in fetal life, infancy, and childhood on intelligence at the ages of 4 and 8 years	Yes	Yes	No	Yes	Yes	Yes
Gampel, 2014	Short and Long-Term Effects of Compromised Birth Weight, Head Circumference, and Apgar Scores on Neuropsychological Development.	Yes	No	No	Yes	Yes	No
Gross, 1978	Newborn head size and neurological status. Predictors of growth and	Yes	No	No	No	No	No

Author, year	*Title*	***Was the sample representative of a well-defined population group?***	***Were socioeconomic level and maternal IQ or maternal schooling included in the adjusted analysis?***	***Were well-defined mediators (i.e., schooling, intelligence, or subsequent head circumference measured between exposure and outcome of interest) included as confounders in the adjusted analysis?***	***Were the measurements of the head circumference standardized for age and sex or were these variables included in the adjusted analysis?***	***Was a measure of association between head circumference and the outcome of interest calculated?***	***Was this article selected for the restricted analysis?***
	development of low birth weight infants.						
Guellec, 2015	Intrauterine Growth Restriction, Head Size at Birth, and Outcome in Very Preterm Infants.	Yes	No	No	Yes	Yes	No
Hack, 1986	Very low birth weight infants: effects of brain growth during infancy on intelligence quotient at 3 years of age.	Yes	No	No	No	Yes	No
Hack, 1989	Differential effects of intrauterine and postnatal brain growth failure in infants of very low birth weight.	Yes	No	No	Yes	Yes	No
Hack, 1991	Effect of very low birth weight and subnormal head size on cognitive abilities at school age.	Yes	No	No	No	Yes	No
Han, 2018	Functional principal component analysis for identifying multivariate patterns and archetypes of growth, and their association with long-term cognitive development.	Yes	No	No	Yes	Yes	No
Hein, 2014	Physical growth and nonverbal intelligence: associations in Zambia.	Yes	No	Yes	No	Yes	No
Heinonen, 2008	Prenatal and postnatal growth and cognitive	Yes	No	No	Yes	Yes	No

Author, year	*Title*	*Was the sample representative of a well-defined population group?*	*Were socioeconomic level and maternal IQ or maternal schooling included in the adjusted analysis?*	*Were well-defined mediators (i.e., schooling, intelligence, or subsequent head circumference measured between exposure and outcome of interest) included as confounders in the adjusted analysis?*	*Were the measurements of the head circumference standardized for age and sex or were these variables included in the adjusted analysis?*	*Was a measure of association between head circumference and the outcome of interest calculated?*	*Was this article selected for the restricted analysis?*
	abilities at 56 months of age: A longitudinal study of infants born at term						
Hickey, 2021	Extreme prematurity, growth and neurodevelopment at 8 years: a cohort study.	Yes	Yes	No	Yes	Yes	Yes
Huang, 2013	Cognition and behavioural development in early childhood: the role of birth weight and postnatal growth.	Yes	Yes	No	No	Yes	No
Ivanovic, 2004	Head size and intelligence, learning, nutritional status and brain development. Head, IQ, learning, nutrition and brain.	No	No	No	Yes	No	No
Ivanovic, 2004	Brain development parameters and intelligence in Chilean high school graduates	No	No	No	No	No	No
Ivanovic, 2004	Scholastic achievement: a multivariate analysis of nutritional, intellectual, socioeconomic, sociocultural, familial, and demographic variables in Chilean school-age children.	Yes	Yes	Yes	No	Yes	No
Ivanovic, 2006	Neuropsychological Parameters Affecting the Academic Aptitude Test	No	No	No	Yes	No	No

Author, year	*Title*	*Was the sample representative of a well-defined population group?*	*Were socioeconomic level and maternal IQ or maternal schooling included in the adjusted analysis?*	*Were well-defined mediators (i.e., schooling, intelligence, or subsequent head circumference measured between exposure and outcome of interest) included as confounders in the adjusted analysis?*	*Were the measurements of the head circumference standardized for age and sex or were these variables included in the adjusted analysis?*	*Was a measure of association between head circumference and the outcome of interest calculated?*	*Was this article selected for the restricted analysis?*
	(AAT) Achievement at the End of High School in 1996 and Their Impact on Job Status in 2002: A Multifactorial Approach in a Follow-up Study.						
Ivanovic, 2008	Twelve-year follow-up study of the impact of nutritional status at the onset of elementary school on later educational situation of Chilean school-age children.	Yes	No	Yes	Yes	Yes	No
Ivanovic, 2009	Impact of nutritional status at the onset of elementary school on academic aptitude test achievement at the end of high school in a multicausal approach.	Yes	No	Yes	No	Yes	No
Ivanovic, 2014	Brain development and scholastic achievement in the Education Quality Measurement System tests in Chilean school-aged children.	Yes	No	No	Yes	Yes	No
Ivanovic, 2019	A multifactorial approach of nutritional, intellectual, brain development, cardiovascular risk, socio-economic, demographic and educational variables	Yes	No	Yes	Yes	Yes	No

Author, year	*Title*	*Was the sample representative of a well-defined population group?*	*Were socioeconomic level and maternal IQ or maternal schooling included in the adjusted analysis?*	*Were well-defined mediators (i.e., schooling, intelligence, or subsequent head circumference measured between exposure and outcome of interest) included as confounders in the adjusted analysis?*	*Were the measurements of the head circumference standardized for age and sex or were these variables included in the adjusted analysis?*	*Was a measure of association between head circumference and the outcome of interest calculated?*	*Was this article selected for the restricted analysis?*
	affecting the scholastic achievement in Chilean students: An eight- year follow-up study.						
Ivanovic, 2019	Impact of anthropometric nutritional parameters on the university selection test in Chile: A multifactorial approach.	Yes	No	Yes	Yes	Yes	No
Jaekel, 2019	Head Growth and Intelligence from Birth to Adulthood in Very Preterm and Term Born Individuals.	No	Yes	No	Yes	Yes	No
Jensen, 2015	Cognitive ability in adolescents born small for gestational age: Associations with fetal growth velocity, head circumference and postnatal growth.	Yes	No	No	Yes	Yes	No
Kan, 2008	The association of growth impairment with neurodevelopmental outcome at eight years of age in very preterm children.	Yes	Yes	No	Yes	Yes	Yes
Kim, 2020	Cognitive Outcomes of Children with Very Low Birth Weight at 3 to 5 Years of Age.	Yes	No	No	No	No	No
Kinkegaard, 2020	Associations of birth size, infancy, and childhood	Yes	Yes	No	Yes	Yes	Yes

Author, year	*Title*	*Was the sample representative of a well-defined population group?*	*Were socioeconomic level and maternal IQ or maternal schooling included in the adjusted analysis?*	*Were well-defined mediators (i.e., schooling, intelligence, or subsequent head circumference measured between exposure and outcome of interest) included as confounders in the adjusted analysis?*	*Were the measurements of the head circumference standardized for age and sex or were these variables included in the adjusted analysis?*	*Was a measure of association between head circumference and the outcome of interest calculated?*	*Was this article selected for the restricted analysis?*
	growth with intelligence quotient at 5 years of age: a Danish cohort study.						
Kitchen, 1992	Very low birth weight and growth to age 8 years. II: Head dimensions and intelligence.	No	Yes	No	Yes	Yes	No
Klein, 1972	Is big smart?: The relation of growth to cognition.	Yes	No	No	No	Yes	No
Koller, 1997	Patterns of cognitive development in very low birth weight children during the first six years of life.	Yes	No	No	No	No	No
Koshy, 2021	Association between head circumference at two years and second and fifth year cognition.	Yes	Yes	No	Yes	Yes	Yes
Kroupina, 2015	Associations between physical growth and general cognitive functioning in international adoptees from Eastern Europe at 30 months post-arrival	Yes	No	Yes	Yes	Yes	No
Kuban, 2009	Developmental correlates of head circumference at birth and two years in a cohort of extremely low gestational age newborns.	Yes	No	No	Yes	Yes	No

Author, year	*Title*	*Was the sample representative of a well-defined population group?*	*Were socioeconomic level and maternal IQ or maternal schooling included in the adjusted analysis?*	*Were well-defined mediators (i.e., schooling, intelligence, or subsequent head circumference measured between exposure and outcome of interest) included as confounders in the adjusted analysis?*	*Were the measurements of the head circumference standardized for age and sex or were these variables included in the adjusted analysis?*	*Was a measure of association between head circumference and the outcome of interest calculated?*	*Was this article selected for the restricted analysis?*
Larroque, 2001	School difficulties in 20-year-olds who were born small for gestational age at term in a regional cohort study.	Yes	Yes	No	Yes	Yes	Yes
Lee, 2019	The causal influence of brain size on human intelligence: Evidence from within-family phenotypic associations and GWAS modeling.	Yes	Yes	No	Yes	Yes	Yes
Lei, 2015	Choosing the Best Newborn Anthropometric Measure Associated With the Risks and Outcomes of Intrauterine Growth Restriction	Yes	Yes	No	Yes	Yes	Yes
Leppanen, 2014	Antenatal and postnatal growth and 5-year cognitive outcome in very preterm infants.	Yes	No	No	Yes	Yes	No
Lewis, 1989	Language and motor findings in benign megalencephaly.	Yes	No	No	Yes	No	No
Li, 2004	Relative importance of birth size and postnatal growth for women's educational achievement.	Yes	Yes	Yes	Yes	Yes	No
Lidzba, 2016	Growth in very preterm children: Head growth after discharge is the best independent predictor for cognitive outcome.	Yes	No	No	No	No	No

Author, year	*Title*	***Was the sample representative of a well-defined population group?***	***Were socioeconomic level and maternal IQ or maternal schooling included in the adjusted analysis?***	***Were well-defined mediators (i.e., schooling, intelligence, or subsequent head circumference measured between exposure and outcome of interest) included as confounders in the adjusted analysis?***	***Were the measurements of the head circumference standardized for age and sex or were these variables included in the adjusted analysis?***	***Was a measure of association between head circumference and the outcome of interest calculated?***	***Was this article selected for the restricted analysis?***
Lira, 2010	Early head growth: relation with IQ at 8 years and determinants in term infants of low and appropriate birthweight.	Yes	Yes	Yes	Yes	Yes	No
Lorenz, 2009	Indices of body and brain size at birth and at the age of 2 years: relations to cognitive outcome at the age of 16 years in low birth weight infants.	Yes	No	No	No	Yes	No
Lundgren, 2001	Intellectual and psychological performance in males born small for gestational age with and without catch-up growth.	Yes	No	No	Yes	Yes	No
Lundgren, 2003	Birth characteristics and different dimensions of intellectual performance in young males: a nationwide population-based study.	Yes	No	No	Yes	Yes	No
Lundgren, 2011	Short Adult Stature and Overweight Are Associated with Poor Intellectual Performance in Subjects Born Preterm	Yes	No	Yes	No	Yes	No
Malacova, 2009	Neighbourhood socioeconomic status and maternal factors at birth as moderators of the association between birth	Yes	Yes	No	Yes	Yes	Yes

Author, year	*Title*	*Was the sample representative of a well-defined population group?*	*Were socioeconomic level and maternal IQ or maternal schooling included in the adjusted analysis?*	*Were well-defined mediators (i.e., schooling, intelligence, or subsequent head circumference measured between exposure and outcome of interest) included as confounders in the adjusted analysis?*	*Were the measurements of the head circumference standardized for age and sex or were these variables included in the adjusted analysis?*	*Was a measure of association between head circumference and the outcome of interest calculated?*	*Was this article selected for the restricted analysis?*
	characteristics and school attainment: a population study of children attending government schools in Western Australia.						
McCall, 1983	Developmental changes in head-circumference and mental-performance growth rates: a test of Epstein's phrenoblysis hypothesis.	Yes	No	No	No	No	No
Miller, 2009	Outcomes of children adopted from Eastern Europe.	Yes	No	No	No	No	No
Muhoozi, 2016	Nutritional and developmental status among 6- to 8-month-old children in southwestern Uganda: a cross-sectional study.	Yes	No	No	No	No	No
Nash, 2011	Pattern of growth of very low birth weight preterm infants, assessed using the WHO Growth Standards, is associated with neurodevelopment.	Yes	No	No	Yes	No	No
Nelson, 1970	Head size at one year as a predictor of four-year IQ	Yes	No	No	No	No	No
Neubauer, 2016	Poor postdischarge head growth is related to a 10% lower intelligence quotient in very preterm	Yes	No	No	No	No	No

Author, year	*Title*	*Was the sample representative of a well-defined population group?*	*Were socioeconomic level and maternal IQ or maternal schooling included in the adjusted analysis?*	*Were well-defined mediators (i.e., schooling, intelligence, or subsequent head circumference measured between exposure and outcome of interest) included as confounders in the adjusted analysis?*	*Were the measurements of the head circumference standardized for age and sex or were these variables included in the adjusted analysis?*	*Was a measure of association between head circumference and the outcome of interest calculated?*	*Was this article selected for the restricted analysis?*
	infants at the chronological age of five years.						
Nicolaou, 2020	Factors associated with head circumference and indices of cognitive development in early childhood	Yes	No	No	No	Yes	No
Ochiai, 2008	Head circumference and long-term outcome in small-for-gestational age infants.	Yes	No	No	No	No	No
Pandey, 2021	Childhood Head Growth and Educational Attainment in an Indian Cohort.	Yes	No	No	Yes	Yes	No
Park, 2011	The impact of nutritional status and longitudinal recovery of motor and cognitive milestones in internationally adopted children.	Yes	No	No	No	Yes	No
Petersson, 1999	Primary megalencephaly at birth and low intelligence level.	Yes	No	No	No	Yes	No
Pongcharoen, 2012	Influence of prenatal and postnatal growth on intellectual functioning in school-aged children.	Yes	Yes	No	Yes	Yes	Yes
Powls, 1996	Growth impairment in very low birthweight children at 12 years:	Yes	No	No	No	Yes	No

Author, year	*Title*	***Was the sample representative of a well-defined population group?***	***Were socioeconomic level and maternal IQ or maternal schooling included in the adjusted analysis?***	***Were well-defined mediators (i.e., schooling, intelligence, or subsequent head circumference measured between exposure and outcome of interest) included as confounders in the adjusted analysis?***	***Were the measurements of the head circumference standardized for age and sex or were these variables included in the adjusted analysis?***	***Was a measure of association between head circumference and the outcome of interest calculated?***	***Was this article selected for the restricted analysis?***
	correlation with perinatal and outcome variables.						
Raghuram, 2017	Head Growth Trajectory and Neurodevelopmental Outcomes in Preterm Neonates.	Yes	No	No	Yes	Yes	No
Räikkönen, 2009	Growth Trajectories and Intellectual Abilities in Young Adulthood: The Helsinki Birth Cohort study.	Yes	No	No	No	Yes	No
Raikkonen, 2013	Early life origins cognitive decline: findings in elderly men in the Helsinki Birth Cohort Study.	Yes	Yes	Yes	Yes	Yes	No
Raz, 2014	Postnatal growth and neuropsychological performance in preterm-birth preschoolers.	Yes	No	No	Yes	Yes	No
Raz, 2015	Physical growth in the neonatal intensive-care unit and neuropsychological performance at preschool age in very preterm-born singletons.	Yes	No	No	Yes	Yes	No
Reolon, 2008	Influence of intrauterine and extrauterine growth on neurodevelopmental outcome of monozygotic twins.	Yes	Yes	No	Yes	Yes	Yes

Author, year	*Title*	***Was the sample representative of a well-defined population group?***	***Were socioeconomic level and maternal IQ or maternal schooling included in the adjusted analysis?***	***Were well-defined mediators (i.e., schooling, intelligence, or subsequent head circumference measured between exposure and outcome of interest) included as confounders in the adjusted analysis?***	***Were the measurements of the head circumference standardized for age and sex or were these variables included in the adjusted analysis?***	***Was a measure of association between head circumference and the outcome of interest calculated?***	***Was this article selected for the restricted analysis?***
Roberts, 2007	A cumulative risk factor model for early identification of academic difficulties in premature and low birth weight infants	Yes	Yes	No	No	Yes	No
Rose, 1994	Relation between physical growth and information processing in infants born in India.	Yes	No	No	No	No	No
Rushton, 1997	Cranial size and IQ in Asian Americans from birth to age seven	Yes	No	No	No	No	No
Sammallahti, 2014	Infant growth after preterm birth and neurocognitive abilities in young adulthood.	Yes	No	No	Yes	Yes	No
Sandstead, 1971	Nutritional deficiencies in disadvantaged preschool children. Their relationship to mental development.	Yes	No	No	No	No	No
Scharf, 2018	Early childhood growth and cognitive outcomes: Findings from the MAL-ED study.	Yes	No	No	No	Yes	No
Sells, 1977	Microcephaly in a normal school population.	Yes	No	No	Yes	No	No
Selvanathan, 2021	Head circumference, total cerebral volume and neurodevelopment in preterm neonates.	Yes	No	No	Yes	Yes	No

Author, year	***Title***	***Was the sample representative of a well-defined population group?***	***Were socioeconomic level and maternal IQ or maternal schooling included in the adjusted analysis?***	***Were well-defined mediators (i.e., schooling, intelligence, or subsequent head circumference measured between exposure and outcome of interest) included as confounders in the adjusted analysis?***	***Were the measurements of the head circumference standardized for age and sex or were these variables included in the adjusted analysis?***	***Was a measure of association between head circumference and the outcome of interest calculated?***	***Was this article selected for the restricted analysis?***
Silva, 2006	The relative effect of size at birth, postnatal growth and social factors on cognitive function in late childhood.	Yes	No	Yes	Yes	Yes	No
Silventoinen, 2012	Genetic and environmental contributions to the association between anthropometric measures and iq: a study of Minnesota twins at age 11 and 17.	Yes	Yes	No	Yes	No	No
Smith, 1981	Abnormal head circumference in learning-disabled children	Yes	No	No	Yes	No	No
Smithers, 2013	Impact of neonatal growth on IQ and behavior at early school age.	Yes	No	No	Yes	Yes	No
Stathis, 1999	Head circumference in ELBW babies is associated with learning difficulties and cognition but not ADHD in the school-aged child.	Yes	No	No	Yes	Yes	No
Strauss, 1998	Growth and development of term children born with low birth weight: effects of genetic and environmental factors.	Yes	Yes	No	Yes	No	No
Toro Diaz, 1998	Anthropometric assessment and school	Yes	No	No	Yes	Yes	No

Author, year	*Title*	*Was the sample representative of a well-defined population group?*	*Were socioeconomic level and maternal IQ or maternal schooling included in the adjusted analysis?*	*Were well-defined mediators (i.e., schooling, intelligence, or subsequent head circumference measured between exposure and outcome of interest) included as confounders in the adjusted analysis?*	*Were the measurements of the head circumference standardized for age and sex or were these variables included in the adjusted analysis?*	*Was a measure of association between head circumference and the outcome of interest calculated?*	*Was this article selected for the restricted analysis?*
	achievement in school-age children from high school in Valparaiso, Chile.						
Veena, 2010	Association of Birthweight and Head Circumference at Birth to Cognitive Performance in 9-to 10-Year-Old Children in South India: Prospective Birth Cohort Study	Yes	Yes	No (model 3)	Yes	Yes	Yes
Wang, 2014	The growth of very-low-birth-weight infants at 5 years old in Taiwan.	Yes	No	No	No	No	No
Weinberg, 1974	Intelligence, reading achievement, physical size and social class. A study of St. Louis Caucasian boys aged 8-0 to 9-6 years, attending regular schools.	Yes	No	No	No	No	No
Wright, 2015	Head growth and neurocognitive outcomes.	Yes	No	No	Yes	No	No
Yajnik, 2022	Robust determinants of neurocognitive development in children: evidence from the Pune Maternal Nutrition Study	Yes	No	Yes	No	Yes	No
Yu, 2021	Effect of first-month head-size growth trajectory on cognitive	Yes	No	No	No	Yes	No

Author, year	***Title***	***Was the sample representative of a well-defined population group?***	***Were socioeconomic level and maternal IQ or maternal schooling included in the adjusted analysis?***	***Were well-defined mediators (i.e., schooling, intelligence, or subsequent head circumference measured between exposure and outcome of interest) included as confounders in the adjusted analysis?***	***Were the measurements of the head circumference standardized for age and sex or were these variables included in the adjusted analysis?***	***Was a measure of association between head circumference and the outcome of interest calculated?***	***Was this article selected for the restricted analysis?***
	outcomes in preterm infants.						
Zhu, 2022	Head circumference trajectories during the first two years of life and cognitive development, emotional, and behavior problems in adolescence: a cohort study	Yes	Yes	No	Yes	Yes	Yes
