# Supplementary material for: Head circumference and intelligence, schooling, employment, and income: a systematic review
Source: BMC Pediatr. 2024 Nov 7;24:709. doi: 10.1186/s12887-024-05159-2 (PMC11542250; doi:10.1186/s12887-024-05159-2)
Supplement: Supplementary file 4 — Additional file 4. Details of all included articles. Details of the 115 included articles are provided in this file. [file 12887_2024_5159_MOESM4_ESM.pdf]

Title, author, year, country, study-design	Objective	Study sample [N]	Exposure	Outcomes	Confounders	Main Results
Prenatal head growth and child neuropsychological development at age 14 months **Alamo-Junquera, 2014** Spain Cohort	To assess whether prenatal head growth and HC at birth were prospectively associated with child neuropsychological development at age 14 months in a large population-based birth cohort.	Data from 4 cohorts (Asturias province, Gipúzcoa province, Sabadell city, and Valencia city) of the larger Infancia y Medio Ambiente (Environment and Childhood) (INMA) Project. The study included pregnant women agreed to participate and met the inclusion (>=16 years of age, singleton pregnancy, intention to deliver at the reference hospital) and exclusion (no communication handicap, no fetuses with malformations, no assisted conception) criteria, during their first pre-natal visit, between 2003 and 2008 [N=2104]	Head circumference according to gender and age, categorized into three groups: <10th percentile (n=170), >90th percentile (n=128), and within these 2 cutoff points (n=1729) [at birth].	Intelligence/Cognition: mental scale of the Bayley Scales of Infant Development, version I (BSID), normalized to a mean of 100 points with a SD of 15 points [11-23 months]	Maternal social class, maternal age, maternal BMI, maternal schooling, parity, smooking during pregnancy, paternal BMI, gestational age, sex, country of birth of the father and mother, preterm birth (<37 weeks),cohort location, psychologist.	Linear regression (Betas): Head circumfence <10th percentile: 0.47 (CI95%:0.00;0.94) p<0.05; 10th-90th percentile: 0.00 (CI95%: -0.08;0.08) p≥0.05; > 90th precentile: -0,38(CI95%: -0.93;0.18) p≥0.05
Head circumference at birth and school performance: a nationwide cohort study of 536,921 children **Bach, 2020** Denmark Cohort	To investigate the association between the full range of head circumference at birth, including head circumference relative to birth weight, and school performance in reading and mathematics.	All live births in Denmark from 1 January 1997 to 31 December 2005 in the Danish Medical Birth Registry, singletons with no diagnoses of major malformations, congenital syndromes, or teratogenic conditions, with no record of maternal alcohol exposure during pregnancy, and a gestational age at birth between 22 and 45 weeks [N=536921].	Head circumference z scores according to gender and gestational age, categorized into three groups: <−2 SD, −2 SD to 2 SD (reference group), and >2 SD [at birth]	Academic performance: Part of a national test program introduced in public schools in 2010; Reading abilities are tested every second year (second to eighth grade) and mathematic abilities in the third and sixth grade. The test scores were standardized to a mean of 0 and a SD of 1 [8–15 years].	Socioeconomic level, maternal schooling, parity, smoking during pregnancy, paternal schooling, sex, birth year; origin, age, maternal diseases (hypertension, diabetes, psychiatric disease, antiepileptic use during pregnancy), cohabitation status.	Linear regression (betas): Head circumference <−2 SD: Reading: 2 grade - crude: −0.12 (CI95%:−0.15;-0.10); adjusted: -0.08 (CI95%:-0.10;-0.06); 4 grade - crude: -0.14 (CI95%:-0.16;-0.12); adjusted: -0.08 (CI95%:-0.10;-0.06); 6 grade - crude: -0.13 (CI95%:-0.15;-0.11); adjusted: -0.07 (CI95%:-0.10;-0.05); 8 grade: crude: -0.13 (CI95%:-0.16;-0.10); adjusted: -0.07 (CI95%:-0.10;-0.04); Mathematic: 3 grade - crude: -0.14

Title, author, year, country, study-design	Objective	Study sample [N]	Exposure	Outcomes	Confounders	Main Results
						(CI95%:-0.16;-0.12); adjusted: -0.08 (CI95%:-0.10;-0.06); 6 grade - crude: - 0.15 (CI95%:-0.17;-0.13); adjusted: -0.08 (CI95%:-0.10;-0.06); Head circumference >2 SD: Reading: 2 grade - crude: 0.08 (CI95%:0.06;0.10); adjusted: 0.06 (CI95%:0.04;0.07); 4 grade - crude: 0.08 (CI95%:0.06;0.10); adjusted: 0.05 (CI95%:0.03;0.07); 6 grade - crude: 0.09 (CI95%:0.08;0.11); adjusted: 0.05 (CI95%:0.04;0.07); 8 grade: crude: 0.10 (CI95%:0.07;0.13); adjusted: 0.05(CI95%:0.03;0.08); Mathematic: 3 grade - crude: 0.07 (CI95%:0.05;0.09); adjusted: 0.05 (CI95%:0.03;0.07); 6 grade - crude: 0.11 (CI95%:0.08;0.13); adjusted: 0.06 (CI95%:0.05;0.08);
Correlations between intelligence, head circumference and height: evidence from two samples in Saudi Arabia. **Bakhiet, 2017** Saudi Arabia Cross-sectional	To reports two studies designed to ascertain whether intelligence are associated with head size and height in Saudi Arabia	Sample I consisted of 1591 school students (609 boys and 982 girls) aged 6–12 years (mean 9.5 years) attending state schools in Riyadh, the capital of Saudi Arabia. Sample II consisted of 221 boys aged 6–12 years (mean 8.6 years)	Head circumference (centimeters) [6-12 years]	Intelligence/Cognition: Study I - Standard Progressive Matrices Plus (SPM+); Study II - Standard Progressive Matrices (SPM) [6-12 years]	Gestacional age and sex.	Correlation: Study I: 0.350 p<0.001 (crude) e 0.249 p<0.001 (adjusted); Study II: 0.168 p<0.05 (crude) e 0.173 p<0.001(adjusted);

Title, author, year, country, study-design	Objective	Study sample [N]	Exposure	Outcomes	Confounders	Main Results
		with learning disabilities attending schools in Riyadh. [N=1812]				
Normative data for IQ, height and head circumference for children in Saudi Arabia. **Batterjee, 2013** Saudi Arabia Cross-sectional	To report normative data for intelligence, height and head circumference for 6- to 15-year-olds in Saudi Arabia, and the correlations between these variables.	Representative sample from school students at public and private school in Mecca Province, Saudi Arabia, aged between 6.0 and 15.5 years, between April and June 2010 [N= 1553].	Head circumference [6-15 years]	Intelligence/Cognition: Standard Progressive Matrices (SPM) without a time limit [6-15 years]	No adjustment was performed.	Pearson correlation coefficient: For head circumference, sixteen of the eighteen correlations are positive and three are statistically significant. (stratified by sex and grade); Min: 0.013; Max: 0.410; Mean: 0.138.
Prenatal and early childhood predictors of intelligence quotient (IQ) in 7-year-old Danish children from the Odense Child Cohort **Beck, 2022** Denmark Cohort	To assess IQ scores based on standardized age-appropriate scores from the Danish background population and to evaluate potential predictors of IQ in a large sample of 7-year-old children from the Odense Child Cohort	Newly pregnant women residing in the Municipality of Odense From 2010 to 2012 [N=1375]	Head circumference z scores according to gender and gestational age based on international WHO-UK-growth [at birth, 18 months, 7 years].	Intelligence/Cognition: Wechsler intelligence scale for children 5th edition (WISC-V) - FSIQ, standardized to a mean of 100 points with a SD of 15 points [7 years]	Tester, inclusion date, school grade (Model 1) + maternal education, child sex (Model 2)+ maternal pre-pregnancy BMI, z-score for head circumference at birth and duration of breastfeeding (Model 3).	Linear regression (betas): Head circumference at birth- Model 1: 1.0 (CI95%:0.5; 1.4) p<0.001; Model 2: 0.8 (CI95%:0.4; 1.2) p<0.001; Model 3: 0.9 (CI95%:0.5; 1.4) p<0.001; At 18 months Model 1: 1.8 (CI95%:1.2; 2.5) p<0.001; Model 2: 1.7 (CI95%:1.1; 2.4) p<0.001; Model 3: 1.4 (CI95%:0.7; 2.1) p<0.001; At 7 years - Model 1: 2.1 (CI95%:1.5; 2.7) P<0.001; Model 2: 2.0 (CI95%:1.4; 2.6) p<0.001; Model 3: 1.8 (CI95%:1.1; 2.4) p<0.001;
Infant growth before and after term: effects on neurodevelopment in preterm infants. **Belfort, 2011** Australia Cohort	To identify sensitive periods of postnatal growth for pre-term infants relative to neurodevelopment at 18 months' corrected age; to examine the extent	Data from the DHA for the Improvement of Neurodevelopmental Outcome (DINO) study, a randomized trial of docosahexaenoic acid (DHA) supplementation for preterm infants born at <33 weeks'	Head growth in z scores using World Health Organization standards; The authors used mixed-effects regression models to estimate linear	Intelligence/Cognition: Mental Development Index of Bayley Scales of Infant Development, version II (BSID-II), normalized to a mean of 100 points with a SD of 15 points [18 months' corrected age]	Maternal age, maternal schooling, smoking in pregnancy, paternal schooling, gestacional age, sex, age; chronic lung disease, grade 3 or 4 intraventricular	Linear regression (betas): Week 1 to Term (n=561): 1.4 (CI95%:0.0;2.8); Term to 4 months (n=550): -0.5 (CI95%:-2.2;1.1); 4 to 12 months (n=432): 0.0 (CI 95%:-1.7;1.6); The article also presented stratified analysis for weight at birth,

Title, author, year, country, study-design	Objective	Study sample [N]	Exposure	Outcomes	Confounders	Main Results
	to which associations of growth with neuro-developmental outcomes are stronger for infants more vulnerable to neuro-developmental impairment.	gestation.Participants were recruited from 5 Australian perinatal centers from April 2001 to October 2005. Infants were excluded if they had major congenital or chromosomal abnormalities, were from a multiple birth in which not all live-born infants were eligible or were in other trials of fatty acid supplementation. Lactating mothers in whom tuna oil was contraindicated (for example, because of bleeding disorders or therapy with anticoagulants) were also excluded [N=613].	slopes representing weekly growth rates from 1 week of age to term and then calculated internal z scores for growth rates for each measurement [week 1, at term, 4, 12, and 18 months' corrected age]		hemorrhage, postnatal steroids, breastfeeding status at discharge (never, weaned, mixed, exclusive); Home Screening Questionnaire score, and DINO treatment arm	gestational age and maternal schooling. It seems to exist an interaction effect between head growth from week 1 to term and maternal schooling: <Tertiary: 2.8 (CI95%:1.0;4.6); ≥Tertiary 0.2 (CI95%:-1.9;2.3).
Birth characteristics and risk of low intellectual performance in early adulthood: are the associations confounded by socioeconomic factors in adolescence or familial effects? **Bergvall, 2006** Sweden Cohort	To investigate whether the association between size at birth and intellectual performance in young adulthood is influenced by familial factors, including shared environmental and common genetic factors.	The Swedish Birth Register included information on 458 371 live-born males born between 1973 and 1981. Males who were born to mothers of non-Nordic nationality, before 28 or after 43 completed weeks' gestation, with congenital malformations, and multiple births were excluded. Among the survivors at 18 years, 94% were conscripted between the years 1991 and 2000 [N=357768].	Head circumference z scores according to gestational age, categorized into three groups: <−2 SD, −2 SD to 2 SD (reference group), and >2 SD [at birth].	Intelligence/Cognition: intellectual performance, measured through a time-limited test in 4 dimensions: logical/ inductive, verbal, spatial, and theoretical/technical. The results were presented as standard 9 (stanine) scores. Low intellectual performance was defined as a score of ≤2 [18 year, at conscription for military service]	Socioeconomic level, maternal age, parity, gestational age, weight at birth, lenght at birth, year of conscription, households' family structure, conscription year, growth in height (SD), BMI at conscription.	Logistic Regression (Odds Ratio): Head circumference < -2 SD: crude 1.40 (IC95%:1.33;1.48); adjusted: 1.28 (CI95%:1.20;1.37); >2 SD: crude 0.81 (CI95%:0.75;0.88); adjusted: 0.84(CI95%:0.77;0.91); Additional analysis – familial effects: Between-Families 1.10 (CI95%:1.06;1.13); Within-Families: 1.05 (CI95%:1.01;1.10).

Title, author, year, country, study-design	Objective	Study sample [N]	Exposure	Outcomes	Confounders	Main Results
Risks for low intellectual performance related to being born small for gestational age are modified by gestational age. **Bergvall, 2006** Sweden Cohort	To investigate whether the associations between anthropometric measurements at birth and risks for low intellectual performance in early adulthood are modified by gestational age.	Nonmalformed singleton male individuals to Nordic mothers who were born between 28 and 43 completed weeks of gestation from 1973 to 1981. Data from 4 population-based registries: the Swedish Medical Birth Register, the Swedish Conscript Register, the Multi-Generation Register, and the Population and Housing Census of 1990 [N=352125].	Head circumference z scores according to gestational age, categorized into five groups:< -2 SD, from -2 to -1 SD, from -1 to 1 SD (reference group), from 1 to 2 SD, and >2 SD [at birth].	Intelligence/Cognition: intellectual performance, measured through a time-limited test in 4 dimensions: logical/ inductive, verbal, spatial, and theoretical/technical. The results were presented as standard 9 (stanine) scores. Low intellectual performance was defined as a score of ≤2 [18 year, at conscription for military service]	Socioeconomic level, maternal age, parity,height, year of conscription, household's highest education, household's family structure, birth weight for gestational age; The analysis was stratified for gestational age: preterm (28 to 36 weeks), term (37 to 41 weeks) and postterm (42 to 43 weeks).	Logistic Regression (Odds Ratio): Preterm - Head circumference <-2 SD: 1.89 (CI95%:1.36;2.62); from -2 to -1 SD: 1.21(Ci95%:1.00;1.47); from 1 to 2 SD: 0.80 (CI95%:0.66;0.97); > 2 SD: 0.77 (CI95%:0.50;1.17); Term - <-2 SD: 1.24 (CI95%:1.16;1.33); from -2 to -1 SD: 1.12 (CI95%:1.08;1.17); from 1 to 2 SD: 0.98 (CI95%:0.94;1.02); > 2 SD: 0.86 (CI95%:0.78;0.95); Postterm - <-2 SD: 1.00 (CI95%:0.79;1.26); from -2 to -1 SD:1.00 (CI95%:0.91;1.09); from 1 to 2 SD: 1.01 (CI95%:0.92;1.11); > 2 SD: 1.09 (CI95%:0.84;1.42).
Correlational analyses of the influence of basal chronological age on IQ relationships to specified anthropometric measurements. **Boynton, 1942** United States Cross-sectional	To identify most valid basal age to use in the computation of intelligence quotient and its relationships (physical record)	Adolescents from 14 to 20 years in age, who graduated in the years 1933, 1934, 1935, 1937, 1939, and 1940 from the Peabody Demonstration School [N=199].	Head circumference [14-20 years]	Intelligence/Cognition: Kuhlmann-Anderson Test - QI for different "basal ages" [14-20 years]	No adjustment was performed.	Correlation: Boys - 14y: 0.26; 15y: 0.27; 16y: 0.23; Girls - 14y: 0.04; 15y: 0.03; 16y: 0.18
Impact of Early Nutrient Intake and First Year Growth on Neurodevelopment	To assess the relationship between nutritional intake during the first 28 days and weight, length, and	The study was conducted at the Hospital of Lithuanian University of Health Sciences, Department of Neonatology. Inclusion	Head circumference z-score adjusted for age and sex [weekly from birth to discharge	Intelligence/Cognition: Bayley Scales of Infant Development, Second Edition (BSID-II) - Mental Development Index scores normalized to mean = 100	Weight, height, gestational age, sex, total protein, total carbohydrates and total fat in g/kg/day.	Linear Regression (Betas): Change of head circumference in SD from birth to discharge – 23-27 weeks (N=41): 0.07 p=0.696; 28-34 weeks

Title, author, year, country, study-design	Objective	Study sample [N]	Exposure	Outcomes	Confounders	Main Results
of Very Low Birth Weight Newborns **Brinkis, 2022** Lithuania Cohort	head circumference growth during the first year of life and neurodevelopment at 12 months of corrected gestational age of very low birth weight infants receiving early progressive enteral feeding.	criteria for study participants were birth weight < 1500 g and GA ≤ 34 weeks, and written consent of both parents obtained. Exclusion criteria were chromosomal abnormalities, genetic syndromes which may affect growth, absent parental consent, and surgical intervention with partial bowel removal [N=95].	and at 3, 6, 9, and 12 months].	and SD = 15, and classified as Mild development delay if between −1 SD and −2 SD; moderate delay if between −2 SD and −3 SD, and severe delay if < -3 SD [12 months].		(N=53): 0.14 p=0.475; From discharge to 12 months - 23-27 weeks (N=41): 0.28 p=0.227; 28-34 weeks (N=53): 0.37 p=0.056
The influence of birth size on intelligence in healthy children. **Broekman, 2009** Singapore Cohort	To examine the association between birth lenght, birth weight, head circumference, and gestational age at birth, as surrogates of intrauterine fetal growth, to childhood IQ in a large cohort of healthy Singapore children of normal birth size.	Asian children who were attending grades from 1 to 3 (7–9 years old) at 3 "normal" schools in different parts of Singapore between November 1999 and May 2001. Children with serious chronic medical conditions (eg, heart disorders, cancer, and chronic eye conditions) were excluded. Data from Singapore Cohort Study of Risk Factors for Myopia [N=1645].	Head Circumference (centimeters) - Birth history data were obtained from documented medical chart booklets [at birth].	Intelligence/Cognition: Raven's Standard Progressive Matrices (RPM) [Follow-up in 2002, around 9 years]	Age, gender, ethnicity, school, and mother's education (Model 1) + BMI, mother's age at birth, mother's and father's smoking, family size, birth order, and GA (Model 2).	Linear Regressions (Betas): Model 1: 0.42 (CI 95%:0.08;0.75) p=0.015; Model 2: 0.62 (CI 95%:0.21;1.04) p=0.003; Additional analysis among children with normal weight (<2,5 e >4 kg), normal head circumference (<32 e >36 cm) and normal gestational age (<37 weeks): 0.96 (CI95%:0.26;1,66) p valor= 0,008
Early life determinants of low IQ at age 6 in children from the 2004 Pelotas Birth Cohort: a predictive approach. **Camargo-Figuera, 2014** Brazil Cohort	To identify early life determinants of low IQ at age 6 using a predictive modeling approach.	All live births of mothers living in the urban area of Pelotas, in 2004. Children with serious conditions that can be associated with very low IQ (e.g., severe mental retardation and cerebral palsy) were excluded [N=3523].	Head circumference-for-age deficit during the first year of life, based on the World Health Organization growth chart. Deficits were defined as a z-	Intelligence/Cognition: Low IQ, defined as IQ z-score < −1; Wechsler Intelligence Scale for Children-III (WISC-III) - short-form version composed of 4 subtests: 2 verbal (similarities and arithmetic) and 2 performance (block	Socioeconomic level, maternal schooling, sex, age, breastfeeding, interview setting, IQ test evaluator; skin color: both mother and father non-white, father unemployed at the child's birth, mother unemployed	Logistic Regression (Odds Ratio): Crude: 2.4(CI95%:1.9;3.2) p<0.00001; Adjusted: 1.7(CI95%:1.2;2.4) p=0.0022;

Title, author, year, country, study-design	Objective	Study sample [N]	Exposure	Outcomes	Confounders	Main Results
			score < −2 SD at any of the three follow-ups [perinatal, 3 months and 12 months].	building and picture completion) [6 years].	during the child's first 12 months of life; number of siblings at the child's birth, number of persons per room at age 12 month, at least one smoking parent during pregnancy, height-for-age deficit during the first year of life; maternal perception of the child's health status.	
Maternal and neonatal risk factors for mental retardation: defining the 'at-risk' child. **Camp, 1998** United States Cohort	To determine how mental retardation at age seven is related to certain maternal, perinatal, and neonatal characteristics.	Children's mothers had enrolled when registering for prenatal care in one of the 12 urban medical centers that made up the maternal and child data collection sites. Most sites were in cities in the northeast or the southern region of the United States. Children with down syndrome, other genetic or post-infection syndromes (neurofibromatosis, post-rubella, post-meningitic encephalitis), major malformations of the CNS (hydrocephaly, microcephaly), other major CNS disorders (cerebral palsy, epilepsy and rarer major sensory and post-traumatic deficits), neonatal seizures, brain	Head circumference < 31 centimeters [at birth].	Intelligence/Cognition: IQ score < 70 on the Wechsler Intelligence Scale for Children (WISC) - abbreviated version (4 verbal and 3 performance subtests) [7 years].	No adjustment was performed, but the analys was stratified for socioeconomic level and color of skion.	Risk Ratio (Chi-sqaured Test): Low socioeconomic level and black: 2.35 p<0.001; Medium/Hight socioeconomic level and black: 0.39 p>0.05; Low socioeconomic level and white: 0.96 p>0.05; Medium/Hight socioeconomic level and white: 2.63 p>0.05.

Title, author, year, country, study-design	Objective	Study sample [N]	Exposure	Outcomes	Confounders	Main Results
		abnormality (a diagnosis based on abnormal movements, tone, or reflexes observed in the newborn), and birth weight less than 2000 g, were excluded [N=35704].				
An evaluation of various parameters of maturity at birth as predictors of development at one year of life **Caputo, 1974** United States Cohort	To assess the comparative value of several measures of prematurity, immaturity, or underdevelopment of the neonate for predicting developmental status of the infant at 1 year of age.	Infants who were born between 1400 and 2500 g on Staten Island between July 1965 and January 1969 at the three voluntary hospitals on Staten Island. Infants suffering serious physical disabilities and children of unwed and divorced mothers were excluded. From hospital records, a group of full-sited infants (weighing over 2500 g at birth) was matched to the group of premature infants on sex, hospital of birth, socioeconomic status, parity, and date of birth [N=233].	Head circumference in inches [at birth].	Intelligence/Cognition: Cattell Infant Intelligence Scale [1 year].	Socioeconomic level, maternal age, maternal IQ, parity, height, gestational age, sex, weight at birth, Lubchenco score, reflex, Apgar score, hospital e type of delivery.	Correlation: 0.39 ($p < 0.01$), Partial correlation: 0.01 ($p > 0.05$)
Very preterm children free of disability or delay at age 2: predictors of schooling at age 8: a population-based longitudinal study. **Charkaluk, 2011** France Cohort	To study the predictive value of a developmental assessment at 2 years corrected age for schooling at age 8 in children born very preterm and free of disability or delay; to identify other factors associated with	All births between 22 and 32 completed weeks of gestation in all maternity units in the Nord-Pas de Calais region in 1997 who underwent a physical examination and a psychometric assessment at age 2 and were considered free of disability or delay,	Head circumference z-cores according to age and gender, based on World Health Organisation reference curves, categorized into two groups: <-1 SD and $\geq$-1 SD (reference group)	Academic performance: Postal questionnaire sent to the parents with questions about schooling, dicotomized into schooling appropriate for age - being in age-appropriate grade level in a regular classroom environment without support at school, and Schooling not appropriate	Maternal schooling, gestational age, global Developmental Quotient (revised Brunet-Lezine test at 2 years corrected age).	Logistic Regression (Odds Ratio): 2.85 (CI95%: 1.01;8.08) $p<0.05$

Title, author, year, country, study-design	Objective	Study sample [N]	Exposure	Outcomes	Confounders	Main Results
	schooling in this population.	defined as a global DQ less than 70 in the absence of disability [N= 244].	[2 years of corrected age].	for age - being in age-appropriate grade level in a regular classroom environment with support at school, or in regular classroom environment with grade retained, or in a specialised class or school [8 years].		
Associations between preterm birth, small-for-gestational age, and neonatal morbidity and cognitive function among school-age children in Nepal. **Christian, 2014** Nepal Cohort	To examine the long term consequences of low birth weight, preterm birth, small-for-gestational age, neonatal sepsis and birth asphyxia on cognitive and motor function in surviving school age children 7–9 y of age.	Children born to women who lived in rural southern plains of Nepal and participated in a double-blind randomized controlled trial (RCT) of micronutrient supplementation during pregnancy (1999–2001) and who themselves were participants in a RCT of micronutrient supplementation during their preschool years (2001–2005) were tracked and followed at 7–9 y of age (2007–2009). The study included the arms which had received supplementation with iron-folic acid and/or zinc, because they had no impact on the outcomes [N=1822].	Head circumference in centimeters [at birth].	Intelligence/Cognition: Universal Nonverbal Intelligence Test (UNIT) [7-9 anos].	Socioeconomic level, maternal IQ, maternal schooling, parity, weight, age, sex, preterm, small for gestational age, sepsis, birth asphyxia, ever started school, intake of dark green leafy vegetables, intake of citrus, history of diarrhea/dysentery, household salt iodine level.	Linear Regression (Beta): Crude: 1.11 p<0.001; Adjusted: 0.44 p<0.001
Perinatal and postnatal factors in very preterm infants and subsequent cognitive and motor abilities. **Cooke, 2005** United Kingdom	To examine the associations between perinatal and postnatal factors and cognitive and motor abilities at age 7 years in a	All infants born before 32 completed weeks during 1991–92 in the eight hospitals that then existed in the Liverpool postal districts were ascertained. Those who died before discharge	Head circumference percentile, according to gender and age [at birth, 7 years]	Intelligence/Cognition: Wechsler intelligence scale for children (WISC III), dicotomized into < and ≥ 89 (mean) [7 years].	Gestational age, persistence of the arterial duct	Multiple Regression - Head circumference at 7 yeas: Beta: 0.06 p= 0.041; Odds Ratio: 1.06 (CI 95%:0; 1.1); Head circumference was not significative in bivariate analysis (p=0.38)

Title, author, year, country, study-design	Objective	Study sample [N]	Exposure	Outcomes	Confounders	Main Results
Cohort	geographically selected cohort of very preterm infants born over a two year period, in a period of modern perinatal care practices.	from hospital or whose mothers were not resident within a Liverpool postal district at the time of birth were excluded. They were free of major neurodisability and attending mainstream schools [N=280].				and was not included in regression model.
Are there critical periods for brain growth in children born preterm? **Cooke, 2006** United Kingdom Cohort	To identify periods during early development, in children born preterm, when impaired head growth may influence minor motor and cognitive function.	All infants of birth weight 1500 g or less born in 1980 and 1981, to mothers whose place of residence at the time of birth was the county of Merseyside. The obstetric and neonatal records were abstracted for general and clinical details of mother and child. Kids that had cerebral palsy or major visual or hearing deficits were excluded [N=194].	Head circumference z-score, according to gender and age [at birth, at term, at discharge, 4 and 15 years].	Intelligence/Cognition: Wechsler intelligence scale for children (WISC III), dicotomized into < and ≥ 89 (mean) [8 years].	Weight at birth, social class (Registrar General's classification).	Correlations between the outcome and both head circumfence and head growth in each period: crude correlations varied from 0.10 to 0.25 and adjusted from 0.09 to 0.20. For changes in z-score, varied from -0.04 to 0.14 (crude) and from -0.05 to 0.10 (adjusted).
Associations of head circumference at birth with earlylife school performance and later-life occupational prestige **Dekhtyar, 2015** Sweden Cohort	To investigate whether in-utero brain development, measured by head circumfernce at birth, affects (i) school grades reported at age 9-10 and (ii) later-life status attainment captured by occupational prestige, in the same individuals followed-up over the life course. We assess both direct	The Uppsala Birth Cohort Multigenerational Study comprises all live births at the Uppsala University Hospital between 1915 and 1929, who were alive and resident in Sweden in 1960, constituting the population assessed for eligibility, for whom record linkage provided detailed information over their lives. Multiple births, pregnancies lasting less	Head circumference z scores according to gestational age, categorized into three groups: <−1 SD, −1 SD to 1 SD (reference group), and >2 SD [at birth]	Academic performance - Early-life school grades: grades at arithmetic and geometry, writing and grammar, speech and reading, Christian religion studies, handwriting, local geography and history collected during the spring term of elementary school's third year, re-coded the marks from 0 (Grade C) to 18 (Grade A) in accordance with the scoring system suggested by the Swedish education authorities in 1942. They	Sex and birth cohort (Model 1) + birth order, maternal age, weight for gestational age at birth (Modelo 2) + social origin at birth (advantaged vs disadvantaged)	Linear regression (betas): Academic performance: Head circumfernce <−1 SD: model 1: -0.119 (CI95%: -0.177; -0.061) p<0.01; model 2: -0.102 (CI95%:-0.163; -0.043) p<0.01; model 3: -0.097(Ci95%:-0.157; -0.037) p<0.01; > 1 SD: model 1: 0.031 (CI95%: -0.023; 0.084) p≥0.1; model 2: 0.035 (CI95%: -0.021; 0.092) p≥0.1; model 3: 0.031 (CI95%:-0.025; 0.087) p≥0.1 Occupational Prestige:

Title, author, year, country, study-design	Objective	Study sample [N]	Exposure	Outcomes	Confounders	Main Results
	and indirect effects, as well as explicitly test whether social origin is a confounder, or rather an effect modifier, for the link between head circumference at birth, school grades in childhood, and later-life status attainment.	than 37 weeks or more than 41 as well as unknown gestation durations were excluded [N=6024].		calculated an overall third grade mean score after standardizing marks in each subject individually [9-10 years].<br>Employment - Adult occupational prestige: They measured individuals' lifetime status attainment through a prestige score associated with their longest-held occupation in adulthood, using the Standard International Occupational Prestige Scale (SIOPS), which is a continuous scale (range 6-78) that emphasizes subjective perceptions of social rewards, such as approval, respect, admiration, and contempt inherent in occupations [adulthood].		Head circumference <−1 SD: model 1: -1.805 (CI95%:-2.688; -0.921) p<0.01; model 2: -1.636 (CI95%:-2.571; -0.701) p<0.05; model 3: -1.551 (CI95%:-2.484; -0.619) p<0.05; > 1 SD: model 1: -0.355 (CI95%:-1.201; 0.490) p≥0.1; model 2: -0.434 (CI95%:-1.301; 0.432) p≥0.1; model 3: -0.499(CI95%:-1.361; 0.363) p≥0.1<br>No evidence of interaction effect for academic performance (p = 0.14) or occupational prestige (p=0.59). In mediation analysis, 1/4 of the total effect of head circumference on occupational prestige was mediated by school grades.
Poor Head Growth Is Associated with Later Mental Delay among Vietnamese Preterm Infants: A Follow-up Study.<br>**Do, 2021**<br>Vietnam<br>Cohort	To describe the growth of Vietnamese preterm infants in the first 2 years, and to compare with references: World Health Organization (WHO) child growth standards, and healthy Southeast Asian infants. Further, to assess the association between growth in the first year and neurodevelopment	All preterm newborns discharged from the Neonatal Intensive Care Unit at Children's Hospital 1 in Ho Chi Minh City, between July 2013 and September 2014 were eligible for enrolment if they fulfilled the inclusion criteria: <37 completed weeks of gestation at birth and age at admission <29 days. Exclusion criteria were congenital brain malformation and chromosome anomaly [N=143].	Head circumference z-score, Decrement of head circumference z-score [3 and 12 months]	Intelligence/Cognition: Bayley Scales of Infant and Toddler Development - 3rd Edition, including cognitive, receptive and expressive language. Mental delay was defined as cognitive composite scores <-2 SDs) [24 months corrected age]	Gestacional age and sex.	Logistic Regression (Odds Ratio): Head circumference at 3 months: 0.79 (CI95%:0.49;1.29); Decrement of head circumference between 3-12 months:1.89 (CI95%:1.02;3.50)

Title, author, year, country, study-design	Objective	Study sample [N]	Exposure	Outcomes	Confounders	Main Results
	at 2 years corrected age.					
The predictive value of microcephaly during the first year of life for mental retardation at seven years. **Dolk, 1991** United States Cohort	To investigate a group of children who have consistently heads circumference 3 SD below the mean on repeated measurements in infancy, following these children through to the age of seven years, when assessment of IQ is more stable. Other variables that might affect the predictive value of small head size for mental retardation were assessed at the same time, including body size, socio-economic status and the presence of other conditions associated with brain pathology.	Data from US National Collaborative Perinatal Project cohort, which registered pregnancies at 12 hospitals from 1959 to 1966 were followed through to birth, and liveborn children to seven years of age. The subcohort chosen for analysis consisted of all liveborn singletons surviving to at least to one month of age, of black or white ethnic origin, who were products of 'core' pregnancies (i.e. not selectively referred to the hospital for specialist care). Cases of spina bifida, encephalocele, hydranencephaly, hydrocephaly, craniosynostosis and Down syndrome were excluded [N= 35710].	Head circumference z scores according to age, sex and ethnicity, categorized into three groups: <−3 SD, from -2 to -3 SD, > -2 SD (reference group) [at birth, 1 and 7 years]	Intelligence/Cognition:IQ < 70, using Wechsler Intelligence Scale or the Leiter Scale, in cases of deafness [7 years].	No adjustment was performed, but the analys was stratified for socioeconomic level weight at birth and height.	% IQ <70: Head circumference > -2 SD: 2.6%; PC from -2 to -3 SD: 10.5%; <-3 SD: 51,2%; In text: The socioeconomic level was an important predictor of the outcome in groups with head circumference > -2 and from -2 to -3 SD.
The Predictive Value of Head Circumference Growth during the First Year of Life on Early Child Traits. **Dupont, 2018** Canada	To investigate the predictive value of head circumference growth trajectory in the first year of life on early child development at 24 months of age in a	Data from Design, Develop, Discover (3D) cohort study, which recruited pregnant at nine urban clinical centers in three metropolitan areas in the province of Quebec.	Head circumfence - latent-growth curve models (LGCM) to assess HC development during the first year of life, modeling both the	Intelligence/Cognition: Bayley Scales of Infant and Toddler Development, Third Edition. The Cognitive scale of the Bayley-III contains 91 items assessing information processing,	Socioeconomic level, maternal schooling, gestational age.	Trajectory analysis (Beta): Boys: Head circumference at birth: −0.09; From birth to 12 months: 0.12; Girls:Head circumference at birth: 0.06; From birth to 12 months: 0.03; p>0.05 for all measures of effect

Title, author, year, country, study-design	Objective	Study sample [N]	Exposure	Outcomes	Confounders	Main Results
Cohort	healthy general population sample.	Infants who were born before 34 weeks of gestational age, presented serious health conditions, neurological insults or known risk factors that could affect normal development, and whose mother used drugs during pregnancy, were excluded [N= 756].	intercept (HC at birth) and slope (HC growth from 0 to 12 months), stratified for sex [at birth, 3 and 12 months]	conceptual resources and perceptual skills [24 months].		
Predictors of intelligence at the age of 5: family, pregnancy and birth characteristics, postnatal influences, and postnatal growth. **Eriksen, 2013** Denmark Cohort	To conduct a systematic evaluation of a broad selection of both well-established and less well-investigated predictors of IQ in a large sample of basically healthy, 5-year-old children selected from the Danish National Birth Cohort; to identify variables that explained variance in addition to the variance explained by maternal IQ and parental education in this non-clinical sample	Data from Lifestyle During Pregnancy Study (LDPS), which recruited women in the Danish National Birth Cohort at their first antenatal visit at a general practitioner, from 1997–2003.Exclusion criteria were multiple pregnancies, inability to speak Danish, impaired hearing or vision likely to compromise the ability to perform the cognitive tests, and congenital disabilities that imply or are likely to imply mental retardation (e.g. trisomy 21 or infantile autism). Based on their alcohol-drinking pattern before and during pregnancy, oversampling was done for women with moderate and higher levels of alcohol consumption (defined in this study as 5–8 drinks	Head circumference in centimeters; analysis was also condcuted using a quadratic term [at birth and 5 years].	Intelligence/Cognition: IQ was assessed with the Wechsler Primary and Preschool Scales of Intelligence - Revised (WPPSI-R). The short form used in the present study included three verbal subtests (Arithmetic, Information, and Vocabulary) and three performance subtests (Block Design, Geometric Design, and Object Assembly). Swedish norms were used to derive scaled scores and IQs [5 years].	Age, sex, tester (partial adjustment) + maternal age, maternal BMI, maternal IQ, parity, paternal age, height, breatfeeding, parental education, maternal marital status, weight at birth, irregular breakfast, postnatal growth (Full adjustment)	Pearson Correlation Coefficient: Head circumference at birth: 0.07 p=0.064; at 5 years: 0.15 p=0.001 Linear Regression (Betas): Partial adjustment: Head circumference at 5 years - Total IQ total: 0.8 (CI95%:0.2; 1.3) p=0.010; Verbal IQ 0.3 (CI95%: -0.3; 0.8) p= 0.354; Performance IQ: 1.3(CI95%: 0.4; 2.1) p= 0.002; Head circumference at 5 years - quadratic term - Total IQ: -0.3(CI95%: -0.6; 0.0) p= 0.045; Verbal IQ: -0.2 (CI95%: -0.4; 0.1) p= 0.268; Performance IQ: -0.4 (IC95%: -0.8; -0.1) p=0.018; Full Adjustment: Head circumference at 5 years – Total IQ: 0.7 (CI95%:0.1; 1.2) p= 0.017; Verbal IQ: 0.3 (CI95%:-0.2; 0.8 p=0.266; Performance IQ: 1.1 (IC95%: 0.3; 1.8) p=0.009 ; Head circumference at 5 years -

**Title, author, year, country, study-design**	**Objective**	**Study sample [N]**	**Exposure**	**Outcomes**	**Confounders**	**Main Results**
		and ≥9 drinks per week). All statistical analyses were weighted by sampling probabilities [N= 1782].				quadratic term – Total IQ: -0.3 (CI95%: -0.6; 0.0) p=0.020 ; Verbal IQ: -0.2 (CI95%: -0.4; 0.1) p= 0.161; Performance IQ: -0.4(CI95%: -0.8; -0.1) p=0.009
Growth patterns in children with intrauterine growth retardation and their correlation to neurocognitive development. **Fattal-Valevski, 2009** Israel Cohort	To document the growth pattern in a cohort of intrauterine growth-retarded children, specify subgroups of catch-up growth versus noncatch-up growth at various ages, and compare the neurodevelopmental and cognitive outcome of these children.	All infants born consecutively after September 1992 at the Lis Maternity Hospital, Tel Aviv Sourasky Medical Center, with a birth weight <10th percentile for gestational age, according to Israeli birth weight. Children with genetic syndromes, major malformations, or congenital infections were excluded [N= 136].	Head circuference [at birth, 1, 2, 6 and 9-10 years)	Intelligence/Cognition: Wechsler Intelligence Scale for Children-Revised [9-10 years]	Maternal and paternal height.	Pearson Correlation: "Head circumference at birth did not correlate with neurodevelopmental and IQ scores at 9 to 10 years."; The values were presented in a chart and are, aproxemately: head circumference at 1 year: 0.27 p < 0.01; at 2 years: 0.30 p < 0.01; at 6 years: 0.32 p < 0.01; at 9-10 years: 0.35 p < 0.01
Head circumference and child ADHD symptoms and cognitive functioning: results from a large population-based cohort study **Ferrer, 2019** Spain Coorte	To understand the association between prenatal, newborn and postnatal head circumference and preschool neurodevelopment in a large population-based birth cohort.	This study was based on four cohorts (Asturias, Gipuzcoa, Sabadell and Valencia) of the larger Infancia y Medio Ambiente [environment and childhood] Project, established between 2004 and 2008. Mothers were considered eligible for inclusion if they were residents in the cohort area, at least 16 years old, were carrying a singleton pregnancy and were planning to give birth at the reference hospital. Mothers who had participated in an	Head circumference z scores according to age and sex [at birth, 1-1.5 and 4 years].	Intelligence/Cognition: McCarthy Scales of Children's Abilities (MCSA global cognitive scale) [at 5 years]	Maternal age, maternal BMI, maternal schooling, parity, smooking during pregnancy, paternal BMI, gestacional age, sex, Country at birth (mother and father); social class, age when test was performed, quality of the test performance flagged by the psychologist.	Linear Regressions (Betas): Birth 1.22 (IC95%:0.59;1.85) p < 0.05; 1–1.5 years 1.13 (IC95%:0.10;2.15) p < 0.05; 4 years 0.95 (IC95%:0.15;1.75) p < 0.05

Title, author, year, country, study-design	Objective	Study sample [N]	Exposure	Outcomes	Confounders	Main Results
		assisted fertility program and those with communication difficulties were excluded [N= 1795].				
Early life predictors of intelligence in young adulthood and middle age. **Flensborg-Madsen, 2020** Denmark Coorte	To conduct a systematic evaluation of a broad selec- tion of both well-established and less well-established predictors of IQ in the Copenhagen Peri- natal Cohort.	Data from Copenhagen Perinatal Cohort, which consists of children born at the Copenhagen University Hospital between October 1959 and December 1961. Twins were excluded. WAIS sample: individuals from the CPC who participated in the Prenatal Development Project (PDP) follow-up study conducted in 1982–1994, mean age 27.7 years [N = 1126]; IST-2000R sample: comprised individuals from the CPC who participated in the Copenhagen Aging and Midlife Biobank (CAMB) during the period from 2009 to 2011, mean age 50.0 years [N= 1334]; BPP sample: men from the CPC who appeared before the draft board, mean age 19.2 [N= 2237].	Head circumference [at birth], head growht [from birth to 1 year, from 1 to 3 years]	Intelligence/Cognition: WAIS sample: Wechsler Adult Intelligence Scale (WAIS), including all the 11 subtests [mean age 27.7 years]; IST-2000R sample: Intelligenz-Struktur-Test 2000R, self-administered test which consists of three subtests (sentence completion, verbal analogies, and number series), providing a total score ranging from 0 to 59 [mean age 50 years]; BPP sample: Børge Priens Prøve, group test with four subtests (letter matrices, verbal analogies, number series, and geometric figures) providing a total score ranging from 0 to 78 [mean age 19.2 years].	Socioeconomic level, maternal height, parity, smoking during pregnancy, paternal age, gestational age/age, sex, breastfeeding, Single mother prenatally, Pregnancy complications, Birth weight and length, Mother's attitude towards pregnancy (wanted), Mother's employment (employed at 1-year), Daycare institution at some point in the first year, weight and length increase during first year and age 1-3, 1 an 3-year milestones mean.	Linear regression (Betas): Head circumference at birth: WAIS 0.14 $p<0.01$; IST-2000R 0.17 $p<0.01$; BPP 0.16 $p<0.001$; Head growth from birth to 1 year: WAIS 0.18 $p<0.001$; IST-2000R 0.16 $p<0.01$; BPP 0.17 $p<0.001$; Heads growht from 1 to 3 years: WAIS 0.10 $p<0.05$; IST-2000R 0.02 $p>0.05$; BPP 0.11 $p<0.01$
The importance of head growth patterns in predicting the cognitive abilities and literacy skills of	To evaluate the effects of head growth compromise beginning in utero and continuing, in	Small for gestational age (weight for gestational age <-2 SD at birth) enrolled in the Integrated Perinatal	Head circumference, dicomized into head circumference >	Intelligence/Cognition: Wechsler Intelligence Scales For Children–Third Edition (WISC–III) - short form; Academic	No adjustment was performed.	Means (Chi-squared, Anova, MANOVA; Tukey's Honestly Significant Difference post hoc tests) Total IQ:

Title, author, year, country, study-design	Objective	Study sample [N]	Exposure	Outcomes	Confounders	Main Results
small-for-gestational-age children. **Frisk, 2002** Canada Coorte	some cases, through the first 9 months of life on the cognitive and literacy skills of school-age small-for-gestational-age children.	Follow-up Program for Mt. Sinai Hospital and the Hospital for Sick Children in Toronto, Canada, between 1984 and 1987. Exclusion criteria were intrauterine growth restriction arising from a congenital infection or genetic anomaly or alcohol and drug abuse, presence of confounding medical conditions after birth (e.g., seizure disorders, hepatitis, cardiac abnormalities, and hearing impairments), limited knowledge of English at the time of assessment [N=71].	10th percentile (reference group) and head circumference ≤ 10th percentile). Then, children were categorized into three groups: normal SGA group: head circumference > 10th percentile at birth and > 10th percentile at 9 months; catch-up SGA group: head circumference ≤ 10th percentile at birth > 10th percentile at 9 months; abnormal SGA group: head circumference ≤ 10th percentile at birth and ≤ 10th percentile at 9 months of age [at birth, 9 months].	performance: Reading - Woodcock Reading Mastery Tests Revised, Word identification subtest and Passage comprehension subtest, Backman word lists; Spelling - Kaufman Tests of Educational Achievement or Wide Range Achievement Test–3, spelling subtest (7-9 anos)		Normal: 106.2; Catch-up: 92.7; Abnormal: 89.6; IQ Verbal: Normal: 103.9; Catch-up: 92.8; Abnormal: 92.4; Performance IQ: Normal: 107.7; Catch-up: 94.5; Abnormal: 88.7; Reading: Word identification: Normal: 105.1; Catch-up: 91.6; Abnormal: 90.1; Passage comprehension: Normal: 99.2; Catch-up: 82.6; Abnormal: 85.6; Backman word lists: Normal: 88.4; Catch-up: 68.3; Abnormal: 60.0; Spelling: Normal: 96.2; Catch-up: 89.5; Abnormal SGA: 88.7 (significative differences according to the text, but p values were not presented).
Foetal and postnatal head growth and risk of cognitive decline in old age. **Gale, 2003** United Kingdom Cohort	To investigate whether brain growth in foetal or postnatal life, as indicated by head circumference, affected the risk of cognitive decline in old age.	Infants who were born at Jessop Hospital for Women between 1922 and 1930 and still lived in Sheffield in 1997-1998. A stratified sample of 746 people, comprising all 236 subjects from the highest and lowest fifths of birth weight and 85 randomly chosen subjects of each sex from each of the three intervening fifths of	Head circumference - quarters of the distribution, treating men and women separately [at birth].	Intelligence/Cognition: AH4 intelligence test, which provides a measure of logical, verbal and numerical reasoning and The Logical Memory subtest of the Wechsler Memory Scale, which assesses the ability to recall ideas presented in two short stories [Follow-ups of 1997-1998 - mean age 69.8 years, and 2000-2001].	Social class at birth, gestational age, age at follow-up, sex, education, history of cerebrovascular disease, Nottingham Health Profille emotion subscale score.	Linear Regression (Mean for head circumference quarters at birth): AH4 intelligence test: Inicial score: Q1: 23.6 ; Q2: 23.4 ; Q3: 23.6 ; Q4:25.1, p=0.376; Follow-up score: Q1: 26.2; Q2: 26.1; Q3: 25.6; Q4: 25.8, p=0.943; Logical Memory subtest: Inicial score: Q1: 23.7; Q2: 24.4; Q3: 23.8; Q4: 23.8, p=0.746; Follow-up score: Q1: 23.6; Q2: 24.2; Q3: 23.2; Q4: 22.2, p=0.508

Title, author, year, country, study-design	Objective	Study sample [N]	Exposure	Outcomes	Confounders	Main Results
		birth weight, was selected. Four hundred and twelve were included in 1997-1998 follow-up and 242 agreed to be visited again in 2000-2001. Participants with missing data on head circumference or who had experienced problems during the cognitive function testing due to interruption or deafness were excluded [215].				
Critical periods of brain growth and cognitive function in children. **Gale, 2004** United Kingdom Cohort	To investigate whether brain growth during different periods of pre and postnatal development influences later cognitive function in a group of children for whom serial measurements of head growth through foetal life, infancy and childhood were available.	Singleton children born to Caucasian women aged 16 years or older who registered under two obstetric consultants and who attended the midwives' antenatal booking clinic at the Princess Anne Maternity Hospital in Southampton at <17 weeks gestation between April 1992 and June 1993, who were still living in the Southampton area around their ninth birthday [N=221].	Head circumference z-score according to age [at birth, 9 months and 9 years].	Intelligence/Cognition: Wechsler Abbreviated Intelligence Scale - Abbreviated [9 years]	Maternal age, maternal IQ, maternal schooling, sex, breastfeeding, social class; low mood post-partum; number of older siblings.	Linear Regressions (Betas): Head circumference at birth: 0.16 (CI95%:-2.00;2.33) p=0.883; at 9 months: 1.98 (CI95%:0.34;3.62) p=0.018; at 9 years: 2.87 (CI95%:1.05;4.69) p=0.002 Head growth from birth to 9 months and Total IQ total: 2.30 (CI95%:0.56;4.03) p= 0.010; Verbal IQ: 1.72 (CI 95%:0.16;3.60) p= 0.072; Performance IQ: 2.49 (CI95%:0.57;4.40) p= 0.011; from 9 months to 9 years and Total IQ: 2.12 (CI 95%:0.39; 3.86) p= 0.017; Verbal IQ: 2.08 (CI95%:0.21;3.95) p= 0.030; Performance IQ: (CI 95%: 1.76 (0.15;3.67) p= 0.070

Title, author, year, country, study-design	Objective	Study sample [N]	Exposure	Outcomes	Confounders	Main Results
The influence of head growth in fetal life, infancy, and childhood on intelligence at the ages of 4 and 8 years. **Gale, 2006** United Kingdom Cohort	To investigate the effects of brain growth prenatally, during infancy, and during later periods of postnatal development on cognitive function at the ages of 4 and 8 years among term-born members of the Children in Focus subset of the Avon Longitudinal Study of Parents and Children (ALSPAC) cohort whose head circumference was measured at birth and at regular intervals thereafter.	Data from ALSPAC, a prospective study which recruited all pregnancies in 3 Bristol-based district health authorities with expected dates of delivery between April 1991 and December 1992. The Children in Focus cohort is a 10% random selection from the last 6 months of ALSPAC births and includes only term-born children [N=633].	Head circumference z-score according to sex and gestational age; Postnatal head growth between successive time points, conditional on previous size, was calculated by saving the residuals from linear regression models of head circumference SDS at each successive time point versus head circumference SDS at all earlier time points [at birth, 1, 4 and 8 years].	Intelligence/Cognition: Wechsler Preschool and Primary Scale of Intelligence [4 years],Wechsler Intelligence Scale for Children [8 years].	Socioeconomic level, maternal age, maternal schooling, number of older siblings, paternal schooling, gestational age, sex, breastfeeding, parenting score (frequency with which parents let the child play with paints, mud, or messy objects, let the child use objects to build towers or other creations, sing to the child, read the child stories, praise the child, kiss or cuddle the child, shout at the child, slap the child, go to a park or playground with the child, have a meal with the child, and let the child make a lot of noise), history of postnatal depression; head circumference measures in different periods were simultaneously included in the model.	Linear Regression (Betas): IQ total (4 years): Head circumference at birth – Crude: 2.14 (CI95%:1.02;3.26); Adjusted: 2.41 (CI95%:1.31;3.50); Head growth from birth to 1 year - Crude: 2.60 (CI 95%1.27;3.94); Adjusted: 1.97 (CI95%:0.68;3.26); from 1 to 4 years - Crude: 1.59 (CI95%: -0.20 ; 3.37); Adjusted: 0.46 (CI95%:-1.25;2.17); Total IQ (8 years): Head circumference at birth - Crude: 0.70 (CI95%: -0.57;1.98); Adjusted: 0.81(CI95%: -0.40;2.02); Head growht from birth to 1 year - Crude: 1.94 (CI95%:0.41–3.47); Adjusted: 1.56 (CI95%:0.11;3.01); from 1 to 4 years - Crude: 2.93 (CI95%: 0.90;4.97); Adjusted: 1.78 (IC95%: -0.15;3.71); from 4 to 8 years - Crude: -0.03 (CI95%: -2.43 ; 2.37); Adfjusted: -0.57 (CI95%:-2.98;1.72).
Short and Long-Term Effects of Compromised Birth Weight, Head Circumference, and Apgar Scores on	To demonstrate that small head circumference and low 5-minute Apgar scores are predictors for developmental	Data come from part of the Johns Hopkins Collaborative Perinatal Study, which included pregnant women receiving prenatal care at the perinatal clinic	Head circumference in centimeters, dicotomized into ≤ 32 cm and > 32 cm (reference group) [at birth].	Intelligence/Cognition: Bayley Scales of Mental development, dichotomized into advanced or normal, and suspect or abnormal [8 months]; Stanford-Binet	Socioeconomic level, maternal ethnicity, gestational age, sex.	Logistic Regression (Odds Ratio): Mental development suspect or abnormal (8 months): 3.6 (CI95%:2.5;5.0) p=0.0002; 4 years: 1.7 (CI 95%:1.3;2.2); Mean

Title, author, year, country, study-design	Objective	Study sample [N]	Exposure	Outcomes	Confounders	Main Results
Neuropsychological Development. **Gampel, 2014** United States Cohort	abnormalities throughout childhood and later.	delivered their babies at Johns Hopkins Hospital during 1960-1964. Twenty-five years later, between 1992 e 1994, those offspring were re-contacted (mean age 31) [N= 2151].		IQ standardized with a mean of 100 and a standard deviation of 15 [4 years]; Wechsler Intelligence Scale for Children (WISC) standardized with a mean of 100 and a standard deviation of 15 [7 years]. Academic performance: Wide Range Achievement Test (WRAT) - reading, arithmetic, and spelling, whose intervals varied between 0–76, 0–56 e 0–32, respectively [8 years].		(covariance analysis): IQ (4 years): Head circumference ≤ 32 cm 89.2; > 32 cm: 93.5 p<0.0001; IQ (7 years): Head circumference ≤ 32 cm: 89.4; > 32 cm: 92.6 p<.0001; WRAT (8 years) - Reeding: Head circumference ≤ 32 cm: 29.7; > 32 cm: 31.2 p=0.004; Spelling: Head circumference ≤ 32 cm: 21.7; > 32 cm: 22.5 p=0.003; Arithmetic: Head circumference ≤ 32 cm: 18.3; > 32 cm: 19.2 p<0.0001;
Newborn head size and neurological status. Predictors of growth and development of low birth weight infants. **Gross, 1978** United States Cohort	In a survey at age 4 to 5 years of children who were admitted to intensive care nurseries in Syracuse, NY, in 1971, two specific associations between neonatal status and outcome emerged that have not generally been reported. These factors were the presence of small head size at birth and the recognition of abnormal neurological behavior in the newborn period. Their association with later handicap	Infants with birth weights between 750 and 2000 g admitted to the neonatal intensive care units of the Crouse-Irving Memorial Hospital or the St Joseph's Hospital, Syracuse, New York, in 1971, who survived up to 5 years of age [N=118].	Head circumference percentile for estimated gestational age from standard charts, categorized into head circumference < 10th percentile, from 10 to 25th percentile, from 26 to 50th percentile, and >50th percentile) [at birth].	Intelligence/Cognition: The Goodenough-Harris drawing test was administered to all children and scored on the basis of 73 characteristics by two independent observers [4-5 years].	No adjustment was performed.	Mean IQ (T test): Head circumference < 10th percentile: 82.8 p<0.05; from 10th to 25th percentile: 91.9 p>0.05; from 26 to 50th percentile: 91.7 p>0.05; > 50th percentile: 100.6 p<0.05; IQ < 70 (Chi squared test): Head circumference < 10th percentile: 5/17 p<0.01; from 10 to 25th percentile: 3/47 p>0.05; from 26 to 50th percentile: 3/22 p>0.05; >50th percentile: 1/17 p>0.05; refefence group was not defined; aparentely,the comparion was performed with the complemental group.

Title, author, year, country, study-design	Objective	Study sample [N]	Exposure	Outcomes	Confounders	Main Results
	forms the basis of the present report.					
Intrauterine Growth Restriction, Head Size at Birth, and Outcome in Very Preterm Infants. **Guellec, 2015** France Cohort	To determine whether small head circumference or birth weight or both are associated with neonatal and long-term neurologic outcome in very preterm infants.	Data from the 1997 Epipage cohort study, which included all live births between 22 and 32 weeks of gestation in 1997 in 9 regions on France. Because 65% of those born at 22-25 weeks died before discharge, we limited our analysis to children born alive at 26-32 weeks [N=1315].	Head circumference percentile [at birth]. Infants were classified into: Symmetric Growth Restriction (SGR): head circumference and birth weight < 10th percentile or both between 10th and 19th percentile; Head Growth Restriction (HGR) - asymmetric: head circumference < 20th percentile and birth weight at least the next higher decile group; Appropriate for for Gestational Age (AIG): Both head circumference and birth weight > 20th percentile (reference group).	Intelligence/Cognition: Kauffman assessment battery for children, expressed as a mental processing composite score (IQ equivalent), standardized with a mean of 100 and a SD of 15 in a French population born in the 1990s, categorized into score<70, between 70 and 84, and >84 [5 years]. Academic Performance: School difficulties were assessed at age 8 years based on a parental questionnaire. Special schooling (institution, special school and special class in mainstream school, compared with mainstream class) or low grades were considered school difficulties [8 years].	Socioeconomic level, maternal age, parity, gestational age, sex, nacionality (only for intelligence), type of delivery (only for academic performance).	Logistic Regression (Odds Ratio): Score between 70 - 84: SGR - crude: 1.48 (CI95%:0.92;2.38); adjusted: 1.65 (CI95%1.01;2.71); HGR: crude: 1.10 (CI95%:0.66;1.84); adjusted: 1.20 (IC95%0.70;2.04); Score < 70: SGR: crude: 1.99 (CI95%:1.16;3.42); adjusted: 2.61 (CI95%:1.46;4.68); HGR: crude: 1.71 (CI95%:0.98;2.99); adjusted: 2.07 (IC95%:1.15;3.74); School difficulties: SGR: crude: 1.65 (CI95%:1.08;2.52); adjusted: 1.79 (IC95%:1.13;2.83); HGR: crude: 1.51 (IC95%:0.95;2.40); adjusted: 1.48 (IC95%:0.90;2.43).

Title, author, year, country, study-design	Objective	Study sample [N]	Exposure	Outcomes	Confounders	Main Results
Very low birth weight infants: effects of brain growth during infancy on intelligence quotient at 3 years of age. **Hack, 1986** United States Cohort	To test the general hypothesis that brain growth during the first year of life influences later intelligence in the very low birth weight infant and estimate its unique contribution to IQ at 3 years of age, controlling for the effects of other biologic and social factors.	Very low birth weight infants (<1500g) admitted to the Neonatal Intensive Care Unit, Rainbow Babies and Childrens Hospital, Cleveland, in 1977 and 1978 who were born appropriate for gestational age (birth weight for gestational age ≥ -2 SD) [N=139].	Head circumference z-score, dicotomized into head circumference <-2 SD and ≥-2 SD (reference group) [40 weeks, 8, 20 and 33 months corrected age].	Intelligence/Cognition: IQ measured by the Stanford-Binet intelligence test [33 months corrected age].	Socioeconomic level, neonatal risk score (Hobel's neonatal risk score), neurological impairment, race, weight	Mean IQ (Scheffe test): 40 weeks - Head circumference ≥-2 SD: 94.2; <-2 SD: 88.2 p>0.05; 8 months: ≥-2 SD: 95.0; <-2 SD: 79.3 p=0.0004; 20 meses: ≥-2 SD: 94.1; <-2 SD: 85.6 p=0.0055; 33 meses: ≥-2 SD: 94.1; <-2 SD: 77.3 p=0.05; Trajectory analysis (Beta – standardized) Head circumference at 8 months: -0.19 p<0.05
Differential effects of intrauterine and postnatal brain growth failure in infants of very low birth weight. **Hack, 1989** United States Cohort	To investigate the contribution of brain growth failure to outcome in infants of very low birth weight (<1.5 kg) who were appropriate for gestational age (AGA; n = 379) or small for gestational age (SGA; n=102).	Very low birth weight infants (<1500g) admitted to the Neonatal Intensive Care Unit, Rainbow Babies and Childrens Hospital, Cleveland, from January 1977 through December 1982 who survived to their second year. Among children with growth and/or developmental data available for the analysis (81%), 79% were born AGA and 21% were born SGA (< -2 SDs for age) [N=481].	Head circumference z-score according to age, dicotomized into head circumference <-2 SD and ≥-2 SD (reference group) [8 months]	Intelligence/Cognition: The Bayley Scales of Infant Development, Mental evelopmental index (MDI) [20 months].	Socioeconomic level, neonatal risk score (Hobel's neonatal risk score), neurological impairment, race	Linear Regression (betas)AIG: -9.987 p<0.05; PIG: -1.040 >0.05
Effect of very low birth weight and subnormal head size on cognitive abilities at school age. **Hack, 1991** United States Cohort	To extend our study of children with very low birth weights to eight years of age and test the hypothesis that children with subnormal head sizes at eight	Children with very low birth weight (<1,5 Kg) who were admitted to the neonatal intensive care unit at Rainbow Babies and Children's Hospital in Cleveland from January 1977 through December 1979	Head circumference z-score according to age, dicotomized into head circumference <-2 SD and ≥-2 SD (reference group) [8 months]	Intelligence/Cognition: Wechsler Intelligence Scale for Children (WISC-R) - verbal IQ and Performance IQ), continuous and categorized into IQ <70, between 70-84 and > 84 (reference group).	Maternal socioeconomic disadvantage (composite of marital status, race, and level of education), neonatal risk score, intrauterine growth,	Crude analysis: T test (Mean difference): Intelligence – Verbal IQ: 13.4 (CI95%:6.98,19.76); Performance IQ: 13.8(CI95%:7.44,20.20); Academic Performance - Reading: 16.5 (CI95%:9.38,23.59);

Title, author, year, country, study-design	Objective	Study sample [N]	Exposure	Outcomes	Confounders	Main Results
	months of age have significantly poorer outcomes at school age than children with normal head sizes by the age of eight months.	who survived up to 8 years of age [N=249].		Academic achievement: Reading composite - Woodcock Reading Mastery Test (word attack, word identification, and passage comprehension); Mathematics cluster - Woodcock-Johnson Psychoeducational Battery: calculation and applied problems; Spelling - Wide-Range Achievement Test. Limited academic skills was defined as reading, mathematic or spelling stand score < 80 [8-9 years].	birth weight and neurologic status.	Mathematics: 8.2 (CI95%:2.44;13.90); Spelling: 12.0 (CI95%:5.04;18.87); Qui-squared test (Relative Risk): Total IQ < 70: 7.48(CI95%:2.90,19.26); Total IQ < 85: 1.91(CI95%:1.28,2.85); Limited academic skills: 1.81(CI95%:1.25,2.61); Adjusted analysis: Linear Regression (Beta): Intelligence - Verbal IQ: -7.78 p=0.011; Performance IQ: -7.70 p=0.007; Academic Performance - Reading: -11.20 p=0.001; Mathematics: -4.41 p=0.102; Spelling: -7.62 p=0.026.
Functional principal component analysis for identifying multivariate patterns and archetypes of growth, and their association with long-term cognitive development. **Han, 2018** Republic of Belarus Cohort	To demonstrate a data analysis procedure that combines measurements from three commonly recorded time-varying growth traits, head circumference, body length and body weight. We then identify growth patterns that can be associated with subsequently measured full-scale IQ (Wechsler abbreviated scale of intelligence, WASI). The simultaneous	Data from WHO's Promotion of Breastfeeding Intervention Trial (PROBIT) in the Republic of Belarus, developed between June 1996 and December 1997. Mothers were considered eligible for participation if they expressed an intention to breastfeed on admission to the postpartum ward, had no illnesses that would contraindicate breastfeeding or severely compromise its success, and had given birth to a healthy singleton infant of 37	Head circumference [1, 2, 3, 6, 9 and 12 months]; Four groups of head growht pattern were identified: Generally Large (Always above the mean of head circumfence), Catch-up (Below the mean initially, but above the mean in the end of follow-up) Stunting (Always below the means), Faltering (Above the mean initially, but below the mean in the end	Intelligence/Cognition: Wechsler abbreviated scale of intelligence (WASI Full-scale IQ) [6.5 years].	Maternal and paternal age, maternal and paternal schooling, smoking during pregnancy, sex, breatfeeding, hospital where child was born.	Functional principal component analysis, following by ANOVA/ Kruskal Wallis, linear mixed model, and post-hoc analysis with Turkey's multiple comparison procedure (Mean difference – shown in a chart). In text: "Stunting" and "Faltering" were associated with higher risk in comparison with the "Generally Large" and "Catch-up" subgroups. The results were suggestive of higher risk for "Faltering" versus "Catch-up" (p-value = 0.060 after Bonferroni correction). From the post-hoc analysis, "Generally Large" and "Stunting"

Title, author, year, country, study-design	Objective	Study sample [N]	Exposure	Outcomes	Confounders	Main Results
	consideration of multiple trajectories is a main novel feature of our approach.	weeks' or more gestation, 2500 g or more birth weight, and Apgar score 5 or higher at 5 minutes. Sites were randomly assigned to receive an experimental intervention modeled on the Baby-Friendly Hospital Initiative for maintaining breastfeeding and lactation and postnatal breastfeeding support, or a control intervention of continuing usual infant feeding practices and policies [N=12809].	of follow-up), "regular" (infants that did not fill criteria for outliers groups above described).			subgroups had significantly different IQ performance for head circumference (p-value = 0.002), but no significant difference was found between other subgroups such as "Catch-up" or "Faltering".
Physical growth and nonverbal intelligence: associations in Zambia. **Hein, 2014** Zambia Cross-sectional	To investigate normative developmental body mass index trajectories and associations of physical growth indicators—height, weight, head circumference , and BMI—with nonverbal intelligence in an understudied population of children from sub-Saharan Africa.	Data from Bala Bbala Project, a large-scale Institutional Review Board–approved study of the manifestation, prevalence, and etiology of specific reading disabilities in rural Zambia. After screening and enrollment, the following children were excluded from this analysis: those with missing data for BMI, intelligence, or head circumference; those age >19 years; and those with vision poorer than 20/30 in both eyes or hearing loss of >40 dB for at least 1 of the assessed frequencies in both ears [N=3981].	Head circumference [7-8 years].	Intelligence/Cognition: The Universal Nonverbal Intelligence Test, Symbolic Memory subtest (UNIT-SM) and Kaufman Assessment Battery for Children, Second Edition, Triangles subtest (KABC-II-T) were administered to assess nonverbal intelligence (ie, memory and simultaneous visual processing, respectively). For comparison, scaled scores (possible range, 1-19) were derived from published US norms for the UNIT-SM and KABC-II- T [7-18 years].	Sex, grade (age was not used in order to avoid multicolinearity)	Linear Regression (Beta): 0.13 (CI95% presented, however inconsistent) p<0.001<br>Pearson Correlation Coefficient: 0.17 p<0.05

Title, author, year, country, study-design	Objective	Study sample [N]	Exposure	Outcomes	Confounders	Main Results
Prenatal and postnatal growth and cognitive abilities at 56 months of age: A longitudinal study of infants born at term **Heinonen, 2008** Finland Cohort	To investigate whether weight, length, BMI (kilograms per meter squared), and head circumference at birth and their postnatal growth are associated with cognitive abilities at 56 months of age among infants born at term.	The study cohort was composed of infants participating in the Arvo Ylppo Longitudinal Study, which included infants born at term (>37 weeks or <42 weeks in the 7 maternity hospitals in the county of Uusimaa, Finland, between March 15, 1985, and March 14, 1986. Infants with major impairments and/or mental retardation (ie, major congenital malformations, chromosomal ab-normalities, grade 2–4 cerebral palsy, deafness or a hearing defect requiring a hearing aid, epilepsy, and/or with developmental delay at 56 months of age) were excluded [N= 1056].	Head circumference z-score according to sex; The growth variables were the standardized residuals from the linear regression models of body size [at birth, 5, 20 and 56 months]	Intelligence/Cognition: General reasoning, measured on the Columbia Mental Maturity Scale, a nonverbal cognitive-ability test consisting of 100 cards displaying sets of 3 to 5 drawings from which the child has to select the 1 that is different from or unrelated to the others. All of the cognitive test scores were corrected for exact age at measurement and standardized (mean: 100; SD: 15) [56 months].	Sex, gestational age (Model I) + admission in neonatal ward, breastfeeding, smoking during pregnancy, multiple birth, parental schooling, maternal age at delivery and maternal height (Model II)	Linear Regression (Betas): at birth - Model I: 0.83 (CI95%:0.24;1.43) p<0.01; Model II: 0.78 (CI95%:0.17;1.40) p<0.05; from birth to 5 months - Model I: 0.61 (CI95%:-0.24; 1.46) p≥0.05; Model II: 0.66 (CI95%:-0.18;1.49) p≥0.05; from 5 to 20 months - Model I: 0.73 (CI95%:-0.14;1.60) p≥0.05; Model II: 0.56(CI95%:-0.29;1.41) p≥0.05; from 20 to 56 meses - Model I: -0.01(CI95%:-0.83;0.82) p≥0.05; Model II: -0.14 (CI95%:-0.96;0.67) p≥0.05;
Extreme prematurity, growth and neurodevelopment at 8 years: a cohort study. **Hickey, 2021** Australia Cohort	(1) To compare growth from birth to 2 years and 2 to 8 years, and (2) to investigate the associations of growth with cognitive, academic, executive and motor function at 8 years, across three eras from 1991 to 1992, 1997 and 2005.	All extremely preterm babies (22–27 completed weeks' gestation) survivors in the state of Victoria were recruited in three distinct eras: 1991–1992 (24 months), 1997 (12 months) and 2005 (12 months). Controls comprised infants born with gestational ages ≥37 weeks or a birth weight ≥2500g, matched for social variables and were	Head circumference-z scores relative to the British Growth Reference data, which adjusts measurements for age and sex. Growth was calculated between birth and 2 years, and between 2 and 8 years as the z-score difference	Intelligence/Cognition: Wechsler Intelligence Scales for Children, third edition (1991-1992) and fourth edition (1997); Differential Ability Scales, second edition (2005) Academic performance - reading, spelling and arithmetic: the Wide Range Achievements Test, version 318 (1991–1992 and 1997), and version 419 (2005). Both cognitive and academic results were	Maternal schooling, social class, an interaction term for era and growth	Linear Regression (Betas): Head growth from birth to 2 years: IQ - crude: 0.44(CI95%:0.26;0.61); adjusted:0.39 (CI95%:0.21;0.57); Reading - crude: 0.28(CI95%:0.07;0.49); adjusted: 0.28 (CI95%:0.08;0.48); Spelling - crude: 0.16(CI95%:−0.03;0.35); adjusted: 0.16 (CI95%:−0.03;0.34); Arithmetic - crude:

Title, author, year, country, study-design	Objective	Study sample [N]	Exposure	Outcomes	Confounders	Main Results
		recruited for standardization of test scores at 8 years for some outcome variables [N=499].	over that time for individuals with data points at both ages. [at birth, 2 and 8 years].	converted to z-scores relative to the mean scores of contemporaneous controls within each era [8 years corrected age].		0.15(IC95%:−0.06;0.37); ajustado: 0.14 (IC95%:−0.07;0.35); Head growth from 2 to 8 years: IQ - bruto: −0.08(CI95%:−0.83;0.67); adjusted: −0.23 (CI95%:−0.98;0.51); Reading - crude: −0.05(CI95%:−0.71;0.61); adjusted: −0.14 (CI95%:−0.77;0.49); Spelling - crude: 0.04(CI95%:−0.59;0.67); adjusted: 0.02 (CI95%:−0.64;0.59); Arithmetic - crude: 0.14 (CI95%:−0.65;0.93); adjusted: 0.09 (CI95%:−0.71;0.89). (No evidence of interaction by era: data was combined for analysis).
Cognition and behavioural development in early childhood: the role of birth weight and postnatal growth. **Huang, 2013** China Cross-sectional	To explore the relative importance of birth weight and postnatal growth in early childhood for a broad measure of cognitive and behavioural development, using a large sample of Chinese children aged 4–7 years. To investigate whether the findings were different between preterm children and term children.	Data were from a China–US collaborative project designed to prevent neural tube birth defects with periconceptual folic acid supplementation during the period 1993–1996. The program enrolled women who were preparing for marriage from Hebei, Zhejiang and Jiangsu provinces in China and tracked the pregnancy/birth outcomes by the maternal and child health hospital- based	Head circumference in centimeters [4-7 years].	Intelligence/Cognition: Chinese Wechsler Young Children Scale of Intelligence (C-WYCSI), which included five subsets (knowledge, arithmetic comprehension, picture completion mazes, block design and geometric design, picture vocabulary, picture summary and animal peg). The results were standardized for age [4-7 years].	Maternal height, maternal weight, maternal IQ, maternal schooling, parity, weight, gestational age, mother taking folic acid supplement during pregnancy, father's occupation, urban residency.	Two-Stage Least-Squares Regression (betas)Preterm: Adjusted for sex 1.10 (CI95%:0.31; 1.89); Full adjustment: 0.30 (CI95%:-0.42; 1.02); Term: Adjusted for sex: 1.49 (CI95%:1.22; 1.76) ; Full adjustment: 1.05 (CI95%:0.80; 1.29)

Title, author, year, country, study-design	Objective	Study sample [N]	Exposure	Outcomes	Confounders	Main Results
		monitoring system. In 2001, a random sample of 9100 children aged 4–7 years was selected from the children born to these women. Multiple pregnancies were excluded [N= 8389].				
Brain development parameters and intelligence in Chilean high school graduates **Ivanovic, 2004** Chile Cross-sectional	To describe some brain development parameters in Chilean high school graduates of both sexes from high and low socioeconomic stratum and to confirm the hypothesis that independently of sex and socioeconomic stratum, brain volume and head circumference are positively and significantly associated with intellectual quotient.	Right-handed high school graduate students (mean age 18 years) born at term who attended public and private schools in the richest and the poorest counties of the Chile's metropolitan region applying the UNICEF classification. Adolescents who presented history of alcoholism or antecedents or symptoms of brain damage, intrapartum fetal asphyxia, hyperbilirubinemia, epilepsy, or heart disease and their mother had no history of smoking, alcoholism, and drug intake before and during pregnancy were excluded. Two groups of high school graduates were formed and compared: Group 1, high IQ (>120); and Group 2, low IQ (<100). The same proportion of school-age children according to	Head circumference in centimeters and z-score, adjusted for sex, weight and height [mean 18 years].	Intelligence cognition: Wechsler Intelligence Scale for Adults - Revised (WAIS-R) adapted for Chilean population. Group 1, high IQ (>120 WAIS-R); and Group 2, low IQ (<100 WAIS-R) [mean 18 years].	No adjustment was performed.	T Test: Head circumference (cm): Men: High IQ: mean 56.5 (SD 1.4); Low IQ: 54.8 (1.6) p< 0.01; Women: High IQ: 54.8 (1.2); Low IQ: 54.0 (1.2) p < 0.05; T Test: Head circumference (Z-score): Men: High IQ: 0.57 (1.06); Low IQ -0.78(1.22) p< 0.01; Women: High IQ: -0.01 (0.84); Low IQ: -0.63 (0.95) p < 0.05 Pearson Correlation: Head circumference and IQ total: Men 0.499 (p<0.001); Women 0.397 (p<0.01)

Title, author, year, country, study-design	Objective	Study sample [N]	Exposure	Outcomes	Confounders	Main Results
		socioeconomic level (high and low) (1:1) and sex (1:1) were included in each IQ group [N=96].				
Head size and intelligence, learning, nutritional status and brain development. Head, IQ, learning, nutrition and brain. **Ivanovic, 2004** Chile Cross-sectional	To determine the interrelationships between the head size and intelligence, learning, nutritional status, brain development and parental head size in healthy Chilean school-age children graduating from high school, of both sexes, with high and low intellectual quotient and of the high and low socio-economic strata.	Right-handed high school graduate students (mean age 18 years) born at term who attended public and private schools in the richest and the poorest counties of the Chile's metropolitan region applying the UNICEF classification. Adolescents who presented history of alcoholism or antecedents or symptoms of brain damage, intrapartum fetal asphyxia, hyperbilirubinemia, epilepsy, or heart disease and their mother had no history of smoking, alcoholism, and drug intake before and during pregnancy were excluded. Two groups of high school graduates were formed and compared: Group 1, high IQ (>120); and Group 2, low IQ (<100). The same proportion of school-age children according to socioeconomic level (high and low) (1:1) and sex (1:1) were included	Head circumference z-score categorised into <−2, −2 to <0, 0–2 and >2 SD [mean 18 years].	Intelligence cognition: Wechsler Intelligence Scale for Adults - Revised (WAIS-R) adapted for Chilean population. Group 1, high IQ (>120 WAIS-R); and Group 2, low IQ (<100 WAIS-R) [mean 18 years]. Academic performance: School Achievement was evaluated through standard Spanish language and mathematics tests especially designed for the study. Results were expressed as percentage of achievement in overall results, as well as in Spanish language and mathematics; Academic aptitude test (AAT), the baccalaureate examination for university admission with national coverage, which included both verbal (90 items) and mathematics (60 items) tests, with a maximum score of 900 in each test. The overall results in the AAT were also calculated [mean 18 years].	No adjustment was performed.	Mean (ANOVA): Total IQ: Head circumference <−2 SD: 91.0; −2 to <0 SD: 104.3; 0 to 2 SD: 115.4; > 2 SD: 124.0 $p< 0.01$; Verbal IQ: <−2 SD: 87.8; −2 to <0 SD: 104.2; 0 to 2 SD: 114.8; > 2 SD: 128.7 $p< 0.001$; Non-verbal IQ: <−2 SD: 96.4; −2 to <0 SD: 104.2; 0 to 2 SD: 113.9; > 2 SD: 114.3 $p< 0.05$; School Achievement: <−2 SD: 8.6; −2 to <0 SD: 46.6; 0 to 2 SD: 56.6; > 2 SD: 78.8 $p< 0.01$; Academic aptitude test: <−2 SD: 403.3; −2 to <0 DP: 545.0; 0 to 2 SD: 643.7; > 2 SD: 744.8 $p< 0.01$; Pearson Correlation: IQ total: 0.465 $p< 0.0001$; School Achievement: 0.450 $p< 0.0001$; Academic aptitude test 0.434 $p< 0.0001$.

Title, author, year, country, study-design	Objective	Study sample [N]	Exposure	Outcomes	Confounders	Main Results
		in each IQ group [N=96].				
Scholastic achievement: a multivariate analysis of nutritional, intellectual, socioeconomic, sociocultural, familial, and demographic variables in Chilean school-age children. **Ivanovic, 2004** Chile Cross-sectional	To quantitate the relative effects of nutritional, intellectual, socioeconomic, sociocultural, familial, and demographic variables on scholastic achievement of children in elementary and high schools from Chile's Metropolitan Region.	School-age children enrolled in grades 1, 2, 4, 6, and 8 of elementary schools and in grades 1 and 4 of high schools in the Metropolitan Region of Chile in 1986, who attended public, privately subsidized, and private non-subsidized schools in urban and rural areas. The survey was conducted in 13 schools in eight counties of the Metropolitan Region of Chile [N= 4509].	Head circumference z - score [mean 10.4 years].	School Achievement was evaluated through standard Spanish-language achievement and mathematic achievement tests designed especially for this study. The number of items varied with each grade (elementary: grade 1, 16; grade 2 ,16; grade 4, 40; grade 6, 50; grade 8, 62; high school: grade 1, 70) and was similar for spanish-language and mathematic (1:1); for high school grade 4, the Academic Aptitude Test score was considered (150 items with a maximum score of 900). Results were expressed as percentage of achievement in overall results and in spanish-language and mathematic [mean 10.4 years].	Maternal schooling, paternal schooling, height, intellectual ability, book reading; sewage; type of school, quality of housing, calcium intake.	Linear Regression(Beta): 1.15 p<0.001; Pearson Correlation: 0.220 p < 0.0001
Neuropsychological Parameters Affecting the Academic Aptitude Test (AAT) Achievement at the End of High School in 1996 and Their Impact on Job Status in 2002: A Multifactorial	To investigate the impact of neuropsychological parameters in a multicausal context, on the Academic Aptitude Test achievement of Chilean high school graduates in 1996 with high or low intellectual quotient	Right-handed high school graduate students (mean age 18 years) born at term who attended public and private schools in the richest and the poorest counties of the Chile's metropolitan region applying the UNICEF classification. Adolescents who	Head circumference z-score [mean 18 years].	Academic performance: Academic aptitude test (AAT), the baccalaureate examination for university admission with national coverage, with a maximum score of 900 in each test for the overall results [mean 18 years]. Employment: Student's job status 2002 was determined after six years	No adjustment was performed.	Mean – head circumference z-score (T test): Boys: High AAT: 0.56; Low AAT: -0.38 p<0.01; Girls: High AAT: 0.02; Low AAT: -0.48 p>0.05; Student's job status: jobless: -1,14; workers without further schooling: -1,19; students at technical institutes: -0,37; students at

Title, author, year, country, study-design	Objective	Study sample [N]	Exposure	Outcomes	Confounders	Main Results
Approach in a Follow-up Study. **Ivanovic, 2006** Chile Cohort	and socio-economic status and on job status carried out six years later during 2002.	presented history of alcoholism or antecedents or symptoms of brain damage, intrapartum fetal asphyxia, hyperbilirubinemia, epilepsy, or heart disease and their mother had no history of smoking, alcoholism, and drug intake before and during pregnancy were excluded. Two groups of high school graduates were formed and compared: Group 1, high IQ (>120); and Group 2, low IQ (<100). The same proportion of school-age children according to socioeconomic level (high and low) (1:1) and sex (1:1) were included in each IQ group [N=96].		of high school graduation and was expressed as jobless, workers without further schooling, students at technical institutes, students at universities [around 24 years].		universities: 0,48 p< 0.01; Pearson Correlation: Total sample: 0.434 p < 0.0001; Boys: 0.475 p< 0.01; Girls: 0.393 p< 0.01;
Twelve-year follow-up study of the impact of nutritional status at the onset of elementary school on later educational situation of Chilean school-age children. **Ivanovic, 2008** Chile Cohort	To determine the impact of nutritional status in a multicausal approach of socio-economic, socio-cultural, family, intellectual, educational and demographic variables at the onset of elementary school in 1987 on the educational situation of these children in 1998,	School-age children who were enrolled in first grade of elementary school in the Metropolitan Region of Chile in 1987 [N= 813].	Head circumference z-score adjusted for age, sex and body size and categorised into <−2, −2 to 2 and >2 SD [5.5–9 anos].	Educational attainment: educational situation in 1998 classified as graduated, delayed, dropout (children who leaved school without returning to it) and not located [in 1998, when adolescents should have graduated in high school].	Socioeconomic level, intellectual ability, scholastic achievement	Mean – Head circumference z-score (Scheffe's test): not located: 0.14; dropout: -0.50; delayed 0.15; graduated: 0.45 p<0.0001; Logistic regression (Odds Ratio): dropout vs non-dropout: 2.317(CI95%:1.196;4.490) p<0.05; dropout vs delayed: 2.130 (CI 95%:1.049;4.325); p<0.05; dropout vs graduated: 2.766 (CI 95%:1.349;5.671) p<0.01

Title, author, year, country, study-design	Objective	Study sample [N]	Exposure	Outcomes	Confounders	Main Results
	when they should have graduated from high school.					
Impact of nutritional status at the onset of elementary school on academic aptitude test achievement at the end of high school in a multicausal approach. **Ivanovic, 2009** Chile Cohort	To describe the impact of nutritional, intellectual, family, educational and socio-economic variables at the onset of elementary school in 1987 that may affect achievement on the academic aptitude test (AAT) taken in 1998 at the end of high school, and to quantify the impact of these independent variables on the AAT.	School-age children enrolled in the first grade of elementary schools in the Metropolitan Region of Chile in 1987, belonged to public (state), private-subsidised and private non-subsidised schools from both urban and rural areas from the Metropolitan Region of Chile, who graduated from high-school in 1998 and took the Academic Aptitude Test for university admission in the same year [N=260].	Head circumference z-score [mean 6.5 years].	Main outcome: Academic performance - Academic aptitude test (AAT), which included both verbal and mathematics tests, with a maximum score of 900 in each test [mean 18 years]. Additional outcomes (included as confounder in the regression model) - Academic performance: Scholastic achievement test, determined through Spanish language and mathematics tests, designed taking into consideration the objectives pursued by the curricular programmes of Ministry of Education in 1987. Intelligence/Cognition: Intellectual ability (IA) assessed with Raven's Progressive Matrices Test in book form, with a special scale for children aged 4 to 11 years [mean 6.5 years].	Maternal schooling, School achievement in 1987, intellectual ability in 1987	Linear Regression (Beta) - AAT: 13.4 p=0.0118; Pearson Correlation: Head circumference z-score and AAT: 0.239 p<0.0001; Head circumference z-score and Scholastic achievement: 0.238 p<0.0001; Head circumference z-score and Intelectual Abitity: 0.172 p<0.0001.
Brain development and scholastic achievement in the Education Quality Measurement System tests in Chilean school-aged children.	To determine the impact of the nutritional background and parameters of current nutritional status on language and mathematics	School-aged children enrolled in the fifth elementary school grade and in the first grade of high school in Chile's Metropolitan Region in 2010 and who took the SIMCE test at the end	Head circumference z-score dichotomized into < median or ≥ median (reference group) [5ESG: mean 10.8 years,	Academic performance: assessed through the SIMCE tests, which has national coverage in Chile and is administered by the Ministry of Education. Scores range between 0 and 400, are expressed as	Sex	Logistic Regression (Odds Ratio)5ESG: Language: 1.510 (CI95%:1.094; 2.085); Math: 2.447 (CI95%:1.753; 3.415) p< 0.0001;1HSG: Language: 2.030 (CI95%:1.466; 2.813) p< 0.0001; Math:

Title, author, year, country, study-design	Objective	Study sample [N]	Exposure	Outcomes	Confounders	Main Results
**Ivanovic, 2014**<br>Chile<br>Cross-sectional	SA in the Education Quality Measurement System (SIMCE) tests administered by the Ministry of Education.	of the year 2009. They belonged to public, private subsidized, and private nonsubsidized schools from urban areas [N=1353].	1HSG: mean 14.8 years].	scores (mean ± SD) in the language and mathematics tests, and for this study was dichotomized into two groups: <median and ≥median (reference group) [5ESG: mean 10.8 anos; 1HSG: mean 14.8 anos].		2.097 (CI95%:1.463; 3.006) p< 0.0001
A multifactorial approach of nutritional, intellectual, brain development, cardiovascular risk, socio-economic, demographic and educational variables affecting the scholastic achievement in Chilean students: An eight- year follow-up study.<br>**Ivanovic, 2019**<br>Chile<br>Cross-sectional	To quantitate the relative impact of nutritional, intellectual, brain development, cardiovascular risk, socio-economic, demographic and educational variables on the results of the 2009 SIMCE tests of language and mathematics.	School-aged children enrolled in the fifth elementary school grade and in the first grade of high school in Chile's Metropolitan Region in 2010 and who took the SIMCE test at the end of the year 2009. They belonged to public, private subsidized, and private nonsubsidized schools from urban areas [N=1353].	Head circumference z-score for age, dichotomized into < 0 or ≥ 0 (reference group) [5ESG: mean 10.8 years, 1HSG: mean 14.8 years].	Academic Performance: assessed through the 2009 SIMCE tests, which has national coverage and is administered by the Agency for Education Quality. Scores range between 0 and 400; results are expressed as mean ± SD and also categorized in three ranges of achievements: high, medium and low in relation to the age and educational establishments [5ESG: mean 10.8 years, 1HSG: mean 14.8 years].	Maternal schooling, Paternal schooling, schoolastic achievement in the respective educational establishment, Intellectual ability (Raven's Progressive Matrices Test)	Pearson Correlation: 5ESG - Language: 0.159 p=0.0002; 5ESG - Math: 0.242 p=0.0001; 1HSG - Language: 0.173 p=0.0001; 1HSG - Math: 0.330 p=0.0001<br>Mean difference: 5ESG - Language: 8.813 (CI95%: 0.803;16.824), p=0.031; 5ESG - Math: 15.034 (CI95%:7.513;22.554), p= 0.0001
Impact of anthropometric nutritional parameters on the university selection test in Chile: A multifactorial approach.<br>**Ivanovic, 2019**<br>Chile<br>Cohort	To quantitate the relative impact of nutritional, intellectual, brain development, cardiovascular risk, socio-economic, demographic and educational variables of school-age children at the onset of high school during 2010, on the 2013 University Selection Test outcomes, both in language and	School-aged children enrolled in the fifth elementary school grade and in the first grade of high school in Chile's Metropolitan Region in 2010 who took the SIMCE (Sistema de Medición de la Calidad de la Educación) test at the end of the year 2009, graduated from high school in 2013, and took the PSU, the baccalaureate examination with national coverage for	Head circumference z-score for age, dichotomized into < 0 or ≥ 0 (reference group) [1HSG: mean 14.8 years].	Academic performance: Results from the 2013 University selection test in both Spanish-language and mathematic achievement tests, expressed as mean and standard deviation, and categorized into low scholastic achievement (score <450), medium scholastic achievement (450–620), and high scholastic achievement (>620) [mean 18 years].	Maternal schooling, sex, schoolastic achievement in the respective educational establishment, Intellectual ability (Raven's Progressive Matrices Test)	Linear Regression (Betas): Language: -17.6 p=0.0323; Math: -22.2 p=0.0062; Pearson Correlation: Language: 0.234 p<0.0001; Math: 0.322 p<0.0001;<br>In text: Head circumference z-score values were significantly higher in school-aged children from the high language or mathematic achievement groups compared with their peers from the medium and low groups in the total sample

Title, author, year, country, study-design	Objective	Study sample [N]	Exposure	Outcomes	Confounders	Main Results
	mathematics tests, at the end of high school, for university admission, applying a multicausal approach.	admission to university, in 2013 [N=671].				(p< 0.0001 and p< 0.0001, respectively) and in male (p< 0.05 and p< 0.05, respectively) and female (p< 0.05 and p< 0.01, respectively) students.
Head Growth and Intelligence from Birth to Adulthood in Very Preterm and Term Born Individuals. **Jaekel, 2019** Germany Cohort	To investigate very preterm/very low birth weight and term born individuals' head growth from birth to 4 years and intelligence in childhood and adulthood, and to determine the specific timing of head growth that matters for intelligence development.	Data from Bavarian Longitudinal Study, which included very preterm (<32 weeks) and/or very low birth weight (<1500g) infants and an equally sized group of healthy term comparisons born in a geographically defined area of South Bavaria (Germany) between January 1985 and March 1986 [N=401].	Head circumfernce in centimeters and head growht within 2 consecutive measures, adjusted for gestational age [at birth, 20 months and 4 years].	Intelligence/Cognition: Griffiths' Mental Development Scales Developmental Quotient (DQ) items [20 months]; Kaufman Assessment Battery for Children (K-ABC) [6 and 8 years]; Age-normed Full-Scale IQ - Wechsler Adult Intelligence Scale (WAIS III) [26 years]	Socioecononomic level (assessed as a weighted composite score of parents' education and occupation), Igestacional age, birth weight.	Structural Equation Modeling (Betas) - Model 1: VP/VLBW and term born individuals – Total effect: Head circumference at birth and IQ at 6 years: 0.51; IQ at 8 years: 0.43; IQ at 26 years: 0.48; Head growth from birth to 20 months: IQ at 6 years: 0.18; IQ at 8 years: 0.15; IQ at 26 years: 0.25; Head growth from 20 months to 4 years: IQ at 6 years: 0.15; IQ at 8 years: 0.13; IQ at 26 years: 0.11; The effects of gestation and birth weight on intelligence were fully mediated by head circumference and growth while family SES directly predicted intelligence.
Cognitive ability in adolescents born small for gestational age: Associations with fetal growth velocity, head circumference and postnatal growth. **Jensen, 2015** Denmark Cohort	To examine the associations between early pre- and postnatal growth including measurements of fetal growth and intelligence in adolescence.	The baseline data were collected in 1985–1987 on pregnant women who had one or more risks factors for giving birth to an SGA child (i.e. smoking in pregnancy, previous birth to a SGA child, previous pre-eclampsia). Furthermore, a healthy control group was	Head circumference in centimeters [at birth, 3 months and 16-18 years]. (	Intelligence/Cognition: Wechsler Adult Intelligence Scale (WAIS), which included the six verbal and five performance subtests of the original. The raw scores were converted to scaled scores and the sums of scaled scores were converted to IQ using a normal reference population of 200 men and	Sex and adolescent (Model 1) + parental socioeconomic level, smoking in pregnancy, gestational age, birth weight; fetal growth velocity (Model 2)	Model 1 – head circumference at birth: Full-scale IQ: 2.16 p = 0.09; Model 1 - head circumference at 3 months: Full-scale IQ: 3.01 p = 0.01; Model 1 - head circumference at adolescence: Full-scale IQ: 1.30 p = 0.01; verbal IQ: 1.46 p = 0.008; performance IQ: 0.89 p = 0.09 Model 2 - head

Title, author, year, country, study-design	Objective	Study sample [N]	Exposure	Outcomes	Confounders	Main Results
		selected from a birth registry of children born at the same hospital in the same time period who had a birth weight between the 25th and the 75th percentile and no maternal risk factors. Only term-born children were invited to participate [N=144].		women with a mean age of 23 years [16-18 years].		circumference at adolescence: Full-scale IQ: 1.34 p = 0.05.
The association of growth impairment with neurodevelopmental outcome at eight years of age in very preterm children. **Kan, 2008** Australia Cohort	To determine the associations between weight and head circumference, at birth and postnatally, with cognitive, academic and motor outcomes at age 8 years for very preterm children free of neurosensory impairment.	Very preterm infants (23–27 weeks' gestational age) born in 1991 and 1992 in the state of Victoria, Australia, who survived to the age of 8 years.Children with any cerebral palsy, blindness or deafness were excluded [N=179].	Head circumference z-scores - To ascertain the postnatal growth over any time period, the Z-score at the start of the period was subtracted from the Z-score at the end of the period. Catch-up growth in any time period comprised a positive value for change in a postnatal growth Z-score [at birth, 2 and 8 years].	Intelligence/Cognition: Wechsler Intelligence Scale for Children -Third Edition (WISC-III); which yields a standard score with a mean of 100 and SD of 15. A control group of normal birth weight (birth weight N > 2499 g) and term (gestational age N > 36 weeks) children was recruited to enable standardisation of the IQ test scores; Academic Performance: Wide Range Achievement Test, 3rd edition (WRAT3), which included three subscales that assess reading (word recognition and decoding), spelling and arithmetic and yields a standard score with a mean of 100 and SD of 15 [8 years].	Maternal schooling, intraventricular haemorrhage, cystic periventricular leucomalacia, surgery and postnatal corticosteroids, language other than English spoken at home	Linear Regression (Betas): IQ - Head circumfence at birth: -0.005 (CI 95%: −0.180; 0.171) p=0.96; 2 years: 0.224 (CI 95%:0.088;0.360) p=0.001; from birth to 2 years: 0.141 (CI 95%: 0.023; 0.259) p=0.019; 8 years: 0.146 (CI 95%: 0.008; 0.284) p=0.038; from 2 to 8 years: − 0.152 (CI 95%: − 0.376; 0.072) p=0.18; Reading: Head circumference at birth: 0.88 (CI95%:− 1.86; 3.61); 2 years: 2.88 (IC95%:0.90; 4.86); from birth to 2 years:1.96 (CI95%:0.18; 3.74); 8 years: 2.80 (CI95%:0.85; 4.75); from 2 to 8 years: 0.95 (CI95%:− 2.39; 4.30); Spelling: Ar birth: 0.17 (CI95%:− 1.94; 2.25); 2 years: 1.99 (CI95%0.45; 3.53); from birth to 2 years: 1.37 (CI95%:0.03;2.71); 8 years: 1.89 (CI95%:0.35; 3,42); from 2 to 8 years: 0.17 (CI95%: − 2.43; 2.77); Arithmetic: At birth:

Title, author, year, country, study-design	Objective	Study sample [N]	Exposure	Outcomes	Confounders	Main Results
						0.92 (CI95%:−1.42;3.26); 2 years: 1.81 (CI95%:−0.02; 3.65); from birth to 2 years:1.10 (CI95%: −0.40, 2.60); 8 years: 1.41 (CI95%: −0.36;3.12); from 2 to 8 years: −0.79 (IC95%: −3.75; 2.17)
Cognitive Outcomes of Children with Very Low Birth Weight at 3 to 5 Years of Age.<br>**Kim, 2020**<br>South Korea<br>Cohort	To determine the influence of perinatal and neonatal risk factors on the cognitive performance of very low birth weight children at 3 to 5 years of age.	Infants with a birth weight of < 1500 g admitted to the NICU at Seoul Hanyang University Hospital between January 2010 and November 2012. Children with genetic syndromes, congenital malformations, chromosomal anomalies and cystic periventricular leukomalacia were excluded [N=88].	Head circumference in centimeters [at birth, 6, 12 and 18 months, 3-5 years].	Intelligence/Cognition: Korean version of the Wechsler Preschool and Primary Scale of Intelligence IV (WPPSI-IV), including subscales of vocabulary comprehension intelligence quotient (IQ), visual-spatial ability IQ, and working memory. The results were standardized to mean 100, and dichotomized into total IQ < 85 and ≥ 85 (reference group) [3-5 years].	No adjustment was performed.	Mann-Whitney U test<br>Mean - Head cirucmference - at birth: IQ < 85: 34.4; IQ ≥ 85: 34.6 p=0.847; 6 months - IQ < 85: 40.4; IQ: ≥ 85: 41.4 p≥0.05; 12 months - IQ < 85: 43.8; IQ ≥85: 44.3 p≥0.05; 18 months - IQ <85: 45.3; IQ ≥ 85: 46.5 p≥0.05; 3-5 yeas - IQ <85: 51.7; IQ ≥ 85: 51.5 p≥0.05
Associations of birth size, infancy, and childhood growth with intelligence quotient at 5 years of age: a Danish cohort study.<br>**Kirkegaard, 2020**<br>Denmark<br>Cohort	To examine how birth size and growth in infancy and childhood are associated with IQ at age 5 y in term-born children using path analysis.	This study was based on data from the Lifestyle During Pregnancy Study (LDPS) nested within the Danish National Birth Cohort (DNBC), which included women between 1996 and 2002 at their first antenatal visit to their general practitioner if they planned to carry their pregnancy to term and spoke Danish. The LDPS is a follow-up study of a subsample of the cohort, conducted between 2003 and 2006	Head circumference z-scores [at birth, 5 and 12 months, and 5 years].	Intelligence/Cognition: Wechsler Primary and Preschool Scales of Intelligence– Revised (WPPSI-R), including 3 verbal (arithmetic, information, and vocabulary) and 3 performance subtests (block design, geometric design, and object assembly). Swedish norms were used to derive scaled scores and IQs [5 years].	Sex, gestational age, socio-ocupational status, birth order, maternal age, maternal IQ, maternal BMI, paternal BMI, smoking, exercise and dietary intake during pregnancy, age when started in daycare, duration of any breastfeeding, time of introduction to solid food (Model A) + weight, height (Model B).	Trajectory analysis (betas):Model A: At birth: 0.52 (CI 95%:0.02;1.02); 5 months: 0.23 (CI 95%:−0.65;1.12); 12 months: 1.87(CI 95%:0.21;3.53); 5 years: 2.36 (CI 95%:1.02;3.71); Model B: At birth: 0.41 (CI 95%:−0.09;0.91); 5 months: 0.22 (CI 95%:−0.69;1.13) ; 12 months: 1.73 (CI 95%:−0.05;3.50); 5 years: 2.09(CI 95%:0.78;3.41)

**Title, author, year, country, study-design**	**Objective**	**Study sample [N]**	**Exposure**	**Outcomes**	**Confounders**	**Main Results**
		when the children turned 5 y of age, and the sampling was stratified on alcohol exposure. Twins, children who presented hearing or vision impairment, or congenital disease were excluded [N=1719].				
Very low birth weight and growth to age 8 years. II: Head dimensions and intelligence. **Kitchen, 1992** Australia Cross-sectional	(1) To investigate differences in OFC percentile distribution using the two commonly used reference standards; (2) to determine head growth achieved by age 8 years in cohorts of ELBW and VLBW children and a normal-birth-weight compari¬ songroup; (3) to investigate whether correction of OFC for the extremely dolichocephalic skull was of clinical importance at age 8 years; and (4) to determine if head measurements other than OFC were more strongly correlated with intelligence.	White children, who were born at the Royal Women's Hospital, Melbourne, Australia, with no signs of moderate or severe cerebral palsy, surviving to age 8 years. Group 1 included 79 children with birth weight between 500 and 999 g born between January 1,1977, and March 31,1982. Group 2 was composed of 111 children with birthweight between 1000 and 1499 g born between October 1, 1980, and March 31, 1982. Group 3 was composed of 56 randomly selected children with birthweight over 2500 g born during the same period as group 2 children [N= 151].	Head circumference z-scores according to age and sex [8 years].	Intelligence/Cognition: Wechsler Intelligence Scale for Children-Revised (WISC-R) [8 years].	Social class, maternal schooling, sex, birth weight, imigration status, fee-paying status.	Correllation: 0.24 p=0.003; Linear Regression (Beta): 64.5 (CI95%:5.3;123.7) p=0.034

Title, author, year, country, study-design	Objective	Study sample [N]	Exposure	Outcomes	Confounders	Main Results
Is big smart?: The relation of growth to cognition. **Klein, 1972** Guatemala Cross-sectional	To investigate whether physical growth, as an index of nutritional history, is related to cognitive functioning independent of social environmental factors.	Spanish-speaking children with 3 to 6 years of age, living in four isolated, rural villages in eastern Guatemala. They are part of a continuing longitudinal study of the relations among nutrition, physical growth, psychological performance and social experience [N=342]	Head circumference [3-6 years].	Intelligence/Cognition: Language facility: based on the child's ability to name and recognize pictures of common objects and to note and state the relations among orally presented verbal concepts; Short term memory for numbers: based on child's recall of increasingly long strings of numbers read to him at the rate of one per second; Perceptual analysis: based on child's ability to analyze complex visual arrays and to locate hidden figures embedded in a larger background or to detect which of several similar variations of an illustrated object was identical with a standard. Results were standardized to ager and sex [3-6 years].	Height; statified by sex.	Linear Regression (CI and p value were not presented): Pearson Correlation: Language facility: Boys: 0.23; Girls: 0.29; Short term memory: Boys: 0.18; Girls: 0.33; Perceptual analysis: Boys: 0.31; Girls: 0.32; Betas: Language facility: Boys: 0.20; Girls: 0.19; Short term memory: Boys: 0.18; Girls: 0.25; Perceptual analysis: Boys: 0.27; Girls: 0.28;
Patterns of cognitive development in very low birth weight children during the first six years of life. **Koller, 1997** United States Cohort	(1) To determine patterns of individual differences in cognitive development over the first 6 years of life in very low birth weight children, and (2) to examine the relative im- pact of selected biomedical and sociodemographic factors on these patterns.	Very low birth weight infants, born between 1975 and 1989 and recruited from the Neonatal Intensive Care Units of three hospitals in the Bronx that were part of the Department of Pediatrics of the Albert Einstein College of Medicine. Criteria for inclusion in the present study were a birth weight <1500 g and cognitive data at four points in time [N=203].	Head circumference percentile, dichotomized into ≤ 10th percentile and > 10th percentile (reference group) [1 year corrected age].	Intelligence/Cognition: Mental Development Index (MDI), of the Bayley Scales of Infant Development [12 and 24 months]; Stanford-Binet Intelligence Scale [4 years]; Wechsler Intelligence Scale for Children—Revised [6 years]. Using the cognitive test scores described above, a hierarchical agglomerative cluster analysis (Ward's method) was performed to identify	No adjustment was performed.	Chi-squared test: % Head circumference < 10th percentile for cognition cluster: Cluster A (average–stable): 18%; Cluster B (average–declined to low average): 21%; Cluster C (average–declined to below average): 32%; Cluster D (very low–increased to low average): 27%; Cluster E (very low–stable): 68% $p < 0.005$

Title, author, year, country, study-design	Objective	Study sample [N]	Exposure	Outcomes	Confounders	Main Results
				relatively homogeneous subsets of children with similar developmental patterns. Authors selected the cluster solution that best represented the data.		
Association between head circumference at two years and second and fifth year cognition. **Koshy, 2021** India Cohort	To assess the relationship between HC measured at two years and cognition at two and five years of age in a birth-cohort in south India.	Sub-analysis of a large multinational prospective, longitudinal birth-cohort study in eight different countries across the world -'The Etiology, Risk Factors and Interactions of Enteric Infections and Malnutrition and the Consequences for Child Health and Development (MAL-ED) Network' . The Indian study was conducted in an urban slum in Vellore, South India and recruited pregnant women identified by a door- to-door survey between March 2010 and February 2012. The exclusion criteria were family's existing plans to migrate out of the study site during the study period, multiple pregnancies, medical comorbidities in the index child, and another child from the same family already registered in the study [N=251].	Head circumference z-scores according to age, categorized into three groups: <−3 SD, −2 SD to -3 SD and ≥− 2SD (reference group) [2 years]	Intelligence/Cognition: The Bayley Scales of Infant and Toddler Development- III (BSID-III) [2 years]; The Wechsler Preschool Primary Scales of Intelligence – third edition (WPPSI-III) [5 years].	Socio-economic status (WAMI scores), Maternal IQ, height, sex, mean body iron and mean body lead.	Linear Regression (beta): BSID-III: Head circumference from − 2 to −3 SD: − 0.16 (CI 95%:− 1.07;0.75) p= 0.730; < − 3 SD: − 2.21 (CI 95%:− 3.87;-0.56) p=0.009; WPPSI-III: Verbal IQ: − 2 to −3 SD: −1.58 (CI 95%:− 4.06;0.90) p= 0.211; < − 3 SD: − 7.35 (CI 95%: −11.78; -2.92) p= 0.001; Performance IQ: − 2 to −3 SD: −1.36 (CI 95%: − 3.99;1.28) p= 0.311; < − 3 SD: −7.07 (CI 95%: −11.77;-2.36) p= 0.003; Processing speed: − 2 to −3 SD: 1.00 (CI 95%: −3.90;5.89) p= 0.688; < − 3 SD: −7.47 (− 16.39;-1.46) p= 0.101

Title, author, year, country, study-design	Objective	Study sample [N]	Exposure	Outcomes	Confounders	Main Results
Associations between physical growth and general cognitive functioning in international adoptees from Eastern Europe at 30 months post-arrival **Kroupina, 2015** United States Cohort	To examine the association between physical growth, the growth hormone (GH) system, and general cognitive functioning post-adoption.	Children who were adopted in EUA from Eastern Europe, between 8.8 and 45.6 months of age, recruited when their initial medical evaluation was scheduled with their parents within 1 month of their arrival in the USA. Children with a high risk for fetal alcohol spectrum disorder (FASD) were excluded [N=46].	Head circumference z-scores according to age and sex [within 1 month of adoption, 6 and 30 months after adoption]	Intelligence/Cognition: Stanford-Binet Intelligence Scales (fifth edition), converted to normalized, standard scores with means of 100 and standard deviations of 15 [30 months after adoption].	Age at arrival, IGFBP-3 (IGF binding protein-3) and Mullen Scales of Early Learning at 6 months	Spearman Correlation: Head circumference at arrival and verbal IQ: 0.34 p=0.010; Head circumference at arrival and non-verbal IQ: 0.25 p=0.050; at 6 months and verbal IQ: 0.33 p=0.014; At 6 months and non-verbal IQ: 0.19 p=0.099; at 30 months and verbal IQ: 0.21 p=0.172 at 30 months and non-verbal IQ: 0.11 p=0.454 Linear Regression (Beta): Change in head circumference 6 months after arrival and verbal IQ: 0.280 p=0.031
Developmental correlates of head circumference at birth and two years in a cohort of extremely low gestational age newborns. **Kuban, 2009** United States Cross-sectional	To evaluate the developmental correlates of microcephaly evident at birth and at 2 years in a cohort born at extremely low gestational age.	During the years 2002–2004, women delivering before 28 weeks gestation at 14 participating institutions in 11 cities in 5 states in United States were asked to enroll in the study (ELGAN Study). This analysis included infants who survived to 24 months corrected age [N=958].	Head circumference z-scores dichotomized into < - 2 SD ≥ -2 SD (reference group) [24 months corrected age]	Intelligence/Cognition: Mental Developmental Index (MDI) - Bayley Scales of Infant Development, Second edition. Children who could not complete Bayey Scales were evaluated using the Vineland Adaptive Behavior Scales (MDI equivalent). Results were dichotomized into MDI < 70 and ≥ 70 [24 months corrected age].	Ventriculomegaly on late (third) ultrasound scan; Hypoechoic lesions	Logistic Regression (Odds Ratio): Adjusted for ventriculomegaly: 2.8 (CI 95%:2.0;3.8); Adjusted for hypoechoic lesions: 2.7 (IC 95%:2.0;3.8); Adjusted for both: 2.7 (IC 95%:2.0;3.7)
School difficulties in 20-year-olds who were born small for gestational age at term in a regional cohort study. **Larroque, 2001** France Cohort	To investigate the relation between school difficulties and being born small for gestational age (SGA) at full term in adolescents and young adults.	Full-term (>37 weeks' gestation) singleton patients who were born small for gestational age (birth weight or length below the third percentile), identified from a population-based birth registry in and around the city of	Head circumference z-scores according to age and sex, dichotomized into < - 2 SD ≥ -2 SD (reference group) [at birth]	Academic Performance: Age at starting secondary school (normally 11) - Participants who were older (≥12 years) had repeated at least 1 grade in primary school; and the final examination of secondary school, the baccalaureate. This	Socioeconomic level, maternal age, sex, family size.	Logistic Regresion (Odds Ratio):Late entry in secondary school - crude: 2.2 (CI95% was not presented) p=0.04; adjusted: 1.8 (CI95%:0.7;4.4) p= 0.20; No baccalaureate: crude: 2.3 (CI95% was not presented) p=0.05;

Title, author, year, country, study-design	Objective	Study sample [N]	Exposure	Outcomes	Confounders	Main Results
		Haguenau, France, from 1971 to 1978. For this analysis, adolescents who had psychomotor handicaps, severe chronic illnesses or malformations, or institutionalised were excluded [N=236].		diploma is not obtained by adolescents who failed it and those who took a short vocational program. The normal age for the baccalaureate is approximately 18 years. We analyzed baccalaureate success only among participants who were at least 19 years old [16-24 years].		adjusted: 1.9 (CI95%:0.7;5.1) p= 0.19;
The causal influence of brain size on human intelligence: Evidence from within-family phenotypic associations and GWAS modeling. **Lee, 2019** United States Unsure - lack of information	To investigate whether the association between brain size and the IQ reflects a theoretically important causal relationship or spurious confounding.	The data are derived from the Minnesota Center for Twin and Family Research, including monozygotic (MZ) and same-sex dizygotic (DZ) twin pairs born in Minnesota from 1972 to 1984, identified from Minnesota state birth records. Participants were recruited when they were approximately 11 or 17 years of age [N=2698].	Head circumference z-scores according to age and sex [11 or 17 years]	Intelligence/Cognition: Wechsler Intelligence Scale for Children-Revised (WISC- R) [11 years-old twins] and Wechsler Adult Intelligence Scale-Revised (WAIS-R) [17 years-old twins] - short forms, including two Verbal subtests (Information, Vocabulary) and two Performance subtests (Block Design, Picture Arrangement) and standardized with mean zero and unit variance.	Analysis with family fixed effects	Linear Regression (Betas): All pairs (N=1349): 0.172 p=0.00000003; MZ 11 years (N=642): 0.150 p=0.002; DZ 11 years (N= 363): 0.219 p=0.00005; MZ 17 years (N= 223): −0.050 p= 0.56; DZ 17 years (N=121): 0.209 p=0.05;<br><br>Upon inclusion of the first three powers of standardized height and weight as covariates, the coefficient of HC becomes slightly smaller but remains highly significant (Beta: 0.148, p < 0.0001)
Choosing the Best Newborn Anthropometric Measure Associated With the Risks and Outcomes of Intrauterine Growth Restriction **Lei, 2015** United States Cohort	To test 8 anthropometric measures at birth and selected the most relative anthropometric measure at birth with intra uterine growth restriction-related risks and outcomes in term newborns.	The Collaborative Perinatal Project was a prospective study that recruited pregnant women at 12 US academic medical centers between 1959 and 1976. Stillbirths or terminations, multiple births, preterm (gestational age <37 weeks), postterm	Head circumference percentile according to gestational age, sex and ethnicity, dichotomized into head circumference < 10th and ≥ 10th percentile	Intelligence/Cognition: Wechsler Intelligence Scales for School Children and Preschool Children (WISC-I), based on 7 components (information, comprehension, vocabulary, digit span, picture arrangement, block design, and coding). IQ <70 was used as the long-	Socioeconomic level, maternal age, magternal schooling, parity, smoking during pregnancy, gestational age, sex, breastfeeding, race, marital status, hypertensive disorders during pregnancy, delivery	Logistic Regression (Odds Ratio): 1.9(IC 95%:1.6;2.4)

Title, author, year, country, study-design	Objective	Study sample [N]	Exposure	Outcomes	Confounders	Main Results
		(gestational age ≥42 weeks), or unknown gestational age were excluded from this analysis. The sample was restricted to non-Hispanic white and black [N=30327].	(reference group) [at birth].	term adverse outcome in this analysis [7 years].	hospital, Apgar score at 5 minutes.	
Antenatal and postnatal growth and 5-year cognitive outcome in very preterm infants. **Leppanen, 2014** Finland Cohort	To study how antenatal growth affects cognitive outcome in very preterm infants and to determine whether there is an association between growth in any particular time period between birth and 5 years of age and cognitive outcome.	Infants who were born between 2001 and 2006 with a birth weight <1501 g or between 2004 and 2006 with a gestational age <32 weeks, treated at Turku University Hospital, and from Finnish- or Swedish-speaking families living in the catchment area to enable psychological assessment. Infants with severe congenital anomalies or a diagnosed syndrome affecting their development were excluded [N=181].	Head circumference z-scores according to age [at birth, 36 and 40 weeks, 1, 2 and 4 months, 1, 2 and 5 years] .	Intelligence/Cognition: Short version of the Wechsler Preschool and Primary Scales of Intelligence–Revised, including subtests for information, sentences, arithmetic, block design, geometric design, and picture completion, and standardized to mean 100 and SD 15 [5 years].	Maternal schooling, gestational age, sex.	Spearman Correlation: varied between 0.11 and 0.18 for all children, with p< 0.05 in 4 of 7 measures Linear Regression (Beta): For incresing in 1 z-score of head circumference from birth to 1 year: 2.5 (CI 95%: 0.67;4.33) p=0.008; 40 weeks to 1 year: 3.6 (CI 95%: 0.95;6.15) p=0.007; birth to 2 years: 2.5 (IC 95%: 0.65;4.31) p=0.008; 40 weeks to 2 years: 3.1 (CI 95%:0.45;5.70) p=0.02
Language and motor findings in benign megalencephaly. **Lewis, 1989** United States Cross-sectional	To document the language, motor and intelli- gence functions in children presenting with head circumferences greater than two standard deviations above normal but for whom no known underlying cause exists beyond genetic factors. Findings are	Children between the ages of 5 and 12 years, followed in the clinical neurological practice of the third author and met the following criteria: head circumference was greater than the 98th percentile for age and sex; no central nervous system pathology or metabolic disease was known to be associated with increased brain size, tomography scan	Head circumference percentile according to age and sex, dichotomized into > 98th and ≤ 98th percentile (reference group) [3-12 years]	Intelligence/Cognition: Slosson Intelligence Test; Two measures of language comprehension were included: the Peabody Picture Vocabulary Test assessed vocabulary comprehension; and the Token Test for Children measured syntactic comprehension [3-12 years].	No adjustment was performed, but bivariate analysis comparing the groups of siblings.	Mann-Whitney U test - Peabody Picture Vocabulary Test (U = 33.0, p = 0.51); Token Test (U = 40.0, p = 0.97); Similarly, no difference in IQ was noted (data not presented).

Title, author, year, country, study-design	Objective	Study sample [N]	Exposure	Outcomes	Confounders	Main Results
	compared to siblings' per-formance who have normal head circumferences.	showed a normal ventricular system and the absence of other congenital or acquired disease processes, lived with both parents and at least one sibling above 3 year of age in the home. The sibling group consisted of siblings with normal head circumferences (i.e., within two standard deviations from the mean for age and sex) [N=18].				
Relative importance of birth size and postnatal growth for women's educational achievement.<br>**Li, 2004**<br>Guatemala<br>Cohort	To examine prenatal growth (proxied by size at birth), early childhood growth (by size at age 2 years, which represents the prenatal and the early postnatal periods), and total growth (by adult height) in relation to women's educational achievement (EA); and to assess the relative importance of birth size, early postnatal growth (birth to age 2 years), and late postnatal growth (2 years to adulthood) for women's educational achievement.	Female singletons, who were born between August 1969 and December 1975, participated in the 1969–1977 INCAP study, were residents of the villages between 1996 and 1999, and had at least one child less than 3 years of age during this time (who took part in a second longitudinal study carried out by Emory and INCAP) [N=133].	Head circumference z-scores calculated according to the revised 2000 NCHS/CDC Growth Charts. The authors estimated the influence of birth size on size at 2 years by modeling head cicrumference at 2 years on birth size to estimate predicted size at 2 years. Then, the residual (observed size at 2 years – predicted size at 2 years, R1) was calculated as a measure of the early postnatal effect [at birth, 2	Academic Performance: Summary measure of "Educational achievement", based on the tests of general knowledge, numeracy, reading, and two educational achievement tests. Results were categorized into quintiles, based on cohort-specific threshold values [20-29 years].	Socioeconomic level (based on characteristics of home and possessions, mother's education, and father's occupational status), maternal age, gestational age, age at follow-up, maximum grade achieved	Logistic Regression (Odds Ratio): Head circumference at birth: 0.9 (CI95%:0.7; 1.3); at 2 years: 1.4 (CI95%:1.02; 2.0); From birth to 2 years: 1.5 (CI95%:1.05; 2.2)

Title, author, year, country, study-design	Objective	Study sample [N]	Exposure	Outcomes	Confounders	Main Results
			years, from birth to 2 years].			
Growth in very preterm children: Head growth after discharge is the best independent predictor for cognitive outcome. **Lidzba, 2016** Germany Cohort	To investigate the relationship between growth and cognitive/motor development at early school-age.	Very preterm /very low birth weight infants (<32 weeks gestation or <1500 g) born between 1995 and 1997, treated in one neonatal intensive care unit of a tertiary centre (University Children's Hospital, Tübingen, Germany. Children with cerebral palsy were excluded [N=83].	Head circumference z-scores; Catch-up growth, defined as difference between age-standardized head circumference at the second measure and age-standardized head circumference at first measure [at birth, at discharge, 6.7-10 years].	Intelligence/Cognition: Kaufmann Assessment Battery for Children (K-ABC) - The Mental Processing Composite Score (MPC), which is calculated from the simultaneous processing scale (visuo-spatial and logical abilities) and the sequential processing scale (measuring auditory and visual short term memory and attention) [6.7 - 10 years].	Maternal schooling, "immaturity" (gestational age, days of ventilation, corticosteroids, BPD), intracranial haemorrhage, sepsis.	Linear Regression (change in the coefficient of determination - R2 - %): Head circumference at birth: 0 %, p=0.982; Catch-up from birth to discharge 5%, p= 0.030; Catch-up from discharge to school age: 13.4%, p < 0.001
Early head growth: relation with IQ at 8 years and determinants in term infants of low and appropriate birthweight. **Lira, 2010** Brazil Cohort	To investigate the relation between head growth at different periods and IQ at 8 years, and to identify factors associated with more rapid head growth.	Term infants born at any of the six maternity centres in the state of Pernambuco, northeast Brazil, between January 1993 and August 1994 who lived in the study area and came from families earning less than four times the minimum wage (minimum wage approximately US$70 per month). Each time a low birth wight (from 1500 to 2499g) baby was recruited then the next appropriate birth weight (from 3000 to 3499g) infant of the same sex was recruited.	Head circumference z-scores using the updated British 1990 Growth Reference. Gain in head circumference between successive time points, conditional on size at all earlier time points, was calculated [at birth, 2, 4, 6, 12 and 24 months, and 8 years].	Intelligence/Cognition: Wechsler Intelligence Scale for Children (WISC III) validated for the Brazilian population to measure IQ [8 years].	Maternal age, maternal schooling, family income per head, environmental index, home stimulation index, crowding, cohabitation, child's type of school and years of schooling, measured at 8 years	Linear Regression (betas): Low birthweight group: Head circumference at birth - crude: 1.36(CI95%:-3.16;5.87); adjusted: 0.70 (CI95%:-3.12;4.53); Conditional head gain: Between birth and 2 months - crude: 3.59 (CI95%:0.72; 6.46); adjusted: 3.31 (CI95%:0.82;5.80); between 2 and 6 months - crude: 5.02 (CI95%:2.24;7.81); adjusted: 4.82 (CI95%:2.37; 7.26); between 6 months and 8 years- crude: 1.19 (CI95%:-1.81;4.20); adjusted: 1.17 (CI95%:-

Title, author, year, country, study-design	Objective	Study sample [N]	Exposure	Outcomes	Confounders	Main Results
		Infants with congenital anomalies, twins, and those with signs of neurological abnormalities in the first 24 hours of life were excluded [N=164].				1.35;3.70); Appropriate birthweight group: Head circumference at birth: - crude: 1.32 (CI95%:-2.59;5.22); adjusted: 2.40 (CI95%:-0.89;5.68); Conditional head gain: Between birth and 2 months - crude: 3.75 (CI95%:0.66;6.85); adjusted: 1.82 (CI95%:-0.98; 4.62); between 2 and 6 months - crude: 3.22(CI95%:0.07;6.37) adjusted: 1.76 (CI95%:-1.00;4.53); between 6 months and 8 years - crude: 1.01 (CI95%:-2.23;4.25); adjusted: 1.32 (CI95%:-1.40;5.68).
Indices of body and brain size at birth and at the age of 2 years: relations to cognitive outcome at the age of 16 years in low birth weight infants.<br>**Lorenz, 2009**<br>United States<br>Cohort	To determine in low birth weight infants the relations of being small for gestational age at birth, microcephalic at birth, low weight for age at 2 years, and microcephalic at 2 years to full scale intelligence quotient (FSIQ) at the age of 16 years.	Participants belong to the age of 16 years follow-up of the Neonatal Brain Hemorrhage Study birth cohort, which enrolled infants with birth weights <2000 g born within or admitted to newborn intensive care units in 3 New Jersey hospitals between September 1, 1984, and June 30, 1987 [N=422].	Head circumference percentile dichotomized into head circumference < 10th and ≥ 10th percentile (reference group) [at birth, 2 years].	Intelligence/Cognition: Wechsler Abbreviated Scales of Intelligence (WASI) - Full scale intelligence quotient (FSIQ), including 4 subtests (2 verbal and 2 nonverbal) and standardized to mean of 100 and SD of 15) [16 years].	Weight, age, at follow-up, small for gestational age, any birth social risk (maternal age <19 years, maternal schooling < high school education, unmarried status, minority status, and receipt of public assistance) , germinal matrix and/or intraventricular hemorrhage on head ultrasound, parenchymal lesion and/or ventricular enlargement on head ultrasound, placental abruption, Apgar, thyroid status, systolic blood	Bivariate analysis (Mean difference): Head circumference at birth: 4.2 p=0.001; at 2 years: 7.9 p< 0.001<br>Linear regression (betas): Head circumference < 10th percentile at birrth: -2.12 (CI95%:-6.01;1.77); at 2 years: -4.11 (CI95%:-7.35; -0.87)

Title, author, year, country, study-design	Objective	Study sample [N]	Exposure	Outcomes	Confounders	Main Results
					pressure, peak bilirrubin, prolonged ventilation for gestacional age, any nonelective post-discharge hospitalization. The two head circumference measures were included in the model simultaneously.	
Intellectual and psychological performance in males born small for gestational age with and without catch-up growth. **Lundgren, 2001** Sweden Cohort	To analyze whether body size at birth among males is associated with subsequent intellectual performance and psychological performance in early adulthood.	Men who were born in Sweden from 1973 to 1978 (The Swedish Birth Register), who survived up to 18 years of age and conscripted between January 1991 and January 1997. Multiple births, congenital malformations, and infants born to non-Nordic mothers were excluded [N= 248051].	Head circumference z scores according to gestational age, categorized into three groups: <−2 SD, −2 SD to 2 SD (reference group), and >2 SD [at birth]	Intelligence/Cognition: General intellectual performance, measured by a time-limited test package including four dimensions: logical/inductive, verbal, spatial, and theoretical/technical. The test questionnaire contains 160 items, 40 from each dimension; The results are presented as standard nine (stanine) scores (mean = 5, SD = 2). Subnormal performance was defined as a score of ≤ 3 (i.e. less than -1 SD) [at conscription for military service, 18–25 years]	Idade gestacional, Comprimento ao nascer para idade gestacional; Peso ao nascer para a idade gestacional; altura no alistamento militar	Logistic Regression (Odds Ratio): Head cicrcumference < -2 SD: Crude: 1.45 (CI95%:1.40;1.50); Adjusted: 1.28 (CI95%:1.21;1.36); Head cicrcumference > 2 SD: Crude: 0.85 (CI95%:0.78;0.91); Adjusted: 0.83(CI95%:0.77;0.89);
Birth characteristics and different dimensions of intellectual performance in young males: a nationwide population-based study. **Lundgren, 2003** Sweden	To study the different dimensions of intellectual performance related to birth characteristics and final height.	Men who were born in Sweden from 1973 to 1976 (The Swedish Birth Register), who survived up to 18 years of age and conscripted up to 1994. Multiple births, congenital malformations, and infants born to non-	Head circumference z scores according to gestational age, categorized into three groups: <−2 SD, −2 SD to 2 SD (reference group), and >2 SD [at birth]	Intelligence/Cognition: General intellectual performance, measured by a time-limited test package including four dimensions: logical/inductive, verbal, spatial, and theoretical/technical. The test questionnaire contains 160 items, 40 from each dimension; The results are	Peso ao nascer, comprimento ao nascer, Idade gestacional, IMC no alistamento militar	Logistic Regression (Odds Ratio): All sample: Head circumference < -2 SD: logical: 1.28 (CI95%: 1.20;1.35); spatial: 1.12 (CI95%: 1.06;1.19) theoretical: 1.14 (CI95%:1.08;1.20) verbal: 1.14 (CI95%1.07;1.20); Head circumfence > 2 SD: logical: 0.78 (CI95%:

Title, author, year, country, study-design	Objective	Study sample [N]	Exposure	Outcomes	Confounders	Main Results
Cohort		Nordic mothers were excluded [N= 193723].		presented as standard nine (stanine) scores (mean = 5, SD = 2). Subnormal performance was defined as a score of ≤ 3 (i.e. less than -1 SD) [at conscription for military service, 18 years]		0.74;0.83) spatial: 0.89 (CI95%:0.84;0.94) theoretical: 0.88 (CI95%: 0.84;0.93) verbal: 0.88 (CI95%: 0.83;0.93) Small for gestational age:Head circumference < -2 SD: logical: 1.33(CI95%: 1.15;1.55); spatial: 1.08 (CI95%:0.94;1.25); theoretical: 1.20 (CI95%:1.04;1.38); verbal: 1.19 (CI95%:1.03;1.38)
Short Adult Stature and Overweight Are Associated with Poor Intellectual Performance in Subjects Born Preterm **Lundgren, 2011** Sweden Cohort	To study the association between pre-term birth and adult intellectual performance, with special emphasis on the influence of postnatal growth.	The study is part of a large population-based cohort study on infants born with a low birth weight and low birth length, using the Swedish Medical Birth Register linked to the Swedish Conscript Register, which included liveborn male infants born between 1973 and 1978 who conscripted between January 1991 and January 1997. Infants born of non-Nordic mothers and multiple births were excluded (N = 242244)	Head circumference z scores according to gestational age, categorized into three groups: <−2 SD, −2 SD to 2 SD (reference group), and >2 SD [at birth]	Intelligence/Cognition: General intellectual performance, measured by a time-limited test package including four dimensions: logical/inductive, verbal, spatial, and theoretical/technical. The test questionnaire contains 160 items, 40 from each dimension; The results are presented as standard nine (stanine) scores (mean = 5, SD = 2). Subnormal performance was defined as a score of ≤ 3 (i.e. less than -1 SD) [at conscription for military service, 18 years]	Idade gestacional, peso ao nascer, comprimento ao nascer, índice de Apgar, altura e IMC no alistamento	Logistic Regression (Odds Ratio): : Head cicrcumference < -2 SD - Crude: 1.45 (CI95%:1.37;1.52); Adjusted: 1.27 (CI95%:1.19;1.35); Head cicrcumference >2 SD - Crude 0.81 (CI95%:0.75;0.87); Adjusted: 0.82 (CI95%:0.75;0.89)
Neighbourhood socioeconomic status and maternal factors at birth as moderators of the association between birth characteristics and school attainment: a	To investigate whether reading and writing skills among children of equivalent perinatal characteristics differ by neighbourhood socioeconomic	Non-Aboriginal children in Western Australia who had been born between 1990 and 1997 and subsequently attended Western Australian government schools during 2000– 5, who had a school record	Percentage of optimal head circumference - The optimum value for each measure for a given gestational duration, gender, maternal height,	Academic performance: The reading and writing tasks assessed children's ability to effectively read and write in everyday life and were measured in two literacy test scores. The reading test consisted of multiple-choice, short- and	Maternal age, gestational age, sex, percentage of optimal birth weight at birth, percentage of optimal height at birth, Apgar score, birth order, mother`s marital status, Index	Linear Regression - Multilevel model, participant and school (betas): Reading: 0.46 (CI95%:0.27;0.64); Writing: 0.37(CI95%:0.14;0.60)

Title, author, year, country, study-design	Objective	Study sample [N]	Exposure	Outcomes	Confounders	Main Results
population study of children attending government schools in Western Australia. **Malacova, 2009** Australia Cohort	status and maternal factors.	for grade three. Multiples and children with missing predictor variables were excluded [N=55533].	parity and age is estimated from models derived from Western Australian neonatal survivors unaffected by the most frequently occurring pathological determinants of intrauterine growth in the Western Australian population [at birth].	open-response questions, whereas the writing test required writing a short story, fable, or an anecdote [third grade, mean 8.2 years].	of Relative Socioeconomic Disadvantage (residencial area level), Index of Education and Occupation (district level), school socioeconomic status, academic year, language background, mothers ethnicity, school.	
Developmental changes in head-circumference and mental-performance growth rates: a test of Epstein's phrenoblysis hypothesis. **McCall, 1983** United States Cohort	To test Epstein's phrenoblysis hypothesis, which states that brain growth and mental growth occur in correlated spurts at 3-10 months and 2-4, 6-8, 10-12 or 13, and 14-16 or 17 years of age.	Children who were born between 1930 and 1938, reared in small-to-medium-sized south-western Ohio towns, selected from the Fels Longitudinal Study because they had relatively complete sets of longitudinal I.Q. assessments between 2.5 and 17 years of age [N=80].	Head circumfernce (2.5, 3, 3.5, 4, 4.5, 5, 5.5, 6, 7, 8, 9, 10, 11, 12, 14, 15, e 17 years)	Intelligence/Cognition: Mental age – IQ calculated using Stanford-Binet, transformed into mental ages and then into annualized mental-age growth rates for each assessment age from 3 to 17 years [2.5,3,3.5,4,4.5,5,5.5,6,7,8, 9, 10, 11, 12, 14, 15, and 17 years].	No adjustment was performed.	Multiple discriminant analysis:A significant relationship was not found between patterns of growth rate in head circumference and patterns of growth rate in mental age between 2.5 and 17 years of age (p=0.14)
Outcomes of children adopted from Eastern Europe. **Miller, 2009** United States Cohort	To evaluate cognitive, behavior, and family stress results in a group of 8–11-year-old international adoptees from Eastern Europe compared to child characteristics at arrival.	Children eligible to participate were: (1) adopted from Eastern Europe/former Soviet Union, (2) currently age 8 years 0 months to 10 years 11 months, and (3) with their adoptive families for more than five years. Children were recruited through an International Adoption Clinic	Head circumference z-scores according to age [at arrival - meang age 21 months, at the current evaluation - mean age 9.2 years]	Intelligence/Cognition: Wechsler Abbreviated Scale of Intelligence (WASI) - verbal, performance, and full scale IQ [mean 9.2 years].	No adjustment was performed.	Correlation (without specification): Current head circumferencel - verbal IQ: 0.41, p=0.002; performance IQ: 0.29, p=0.03, total IQ: 0.40, p=0.003); Head circumference at arrival - total IQ: 0.071 p>0.05

Title, author, year, country, study-design	Objective	Study sample [N]	Exposure	Outcomes	Confounders	Main Results
		database and by mailings, announcements, and advertisements in local adoption newsletters [N=50].				
Nutritional and developmental status among 6- to 8-month-old children in southwestern Uganda: a cross-sectional study. **Muhoozi, 2016** Uganda Cross-sectional	To assess the nutritional status and milestone development of 6- to 8-month-old children and associated factors in two districts of southwestern Uganda.	The sampling unit was a household with a lead caregiver as the respondent and an infant aged 6-8 months in Kabale and Kisoro, between October 2013 and February 2014. Households were excluded if the child had 1) congenital malformation(s), 2) a physical handicap that would influence growth or preclude anthropometric measurements, or influence nutrient intake, or 3) been diagnosed with mental or brain illness as evidenced by the child's mother or a health worker [N=512].	Head circumference z-scores (6-8 months)	Intelligence /Cognition: Bayley Scales of Infant and Toddler Development (BSID III) , sandardized to mean 100 e SD 15 [6-8 motnhs].	No adjustment was performed.	Pearson Correlation: 0.08 p>0.05 Linear regression was performed, howerver head circumfernce was not included in the final model.
Pattern of growth of very low birth weight preterm infants, assessed using the WHO Growth Standards, is associated with neurodevelopment. **Nash, 2011** Canada Cohort	To determine whether the pattern of growth of very low birth weight infants during the first 2 years, assessed using the WHO-GS or the traditional Centers for Disease Control and Prevention reference growth	In this retrospective chart review, all preterm infants with birth weights of ≤1500 g, who were born between August 2004 and October 2006, and who were cared for at Sunny- brook Health Sciences Centre in Toronto were eligible for inclusion. Infants	Head circumference Z-scores, using both WHO-GS and CDC graphics to comparision. Sustained growth was defined for each parameter as a change in Z-score of ≤1SD, decelerated	Intelligence/cognition: Bayley Scales of Infant and Toddler Development (BSID III), sandardized to mean 100 e SD 15 [18-24 months of corrected age].	Gestational age, sex, inborn/outborn status, multiple birth, necrotizing enterocolitis (Bell stage ≥II), confirmed nosocomial infection, peri- and (or) intraventricular hemorrhage (grade ≥3), chronic lung	Mean (ANCOVA): Decelerated growth - WHO: 101.9, CDC: 103.0; Sustained growth - WHO: 104.4, CDC: 104.2; Accelerated growth - WHO: 104.8, CDC: 105.4. The differences were not statistically significant.

Title, author, year, country, study-design	Objective	Study sample [N]	Exposure	Outcomes	Confounders	Main Results
	charts (CDC-RGC), is associated with neurodevelopment.	born with a serious congenital anomaly or depression at birth (Apgar score <5 at 5 min), or who were small for gestational age (below the tenth weight-for-age percentile at birth) or died during initial hospitalization were excluded [N=289].	growth was defined as a decline in Z-score of >1 SD, and accelerated growth was defined as an increase in Z-score of >1 SD [at birth, 6 weeks, 4, 8, 12 and 18-24 months of corrected age].		disease, and patent ductus arteriosus.	
Head size at one year as a predictor of four-year IQ **Nelson, 1970** United States Cohort	To determine whether there is an association between head circumference at one year of age and IQ at four years.	Data from the Collaborative Study on Cerebral Palsy, Mental Retardation, and Other Neurological and Sensory Disorders of Infancy and Childhood, which included single-born children, who were born between 1959 and 1964, products of the first Collaborative Project pregnancy of their mothers, who had one-year neurological examinations between the ages of 50 and 54 weeks and who had subsequent psychological examinations at four years. Children with definite organic heart disease at one year were excluded [N=9379].	Head circumference (centimeters) [1 year]	Intelligence/cognition: IQ, using Stanford-Binet Intelligence Test [at 4 years]	Maternal schooling, sex, ethnicity.	The authors did not conduct any statistical test, but presented a table with means/frequencies of IQ and head circumference, stratified by confounders. In the text: "The bottom line indicates the substantial association between IQ and the variable 'years of education of mother' while internal comparison provides a measure of the differences between races and between sexes. For whites of the Collaborative Project, the median four-year IQ is 104, while for Negroes it is 91; in almost every comparison median scores for females exceed those for males by about 3 points. The vertical trends within each of the cells of the table indicate a direct relation- ship between one-year head circumference and four-year IQ, with some slight falling- off in

Title, author, year, country, study-design	Objective	Study sample [N]	Exposure	Outcomes	Confounders	Main Results
						the average scores of those who have excessively large heads".
Poor postdischarge head growth is related to a 10% lower intelligence quotient in very preterm infants at the chronological age of five years. **Neubauer, 2016** Austria Cohort	To determine whether early head growth in very preterm infants was associated with cognitive outcome at the chronological age of five years.	Infants born alive at less than 32 completed weeks of gestation at the Medical University of Innsbruck, the only neonatal intensive care unit in the geographical region (Tyrol, a state in western Austria), from January 2003 to April 2009. Children with congenital anomalies, and nonresidents or moved out of the region prior to follow-up at the chronological age of five years were excluded [N=273].	Head circumference z-scores; suboptimal head size was defined as a Z score of >1 standard deviation (SD) below the mean [at birth, at discharge, at 3, 12 and 24 months, and at 5 years].	Intelligence/Cognition: Wechsler Preschool and Primary Scales of Intelligence, third edition (WPPSI-III). In 13 children, the Snijders-Oomen Non-Verbal Intelligence Test (was used because of language difficulties and, or, cognitive delay. Cognitive delay was defined as a full-scale IQ score of <85 (>1SD below the mean) in either the WPPSI-III or the SON-R test and significant delay as a score of less than 70 (>2SD below the mean) [at 5 years].	No adjustment was performed.	Head circumference Z-score - Mean (Mann–Whitney U-test): At birth: Normal IQ: 0.05; Abnormal IQ: 0.00 p=0.348; at discharge: Normal IQ: -0.54; Abnormal IQ: - 0.57 p=0.796; 3 months: Normal IQ: -0.21; Abnormal IQ: -1.14 p=0.001; 12 months: Normal IQ: -0.33; Abnormal IQ: -1.14 p<0.0001; 24 months: Normal IQ: -0.60; Abnormal IQ: -1.69 p<0.0001; 5 years: Normal IQ: -0.65; Abnormal IQ: -2.06 p=0.002.
Factors associated with head circumference and indices of cognitive development in early childhood **Nicolaou, 2020** Bangladesh, India, Nepal, Peru and South Africa Cohort	While head circumference has been related to intracranial volume and brain size, its association with cognitive function remains unclear. We sought to understand the relationship among various biological and socioeconomic risk factors, head circumference and cognitive development.	Data from study "The Etiology, Risk Factors, and Interactions of Enteric Infections and Malnutrition and the Consequences for Child Health", which included children recruited and followed between 2009 and 2014 in 8 low-and-middle-income-countries: Bangladesh (Dhaka, urbano), Brazil (Fortaleza, urbano), India (Vellore, urbano), Nepal (Bhaktapur, urbano), Peru (Loreto, rural), Pakistan	Head circumference z-scores - individual child slopes using a linear model of head circumference as a function of age in the intervals between test administrations. Measures were taken monthly from the first 2 weeks of life to 24 months [0–6 months, 6–15	Intelligence/cognition: Bayley Scales of Infant and Toddler Development - III (BSID III) [at 6, 15 and 24 months].	Socioeconomic level, maternal height, participant weight, breastfeeding, total energy intake in kilocalories, micronutrient levels (inflammation-adjusted ferritin and retinol), altitude-adjusted haemoglobin concentration, food insecurity, diarrheal episodes, longitudinal prevalence of fever, acute lung	Linear regression (beta): Graphics presented in the supplementary materials. In the text: We found no associations between cognitive, gross motor or language scores and any of the three HC measures as assessed at 6, 15 and 24 months of age.

Title, author, year, country, study-design	Objective	Study sample [N]	Exposure	Outcomes	Confounders	Main Results
		(Naushahro Feroze, rural), South Africa (Venda, rural), and Tanzania (Haydom, rural). Children with less than 1500 g at birth were excluded. Head circumference data collected from Pakistan and Brazil, and Bayley scale data collected from Tanzania were considered as low quality and were also excluded [N=1210].	months and 15–24 months].		respiratory infection (ALRI), antibiotic use, faecal path-ogen burden, alpha-1-acidglycoprotein (AGP) plasma concentration, myeloperoxidase (MPO), neopterin (NEO) and alpha-1 antitrypsin (AAT) concentrations, and lactulose:mannitol Z-score.	
Head circumference and long-term outcome in small-for-gestational age infants.<br>**Ochiai, 2008**<br>Japan<br>Cohort	To assess risk factors for the growth and development of small-for-gestational age (SGA) infants whose birth weight was less than the 10th percentile.	Low birth weight infants (birth weight less than the 10th percentile by Japanese growth norms) who were born from April 1995 to March 1998, and were admitted to the neonatal intensive care unit of three affiliated hospitals of Kyushu University Hospital, Fukuoka Municipal Children's Hospital and National Kyushu Medical Center. Patients who suffered from chromosomal abnormalities, inherited diseases, TORCH infections, and/or major constitutional anomalies, or infants of multiple births were excluded [N=56].	Head circumfernce percentile, dichotomised into head circumference ≥ 10th percentile (reference group) and < 10th percentile [at birth and at 1 year].	Intelligence/Cognition: Wechsler Intelligence Scale for Children (WISC-R) [6 years].	No adjustment was performed.	Mean (T test): Head circumference at birth: no difference (results presented in a Figure).Head circumference at 1 year < 10th percentile vs ≥ 10th percentile: Performance IQ: 82.4 (11.6) vs. 99.4 (16.8) p<0.01; Verbal IQ: 84.9 (15.8) vs. 104.3 (10.9) p< 0.005; Total IQ: 80.8(13.3) vs. 102.7 (15.0) p<0.005

Title, author, year, country, study-design	Objective	Study sample [N]	Exposure	Outcomes	Confounders	Main Results
Childhood Head Growth and Educational Attainment in an Indian Cohort. **Pandey, 2021** India Cohort	To investigate associations of head size at birth and head growth during specific periods in infancy and childhood with educational attainment, as a proxy for cognitive ability.	During 1969-73, 20755 married women living in South Delhi were recruited, resulting in 8030 singleton live newborns, forming the New Delhi Birth Cohort. In this study, women who had their educational attainment evaluated in the phase 5 of the follow-up were included [N=1526]. The "completed cases analysis" included participants without missing data [N=558].	Head circumference (centimeters), conditional growth variables for head circumference [at birth, 6 months, 2, 11 and 26-32 years].	Educational attainment: Participants' educational attainment was recorded in seven categories from no schooling to a professional qualification. Educational attainment was converted from the original seven categories into years of education, from none (0 years) to a professional qualification (17 years) [26-32 years].	Socioeconomic level, sex, gestational age, height, BMI.	Linear Regression (betas): Crude - At birth: 0.30 (CI95%:0.14;0.46); from birth to 6 months: 0.44 (CI95%:0.28,0.60); from 6 months to 2 years: 0.30 (CI95%:0.14;0.46); from 2 to11 years 0.20 (CI95%:0.04;0.38); from 11 anos to adulthood: 0.15 (CI95%:-0.02;0.33); Adjuested: At birth: 0.15 (CI95%:-0.05;0.34); from birth to 6 months: 0.14 (CI95%:-0.03 to 0.31); from 6 months to 2 years: 0.03 (CI95%:-0.13 to 0.19); from 2 to 11 years: 0.03 (CI95%:-0.14 to 0.20); from 11 years to adulthood: 0.08 (IC95%:-0.10 to 0.26)
The impact of nutritional status and longitudinal recovery of motor and cognitive milestones in internationally adopted children. **Park, 2011** United States Cohort	To assess the impact of baseline (immediately post adoption) nutritional status on fifty-eight children as measured by weight-for-age, height-for-age, weight-for-height and head circumference-for-age z scores, as a determinant of cognitive (MDI) and psychomotor development (PDI) scores longitudinally.	Children younger than 42 months of age who presented to the Adoption Health Service at Rainbow Babies and Children's Hospital in Cleveland, Ohio for post-adoptive care within 2 months of immigration, from April 2001 to February 2002 [N=58].	Head circumference z scores according to age, dichotomized into: ≤−2 SD, and >-2 SD (reference group) [post-adoption, 4.7-39.5 months]	Intelligence/Cognition: Bayley Scales of Infant and Toddler Development - 2nd edition [0, 3, 6 and 12 months after the inicial evaluation - post-adoption]	Age	Linear mixed-effect model: Data was not presented. In the text: Head circumference was associated with MDI in the baseline, but not in the follow-ups.

Title, author, year, country, study-design	Objective	Study sample [N]	Exposure	Outcomes	Confounders	Main Results
Primary megalencephaly at birth and low intelligence level. **Petersson, 1999** Sweden Cohort	To evaluate the association between primary megalencephaly at birth and psychosensory conditions and to determine mother-child similarity for primary megalencephaly at birth.	Cohort 1 was obtained by linking the Swedish Medical Birth Registry for the period 1973–1975 with the National Military Service Enrollment Register for the period 1992–1995. Individuals with a chromosomal anomaly, an inborn error of metabolism or birth weight <1,500 g were excluded. The remaining cohort was then divided into five groups based on the medical diagnoses recorded: 1) no malformation or disease diagnosis as specified below; 2) hydrocephalus; 3) malformation of or disease in the CNS or skull; 4) severe eye, ear, or facial malformation; and 5) any major congenital malformation except those listed above, such as dwarfism and limb dysmorphism [N=144273].	Head circumference percentile, dichotomised into > 98th percentile and ≤98th percentile (reference group) [at birth].	Intelligence/Cognition: An intelligence score (ranging from 1 to 9 from a four-part psychological test battery) less than 4 [at military subscription, 18 years].	No adjustment was performed.	Odds Ratio (Confidence intervals were estimated with the test-based method by Miettinen or based on the Poisson distribution when expected values were low): 1.32 (CI95%: 1.11;1.38)
Influence of prenatal and postnatal growth on intellectual functioning in school-aged children. **Pongcharoen, 2012** Thailand Cohort	To assess the relative influence of size at birth, infant growth, and late postnatal growth on intellectual functioning at 9 years of age.	Children who participated in a randomized controlled trial of iron and/or zinc supplementation during infancy in Khon Kaen province in northeast Thailand during the period from 1998 to	Head circumference z-scores; Head growth conditioned on its previous measure [at 4 months, 1 and 9 years]	Intelligence/Cognition: Wechsler Intelligence Scale for Children – 3rd edition, adapted for Thailand population, adjusted for age. Six verbal subtests (information, similarities, arithmetic, vocabulary,	Socioeconomic level, maternal height, maternal schooling, sex, availability of mother at home, school location.	Linear regression(betas): Head circumference at 4 months - Total IQ: 1.8 p< 0.05; Verbal IQ: 1.5 p< 0.05; Performance IQ: 2.0 p< 0.05; from 4 months to 1 year - Total IQ: 0.5 p> 0.05; Verbal IQ: 0.6 p>0.05; Performance IQ:

Title, author, year, country, study-design	Objective	Study sample [N]	Exposure	Outcomes	Confounders	Main Results
		1999. Eligibility criteria were that infants were predominantly breast fed and free from apparent congenital abnormalities. Infants having hemoglobin < 8 g/dL, chronic illnesses, or who were bottle fed were excluded [N=560].		comprehension, and digit span) and 6 performance subtests (picture completion, coding, picture arrangement, block design, object assembly, and symbol search) were administered; Nonverbal Raven's Colored Progressive Matrices [9 years].		0.3 p>0.05; from 1 to 9 years: Total IQ: −0.2 p> 0.05; Verbal IQ: −0.1 p>0.05; Performance IQ: −0.3 p>0.05; Head circumference was not associated with Nonverbal Raven's Colored Progressive Matrices in any age (data not shown).
Growth impairment in very low birthweight children at 12 years: correlation with perinatal and outcome variables. **Powls, 1996** United Kingdom Cross-sectional	To compare the growth of very low birthweight children in early adolescence with that of their normal birthweight peers; to examine the role of factors contributing to growth-parental height, perinatal variables, bone maturity and sexual maturation; to examine the correlation between head growth and cognitive and educational outcome.	Children with very low birth weight treated at the Mersey regional neonatal unit. The cohort was derived from two groups recruited while in primary school: 1) children with a birth weight of < 1251g and birth dates between January 1980 and June 1981, and 2) children with a birth- weight of <1501g, gestation of < 31weeks, and birth dates between January 1982 and November 1983.None of the children had any major neurodevelopmental handicap, and at the time of the original study were in mainstream schools [N=137]. These original controls were also traced and, where possible, used for the present study. VLBW Children with very low birth weight who were now at	Head circumference (centimeters) [11-13.5 years]	Intelligence/Cognition: a shortform of the Wechsler Intelligence Scale for Children-WISC III; Academic performance: The Wechsler Objective Reading Dimensions - WORD; The NFER Basic Maths test [11-13.5 years]	Muito baixo peso ao nascer/status do controle	Linear Regression (betas): Performance IQ: 2.27 (CI95%:0.93;3.61); Verbal IQ: 2.75 (CI95%:1.67;3.83); Total IQ: 2.74 (CI95%:1.56;3.91); Maths: 1.37 (CI95%:0.59;2.15); Reading: 1.23 (CI95%:0.36;2.1); Spelling: 2.03 (CI95%:1.04;3.02); Comprehending: 1.23 (CI95%:0.47;2.0)

Title, author, year, country, study-design	Objective	Study sample [N]	Exposure	Outcomes	Confounders	Main Results
		different schools from their controls had a new control selected from their present school to match for educational experience [N=160, of whom 100 were original controls].				
Head Growth Trajectory and Neurodevelopmental Outcomes in Preterm Neonates. **Raghuram, 2017** Canada Cohort	To evaluate the association between head growth during neonatal and postdischarge periods and neurodevelopmental outcomes of preterm neonates < 29 weeks of gestational age.	Data from the Canadian Neonatal Network and the Canadian Neonatal Follow-Up Network, which included infants of <29 weeks of gestational age admitted to level III NICUs in Canada born between 2009 and 2011, who received neurodevelopmental follow-up assessments at 16 to 36 months corrected age. Infants with major congenital or chromosomal anomalies, planned palliative care before delivery, head circumference less than third percentile for gestational age and sex, unilateral or bilateral severe ventricular enlargement or hydrocephalus requiring surgical drainage were excluded [N=1973].	Head circumference z-scores [at birth, at neonatal intensive care unit discharge, at follow-up assessment (ie, median 21 months)]; The differences between the z scores were calculated for 3 time periods as an estimate of HC growth velocities, and categorized into 5 groups: Group 1: z score difference of −1 to +1 (reference); Group 2: z score difference of <−2; Group 3: z score difference of −1.01 to −2; Group 4: z score difference +1.01 to +2; and Group 5: z score difference >+2.	Intelligence/Cognition: Bayley Scales of Infant and Toddler Development - 3rd Edition < 70 [at 16 and 36 months corrected age]	Gestational age, sex, small for gestational age status, Score for Neonatal Acute Physiology-II (SNAP-II) score, antenatal steroid use, cesarean delivery.	Linear regression (betas): From admission to discharge - Group 2: −1.9 (CI95%:−3.57;−0.24); Group 3: 0.27 (CI95%:−1.32;1.87); Group 4: 1.88 (CI95%:−1.58;5.33); Group 5: −1.47 (CI95%:−5.35;2.41); from discharge to follow-up - Group 2: −8.33 (CI95%:−13.4;−3.23); Group 3: 2.04 (CI95%:−1.71;5.79); Group 4: 0.31 (CI95%:−1.27;1.88); Group 5: −1.35 (CI95%:−2.96;0.25); from admission to follow-up - Group 2: −5.64 (CI95%:−7.83;−3.44); Group 3: −1.84 (CI95%:−3.96;0.29); Group 4: −2.16 (CI95%:−3.95;0.38); Group 5: −1.22 (CI95%:−3.59;1.16); Logistic Regression (odds ratio): Bayley III <70: Group 2 - From admission to discharge: 2.41 (CI95%:1.19;4.87); from discharge to follow-up: 5.35 (CI95%:1.75;16.4);

Title, author, year, country, study-design	Objective	Study sample [N]	Exposure	Outcomes	Confounders	Main Results
						from admission to follow-up: 2.69 (CI95%:1.24;5.86); Group 3 - From admission to discharge: 0.96 (CI95%:0.4;2.31); from discharge to follow-up: 0.55 (CI95%:0.07;4.23); from admission to follow-up: 2.43 (CI95%:1.06;5.53); Group 4 - From admission to discharge: 1.42 (CI95%:0.31;6.54); from discharge to follow-up: 0.36 (CI95%:0.13;0.98); from admission to follow-up: 0.96 (CI95%:0.35;2.64); Group 5 - From admission to discharge: 1.94 (CI95%:0.42;8.91); from discharge to follow-up: 1.23 (CI95%:0.62;2.43); from admission to follow-up: 1.1 (CI95%:0.32;3.8).
Growth Trajectories and Intellectual Abilities in Young Adulthood: The Helsinki Birth Cohort study. **Räikkönen, 2009** Finland Cohort	To evaluate the effects of growth in body size on intellectual abilities, as estimated from measurements ranging from birth to an average age of 20 years, among Finnish men conscripted into the Finnish Defense Forces.	Men who born at Helsinki University Central Hospital (Helsinki, Finland) during the period 1934–1944 and served in the Finnish Defense Forces between 1952 and 1972 [N=2786].	Head circumference z-scores according to gestational age [at birth].	Intelligence/Cognition: The general cognitive ability test scores were obtained from the Finnish Defense Forces Basic Intellectual Ability Test, which included verbal, arithmetic, and visuospatial reasoning and yields a general cognitive ability total score. Measurements of adult intellectual abilities were converted into z scores [at military subscription, mean 20 years].	Gestational age.	Linear regression (betas): Verbal: 0.05 (CI95%:0.00;0.09) p=0.03; Visuospatial: 0.05 (CI95%:0.00;0.09) p=0.04; Arithmetic: 0.07 (CI95%:0.03;0.11) p=0.002;

Title, author, year, country, study-design	Objective	Study sample [N]	Exposure	Outcomes	Confounders	Main Results
Early life origins cognitive decline: findings in elderly men in the Helsinki Birth Cohort Study. **Räikkönen, 2013** Finland Cohort	To examine whether the adverse effects of slow prenatal and postnatal growth on cognitive function persist to old age and predict age related cognitive decline.	Men who born at Helsinki University Central Hospital (Helsinki, Finland) during the period 1934–1944 and served in the Finnish Defense Forces between 1952 and 1972. In 2009, 1,759 men were invited to a re-testing; 53,2% accepted to take part in the study [N=931].	Head circumference z-scores according to gestational age [at birth].	Intelligence/Cognition: The general cognitive ability test scores were obtained from the Finnish Defense Forces Basic Intellectual Ability Test, which included verbal, arithmetic, and visuospatial reasoning and yields a general cognitive ability total score. The results were standardized with mean 100 and SD of 15 [at military subscription, mean 20 years; and at the follow-up, mean 68 years]. The outcome was treated as continuous and categorical variable (top third versus middle and bottom thirds through the cognitive ability evaluations).	Maternal age, maternal height, parity, gestational age, breastfeeding,father's occupational status; highest own achieved level of education in adulthood; diagnoses of stroke and coronary heart disease.	Linear regression (Betas): Cognitive ability at 68 years - crude: 0.97(CI95%:-0.02; 1.97); adjusted: 1.55(CI95%:0.39;2.72); Decrease of cognitive ability through 5 decades - crude: 0.06 (CI95%:-0.01;0.13); adjusted: 0.11(CI95%:0.03;0.19); Logistic regression (Odds ratio): Top third versus middle and bottom thirds of cognitive ability at 20 and 68 years- crude: 1.26 (CI95%:1.06;1.49); adjusted: 1.43 (CI95%:1.14; 1.79).
Postnatal growth and neuropsychological performance in preterm-birth preschoolers. **Raz, 2014** United States Cohort	To examine the relationships between growth indices such as head size or body height (stature) and neuropsychological outcome of preterm birth.	Preterm birth (gestational age <36 weeks) preschoolers, graduates of the William Beaumont Hospital (WBH) Neonatal Intensive Care Unit at Royal Oak, MI, who were born between 1996-2001 and evaluated between 2002-2007. Children with moderate to severe cerebral palsy and intracranial hemorrhage were excluded. Participants were recruited through a follow-up study of preterm children in the	Head circumference z-scores, dichotomized into <15th percentile and ≥ 15th percentile (reference group) [at birth, 3-6 years].	Intelligence/Cognition: the Wechsler Preschool and Primary Scale of Intelligence-Revised (WPPSI-R), including four of the five subtests from the Verbal IQ subscale (Information, Similarities, Vocabulary, and Comprehension) and four of the five subtests from the Performance IQ subscale (Geometric Design, Mazes, Block Design, and Picture Completion) [3-6 years].	Socioeconomic level, gestational age, sex, age at testing (adjusted for prematurity); intrauterine growth z score; multiplicity; total number of complications; preschool body height z score.	Linear Regression ("Effect size" – apparently, similar to a "beta" but for standardized measures): Verbal IQ: Crude – head circumference at birth: 0.349 $p > 0.05$; head circumference in pre-school age: 0.674 $p < 0.001$; Adjusted – head circumference at birth: 0.015 $p > 0.05$; head circumference in pre-school age: 0.510 $p < 0.01$; To investigate the head circumference as a continuous variable, in adjusted analysis, did not reveal significative

Title, author, year, country, study-design	Objective	Study sample [N]	Exposure	Outcomes	Confounders	Main Results
		preschool years and through a related, concurrent study on the preschool outcome of mild intracranial hemorrhage [N=264].				associations with the outcomes.
Physical growth in the neonatal intensive-care unit and neuropsychological performance at preschool age in very preterm-born singletons. **Raz, 2015** United States Cohort	To study the relationship between preschool outcome and the earliest gains observed in postnatal head growth, relative to other indices of physical growth. Specifically, we wished to establish whether relationships exist between gains in head circumference (relative to gains in body-weight or length), from birth to hospital discharge, and intellectual, language, or motor, performance at preschool age.	Children born between 1996 and 2001 with ≤32 weeks of gestational age (extremely prematures), singletons, who were admitted in the Neonatal Intensive Care Unit of William Beaumont Hospital, in Royal Oak, MI. The participants were recruited between 2002 and 2007, in pre-school age. Children with moderate or severe cerebral palsy or intracranial hemorrhage were excluded [N=121].	Difference between head circumference Z-score at birth and hospital discharge (Z-difference). The Z-score at each of the time points was computed as the deviation of attained head circumference from age-specific group means [at birth, at discharge].	Intelligence/Cognition: the Wechsler Preschool and Primary Scale of Intelligence-Revised (WPPSI-R), including four of the five subtests from the Verbal IQ subscale (Information, Similarities, Vocabulary, and Comprehension) and four of the five subtests from the Performance IQ subscale (Geometric Design, Mazes, Block Design, and Picture Completion) [3-6 years].	Socioeconomic level, sex, days hospitalization, total number of complications, weight at birth, intrauterine growth Z-score.	Linear Regression (Betas): Total IQ: -0.19 p=0.04; Verbal IQ: −0.18 p=0.06; Performance IQ: -0.16 p=0.08;
Influence of intrauterine and extrauterine growth on neurodevelopmental outcome of monozygotic twins. **Reolon, 2008** Brazil Cohort	To determine the influence of intrauterine and early postnatal growth on neurocognitive development of monozygotic twins, using intrapair and interpair differences in some anthropometric	Data were derived from the Brazilian Information System on Livebirths (SINASC), a database of all hospital-based live births in the city of Porto Alegre, Brazil, and included twin sets who were born in Porto Alegre between January 2000 and September 2002.	Head circumference (centimeters) [at birth, 12-42 months].	Intelligence/Cognition: Bayley Scales of Infant and Toddler Development, Second Edition. The Mental Development Subscale (MDI) was standardized with mean of 100 and SD of 15 [12-42 months].	Age, sex, fetal growth ratio. Additionally, an intra-pair analysis was conducted, which accounts for shared environmental and genetic factors.	Linear Regression (betas): Head circumference at birth - intra-pair: 1.71 (CI95%: -0.45; 3.87); inter-pair: -1.74 (CI95%: -5.65; 2.16); 12-42 months - intra-pair: 3.20 (CI95%: 1.06; 5.34); inter-pair: 2.28 (CI95%:-0.60; 5.15)

Title, author, year, country, study-design	Objective	Study sample [N]	Exposure	Outcomes	Confounders	Main Results
	measurements collected at birth and at the corrected age of 12 to 42 months.	Exclusion criteria were malformations, neurosensory impairments (cerebral palsy, deafness and blindness), severe psychomotor retardation and twin-to-twin transfusion syndrome [N=72].				
A cumulative risk factor model for early identification of academic difficulties in premature and low birth weight infants **Roberts, 2007** United States Cohort	To examine a model comprised of cumulative risk factors that allows early identification of academic difficulties in premature and low birth weight children.	Indivíduos com peso ao nascer menor de 2.500 g e idade gestacional menor de 37 semanas, recrutados em 8 centros nos Estados Unidos, em 1984. Foram excluídos gêmeos, crianças que apresentaram doença grave ou óbito no período neonatal, paralisia cerebral ou retardo mental (N=494)	Head circumference (centimeters) [at birth].	Academic Performance: Woodcock-Johnson tests of Achievement- Revised - Reading and Math Scores, both normed to have a mean of 100 and a standard deviation of 15 [8 years].	Socioeconomic level, maternal schooling, weight at birth, race.	Linear Regression (betas): Crude: Reading −0.83 (CI95%: −2.39; 0.73), p=0.3; Maths −1.53 (CI95%: −3.03; −0.03), p=0.04 Adjusted: Maths −1.37 (CI95%: −2.70; −0.04), p=0.04
Relation between physical growth and information processing in infants born in India. **Rose, 1994** India Cross-sectional	To examine the relation between two cognitive competencies and several measures of physical growth that are commonly used to index malnutrition in developing countries.	Bebês entre 5 e 10 meses, com peso adequado ou baixo para a idade, recrutados no Hospital Infantil Wadia, Bombaim, Índia, entre outubro de 1984 e dezembro de 1984, desde que não estivessem doentes (a maioria buscou o serviço para receber vacina contra o sarampo (N=166)	Head circumference (centimeters) [5-12 months].	Intelligence/Cognition: Each infant was tested on seven problems: three visual recognition memory (visual-visual) and four cross-modal transfer (tactual-visual). The stimuli for the problems were pairs of three-dimensional geometric forms; members of a pair differed from one another primarily in terms of shape [5-12 months].	No adjustment was performed.	Correlation (non-specified): visual recognition memory: 0.18 ($p<0.05$); cross-modal transfer: 0.19 ($p<0.05$); Total: 0.24 ($p<0.01$)
Cranial size and IQ in Asian Americans from birth to age seven **Rushton, 1997** United States	To evaluate association between Asian brain size and IQ by providing new data on Asian Americans as	O National Collaborative Perinatal Project é um estudo epidemiológico de grande escala patrocinado pelo	Head circumference [at birth, at 7 years].	Intelligence/Cognition: Wechsler Intelligence Scale for Children [7 years].	No adjustment was performed.	Correlation (non-specified): head circumference at birth and IQ at age 7 ($r=0.04$, $p>0.05$); head circumference at 7 years

Title, author, year, country, study-design	Objective	Study sample [N]	Exposure	Outcomes	Confounders	Main Results
Cohort	infants and young children from the Collaborative Perinatal Project.	National Institutes of Health, que coletou dados durante um período de 16 anos de 12 centros médicos nos Estados Unidos, todos em áreas urbanas. Entre 1959 e 1974, 53.043 gestantes foram recrutadas e seus filhos acompanhados desde a gestação até os oito anos de idade. Nos presente estudo foi analisada uma subamostra de americanos asiáticos (N= 100)				and IQ at 7 years (r = 0.24, p <0.05).
Infant growth after preterm birth and neurocognitive abilities in young adulthood. **Sammallahti, 2014** Finland Cohort	To examine whether faster growth from birth to term (40 postmenstrual weeks) and during the first year thereafter was associated with better neurocognitive abilities in adults born preterm with very low birth weight.	Data from cohort of the Helsinki Study of Very Low Birth Weight Adults, which included children who were born with less than 1500g, between January 1978 and December 1985, and discharged alive from the Neonatal Intensive Care Unit of Children's Hospital at Helsinki University Central Hospital in Finland. Participants who presented neurosensory impairments were excluded [N=103].	Head circumference z-scores according to age and sex; Head growht adjusted for its previous measure [at birth, 40 weeks, 12 months corrected age].	Intelligence/Cognition: Wechsler Adult Intelligence Scale III, including subtests of vocabulary, digit span, similarities, and block design. The results were standardized by age and sex [22-30 years].	Sex, age, time period between closest true measurement point and age at neurocognitive testing, highest education of a parent, and neonatal complications and illnesses (septicemia, bronchopulmonary dysplasia, indomethacin treatment, surgery because of patent ductus arteriosus, blood exchange transfusion because of hyperbilirubinemia, intraventricular hemorrhage, and duration of ventilator treatment).	Linear Regression (Betas; CI presented in a graphic): Head circumference from birth to 40 weeks – Total IQ: 0.42 (CI did not include the null value); Verbal IQ: 0.33 (CI did not include the null value); Performance IQ: 0.37 (CI did not include the null value); From term to 12 months: Total IQ: 0.01 (CI did not include the null value); Verbal IQ: -0.17 (CI did not include the null value); Performance IQ: 0.19 (CI did not include the null value). Sectional measures of the head circumference (in the supplementary materials) – Total IQ: head circumference at birth: 0.18 (CI95%:0.03;0.34); 40 weeks: 0.18 (CI

Title, author, year, country, study-design	Objective	Study sample [N]	Exposure	Outcomes	Confounders	Main Results
						95%:0.10;0.26); 12 months: 0.44 (CI95%:0.14;0.73).
Nutritional deficiencies in disadvantaged preschool children. Their relationship to mental development. **Sandstead, 1971** United States Lack of information provided to define the study design.	To evaluate the nutritional status of preschool children from the economically depressed area of Nashville and to correlate it with Stanford-Binet test scores.	One hundred preschool children attending two day nurseries in the economically depressed area of Nashville, Tenn, were evaluated, without prior knowledge of their scores on the Stanford-Binet test of "intelligence," or their individual degree of participation in the language development program of the Bill Wilkerson Hearing and Speech Center. Stanford-Binet tests were administered to 76 of the children by investigators from the Bill Wilkerson Hearing and Speech Center both prior and subsequent to instruction in language [N=76].	Head circumference according to age ["school age" - the article did not present the precisely age of the participants].	Intelligence/Cognition: Stanford-Binet tests were administered both prior and subsequent to instruction in language ["school age" - the article did not present the precisely age of the participants].	No adjustment was performed.	Correlation (non-specified): Initial IQ (n=68): 0.0643 p>0.05; Final IQ (n=66): -0.0210 p>0.05
Early childhood growth and cognitive outcomes: Findings from the MAL-ED study. **Scharf, 2018** Bangladesh, India, Nepal, Peru e Africa do Sul Cohort	To evaluate measures of growth as predictors of cognitive development at 24 months using three growth parameters (length, weight, and head circumference) and four constructs for evaluating growth (birthweight, individual measures at specific time	Data from The Etiology, Risk Factors, and Interactions of Enteric Infections and Malnutrition and the Consequences for Child Health (MAL-ED) study, which included approximately 200 children from each field site in areas of malnutrition and enteric disease in eight low- and middle-income countries: Bangladesh	Head circumference z-scores (HCZ) [monthly from recruitment (mean 7 days) to 24 months]The authors assessed four growth constructs: 1. Initial size: Cross-sectional HCZ at enrolment within 17 days of birth (proxy for	Intelligence /Cognition: Bayley Scales of Infant and Toddler Development (BSID III) [24 months].	Research site, enrolment weight.	Linear Regression (beta): Monthly anthropometry measures and cognitive development: for an average increase of 1 HCZ, Bayley cognitive score (range of 0 to 15) increased 0.37 points. Summative head circumference from 12 to 18 and 18 to 24 months was significantly related to cognitive skills; Rate of growth and child development: Growth rate in length 6 to 12 months,

Title, author, year, country, study-design	Objective	Study sample [N]	Exposure	Outcomes	Confounders	Main Results
	points, summative growth, and rate of growth). We sought not to examine all proximal determinants of development, but instead to determine which components of growth best correlate, to inform future studies considering anthropometric outcomes when direct cognitive assessment is not possible.	(Dhaka, urban), Brazil (Fortaleza, urban), India (Vellore, urban), Nepal (Bhaktapur, urban), Peru (Loreto, rural), Pakistan (Naushahro Feroze, rural), South Africa (Venda, rural), and Tanzania (Haydom, rural). Children born <1,500 g, who had serious illness or extended hospital stays, multiple gestations, and whose mothers were <16 years, were excluded. Upon analysis of quality, the Bayley assessments from Tanzania site and the head circumference data from Pakistan and Brazil sites were not found to have sufficient quality and thus were excluded [N=1210].	birthweight); 2. Attained size: HCZ at monthly cross sections from 0 to 2 years; 3. Summative growth: Area between child's growth curve and the WHO growth curve; areas calculated from birth to 24 months and in 6-month intervals; 4. Growth rate: $\Delta HCZ/\Delta t$ where Δt was time from birth to 24 months in 6-month intervals.			and head circumference 6 to 12 and 12 to 18 months were positively related to cognitive scores. Rapid rate of change for weight, length, and especially head circumference at 18 to 24 months was related to lower scores (graphical presentation).
Microcephaly in a normal school population.<br>**Sells, 1977**<br>United States<br>Cross-sectional	(1) To examine the prevalence of microcephaly, defined as a head circumference 2 SD below the mean in a normal school population; (2) to determine IQ and academic achievement of individuals with a head circumference 2 SD below the mean: and (3) to evaluate the association between	Students aged 5 to 18 years, attending regular classes in 4 schools in Seatle in 1972, representative of the entire district's student population [N=1006]. From de total, 19 students had head circumference less or equal 2 SD below the means for age were selected.	Head circumfence z-scores, dichotomized into < - 2 SD or ≥ -2 SD (control group) [5-18 years].	Intelligence/Cognition: The Lorge-Thorndike was the primary group intelligence test used (eight students) and the Wechsler Intelligence Scale for Children (WISC) (three students). Seven students did not have IQ data available for analysis. Academic Performance: The Comprehensive Test of Basic Skills (CTBS), expressed in national percentiles [5-18 years].	No adjustment was performed.	Mean (T Test): Mean IQ: 99.5 vs 105.0 (controls) p>0.05; Academic performance: 49 vs 70 (controls) p=0.001

Title, author, year, country, study-design	Objective	Study sample [N]	Exposure	Outcomes	Confounders	Main Results
	height and IQ and academic achievement in these individuals.					
Head circumference, total cerebral volume and neurodevelopment in preterm neonates. **Selvanathan, 2021** Canada Cohort	To determine whether early neonatal head growth is associated with preschool-age neurodevelopment in very preterm neonates and whether suboptimal head growth is associated with poorer neurodevelopment.	Very preterm neonates (24–32 weeks' gestational age) were recruited from April 2006 to September 2013 at British Columbia Women's Hospital, Vancouver, Canada. Neonates with congenital malformation or syndrome, hydrocephalus requiring shunt insertion, antenatal infection or sonographic evidence of large parenchymal haemorrhagic infarction (>2 cm) were excluded [N=168].	Head circumference percentiles, dichotomized into < 10th percentile and ≥ 10th percentile (reference group) [at birth, at ICU discharge].	Intelligence/Cognition: The Bayley Scales of Infant and Toddler Development Third Edition (Bayley-III) [18 and 36 months of corrected age]; The Wechsler Preschool and Primary Scale of Intelligence, Third Edition (both normalized, with mean of 100 and SD of 15) [4.5 years].	Gestational age (basic model) + retinopathy of prematurity, white matter injury (final model).	Linear regression (betas): Total IQ: Head circumference at birth - basic model: -8.9 (CI95%:−15.0;−2.9); final model: -7.2 (CI95%:−13.0;−1.4); head circumference at birth/at discharge - basic model: regular HC at birth/small head circumference at discharge: -1.8 (CI95%:−9.3;5.7); small/regular: -10.4 (CI95%:−19.7;−1.1); small/small: -8.8 (CI95%:−16.4;−1.3); final model: regular/small: -4.0 (CI95%:−11.0;3.0); small/regular: -1.3 (CI95%:−11.1;8.5); small/small: -11.3 (CI95%:−18.4;−4.3); Bayley Scale at 18 months: Head circumference at birth: −5.0, p=0.03; Bayley at 36 months: −8.6, p=0.001 (final model); head circumference at birth/at discharge (small/small): Bayley at 18 meses: −7.4, p=0.008; Bayley at 36 months: −9.2, p=0.002 (final model).

Title, author, year, country, study-design	Objective	Study sample [N]	Exposure	Outcomes	Confounders	Main Results
The relative effect of size at birth, postnatal growth and social factors on cognitive function in late childhood. **Silva, 2006** United Kingdom Cohort	To investigate if fetal, head, and somatic postnatal growth are independent predictors of cognition and estimate their relative importance compared to the effect size of social factors on cognitive function at 10 years.	Data from the British Birth Cohort Study, which included individuals born during 5–11 April 1970 in the United Kingdom. Nonsingleton births and stillbirths, and children with incomplete data on all four measures of cognitive function were excluded [N=11244].	Head circumference (centimeters) [at 5 and 10 years].	Intelligence/Cognition: a latent construct measured by four indicators: The British Ability Scale is a self-completion test of cognitive attainment, composed of 120 items, comprising two verbal (word definitions and word similarities) and two-nonverbal sub-scales (recall of digits and matrices). The Shortened Edinburgh Reading Test is a self-completion test of word recognition which contains 67 items; The Friendly Maths Test consists of a total of 72 multiple choice questions and The Pictorial Language Comprehension Test is composed of 100 items. The total score represents the count of all items answered correctly [10 years].	Socioeconomic level, maternal age, maternal height, parity, paternal height, participant age and sex, breastfeeding, birth weight, Height at 5 and 10 years, Weight at 10, Age child started school, Number of days read to child in the past week, Maternal smoking, Marital status, Ethnicity.	Linear Regression (Betas): non-standardized coefficients: head circumference at 5 years: 0.039; at 10 years: 0.034; standardized coefficients: head circumference at 5 years: 0.073; at 10 years: 0.065; Structural equation modelling (Betas): Direct effect: non-standardized coefficients: head circumference at 5 years: 0.068; at 10 years: 0.058; Indirect effect: Coeficientes padronizados: head circumference at 5 years: 0.028; at 10 years: 0.000; p<0.001
Genetic and environmental contributions to the association between anthropometric measures and iq: a study of Minnesota twins at age 11 and 17. **Silventoinen, 2012** United States Cross-sectional	To investigate the associations of different anthropometric measures with IQ in twins during late childhood and late adolescence. The twin-study design provided an opportunity to analyze how genetic and environmental factors contribute to these associations.	Data from the Minnesota Twin Family Study, which included monozygous (MZ) and same-sex dizygous (DZ) twin pairs born in Minnesota from 1972 to 1984, identified from Minnesota state birth records. Twins and their families were invited to a day-long assessment in a laboratory when the twins were approximately 11 or 17 years of age; 17%	Head circumference according to sex and age [at 11 or 17 years].	Intelligence/Cognition: An abbreviated version of the Wechsler Adult Intelligence Scale-Revised (WAIS-R) [11 years]. or the Wechsler Intelligence Scale for Children-Revised (WISC-R) [17 years]. The short forms consisted of two Verbal subtests (Information and Vocabulary) and two Performance subtests (Block Design and Picture Arrangement). Results	Adjust was not performed for specific confounders.	Standardized regression estimates using the clustered samples option (Phenotypic correlations) 11 years - Boys: 0.21 (CI95%:0.09;0.33), Girls: 0.13 (CI95%:0.04;0.22); 17 years - Boys:0.17 (CI95%:0.06;0.27), Girls: 0.14 (CI95%:0.05;0.23) Decomposition of statistically significant correlations between anthropometric traits and full-scale IQ into additive genetic and specific

Title, author, year, country, study-design	Objective	Study sample [N]	Exposure	Outcomes	Confounders	Main Results
		refused to participate [N=2764].		were adjusted for age and sex.		environmental factors (using a bivariate Cholesky decomposition): Additive genetic correlation: r 0.32 (CI95%:0.14;0.50); Specific environmental correlation: 0.21 (CI95%:0.09;0.33)
Abnormal head circumference in learning-disabled children **Smith, 1981** United States Case-control	To test the hypothesis that there is a significant prevalence of abnormal head circumference in children with learning disabilities, documented by recognized criteria.	Cases: All learning-disabled children seen in three Salt Lake City area school district child development clinics between 1st September 1977 and 15th April 1979, who presented the following criteria: absence of medical problems precluding normal learning, IQ ≥ 85, learning quotient <90 in one or more areas, and deficits > 2SD below mean in one or more areas). Controls: group of children with average or better classroom performance was drawn from the same school districts, IQ ≥ 85, learning quotient ≥ 90, from the same school districts where the cases were from, paired by sex and school level [Cases=73; controls 71, total =144].	Head circumference z-score according to age, categorized into ≥ +2 SD, from +2 to -2 SD (reference group) and < -2SD [at 6-13 years].	Academic performance: Learning quotient - a ratio of child's measured academic achievement versus expected achievement in relation to mental age, chronological age and grade level (Illinois Test of Psycholinguistic Abilities, Bender Gestalt and Frostig Test of Psycholinguistic Perception) < - 2SD [at 6-13 years].	Just bivariate analysis was performed, but the results were stritified by sex, age and school level.	Prevalence: Boys: Head circumference ≥ +2 SD: cases 12.5% and controls 3.6%; from -2 to +2 SD: cases 82.2% and controls 96.4%; <-2 SD: cases 5.3% and controls 0% (p<0.05) Girls: Head circumference ≥ +2 SD: cases 5.9% and controls 0%; from -2 to +2 SD: cases 70.6% and controls 100%; <-2 SD: cases 23.5% and controls 0% (p<0.05)
Impact of neonatal growth on IQ and behavior at early school age. **Smithers, 2013**	To examine associations of neonatal weight gain and head circumference gain	Data from the Promotion of Breastfeeding Intervention Trial (PROBIT), which	Neonatal gain in head circumference (HCG): the gain in head	Intelligence/Cognition: The Wechsler Abbreviated Scales of Intelligence (WASI), which included the vocabulary and	Clustering by polyclinic, age at measurement of head circumference, residential strata,	Linear Regression (betas): the highet vs the lowest quartile of gain in head circumference: total IQ:1.5 (CI95%:0.9;2.2),

Title, author, year, country, study-design	Objective	Study sample [N]	Exposure	Outcomes	Confounders	Main Results
Belarus<br>Cohort	with IQ scores and behavior at early school age.	included singleton infants born at ≥ 37 weeks' gestation and weighing ≥ 2500 g during 1996 to 1997 in Belarus. The current study was conducted on the children who had anthropometric measurements at 1 month, all relevant co-variables, and follow-up assessments of IQ or behavior at 6.5 years of age [N=13840].	circumference from birth to 1 month, divided by birth head circumference, and multiplied by 100%, categorized into quartiles. The lowest quartile, which reflects the lowest relative gain in head circumference during the neonatal period, was designated the reference group [at birth, at 1 month].	similarities subtests to measure verbal IQ, and the block design and matrix reasoning subtests were used to measure performance IQ. Raw subtest scores were converted to age- and gender-standardized scores [6.5 years].	maternal and paternal occupation and schooling, 5-min Apgar scores, complications during delivery and the postpartum period, cesarean delivery, older siblings, treatment group.	performance IQ: 1.5 (CI95%:0.8;2.2), verbal IQ: 1.3 (CI95%:0.6;1.9).
Head circumference in ELBW babies is associated with learning difficulties and cognition but not ADHD in the school-aged child.<br>**Stathis, 1999**<br>Australia<br>Cohort	To examine whether a small head circumference and low head-circumference growth velocity during the first year of life predict consequences at school age in learning, cognition, and concentration.	Infants with extremely low birth weight (birthweight 500 to 999g) who were born between 1977 and 1986 and received neonatal care at the Mater Misercordiae Mother Hospital, Brisbane, Australia [N=87].	Head circumference percentile, dichotomized into ≤ 10th percentile and > 10th percentile (reference group) [at birth, 4, 8, 12 and 24 months of corrected age].	Intelligence/Cognition: the McCarthy Scale's General Cognitive Index (GCI); Academic performance - Learning difficulty: a Children delayed by >1 year in reading, mathematics, writing, or spelling, based on a detailed ANSER questionnaire completed by the children's teachers [6 years].	Maternal age, maternal schooling, weight at birth, gestational age, sex, multiple births, the number of days ventilated, or a history of intraventricular hemorrhage.	Crude analysis: Mean difference: GCI: head circumference ≤10th percentile vs >10th percentile: at birth: –0.9 (CI95%: –10.0;8.2); 4 months: –10.1 (CI95%:–18.7; –1.5); 8 months: –11.0 (CI95%:–20.0;–2.0); 12 months: –9.4 (CI95%:–18.4; –0.4); 24 months: –6.6(CI95%:–16.4; 3.2) Chi-squared - percentual (%) of children with learning difficulty: At birth - head circumference < 3rd percentile: 64 (CI95%:31;89); from 3rd to 10th percentile: 50 (CI95%: 23;77); > 10th percentile: 45 (CI95%:32;59), p=0.54; 4 months - head

Title, author, year, country, study-design	Objective	Study sample [N]	Exposure	Outcomes	Confounders	Main Results
						circumference < 3rd percentile: 63 (CI95%: 35;85); from 3rd to 10th percentile: 54 (CI95%:25;81); > 10th percentile: 43 (CI95%:29; 59) , p=0.32; 8 months - head circumference < 3rd percentile: 61 (CI95%:39;80); from 3rd to 10th percentile: 77 (CI95%:46;95); > 10th percentile: 30 (CI95%:17; 47), p= 0.004<br>Adjusted analysis: Logistic regression (Odds ratio): Head circumference at 8 months and learning difficulty: 4.7 (CI95% 1.9;13.6) p< 0.05; Linear Regression (Mean difference): Head circumference at 8 months and cognition: 8.3, p=0.04
Growth and development of term children born with low birth weight: effects of genetic and environmental factors.<br>**Strauss, 1998**<br>United States<br>Cohort	To evaluate the role of intrauterine growth retardation (IUGR) on childhood growth and development, controlling for environmental and genetic factors.	The National Collaborative Perinatal Project prospectively monitored 59,393 women enrolled during their pregnancy and their 55,760 children from 1959 to 1976. A total of 8411 women were enrolled for at least two different pregnancies. Infants were excluded if they had a gestational age <37 weeks, multiple gestation, neural tube defect, chromosomal anomaly, or other severe congenital	Head circumference z-scores, dichotomized into < -2 SD and ≥ -2 SD [at birth].	Intelligence/Cognition: Wechsler Intelligence Scale for Children [7 years].	No adjustment was performed, but bivariate analysis comparing the groups of siblings.	T test (Mean difference): Children with <2500g and head circumference 3 or more cm lower vs their siblings: QI: 86.0 ± 15.7 vs 92.0 ± 14.4, p<0.02 ; Children with <2500g and head circumference less than 3 cm lower vs their siblings: QI: 92.4 ± 11.8 vs 93.1 ± 13.3, p=0.6 Children with <2500g and head circumference < -2 SD vs children with <2500g and head circumference ≥ -2 SD: IQ: 86.0 ± 13.2 vs 94.2 ± 12.8, p<0.001

Title, author, year, country, study-design	Objective	Study sample [N]	Exposure	Outcomes	Confounders	Main Results
		diseases. To control for genetic and environmental factors, the same growth and development assessments were compared in a subpopulation of the population cohort, which consisted of similar-sex sibling pairs in which one sibling was born with ≤ 2500 g and one was born > 2500 g (sibling cohort) [N=440, infants with ≤ 2500 g at birth=32].				
Anthropometric assessment and school achievement in school-age children from high school in Valparaiso, Chile. **Toro Diaz, 1998** Chile Cross-sectional	To determine the interrelationship between nutritional status and scholastic achievement.	Children attending grades 1 to 4 in schools in Valparaíso, Chile, selected by the Ministry of Education to participate in a monitoring program [N=165].	Head circumference percentile, dichotomized into < 50th percentile and ≥ 50th percentile (reference group)[14-20 years].	Academic performance: Spanish and Maths tests applied for each school year, expressed in percentual of corrected aswers by exam and total school performance [14-20 years].	No adjustment was performed.	Correlation (Pearson): Grade I: 0.034 (p>0.05); Grade II: -0.000 (p>0.05); Grade III: -0.136 (p>0.05); Grade IV: 0.443 (p< 0.05);Relative risk for academic performance < 50th percentile - participants with head circumference <50th percentile vs the reference group 1.97, p<0.05
Association of Birthweight and Head Circumference at Birth to Cognitive Performance in 9-to 10-Year-Old Children in South India: Prospective Birth Cohort Study **Veena, 2010** India Cohort	To test the hypothesis that lower birthweight and smaller head circumference at birth are associated with poorer scores in tests of cognitive function, independent of socioeconomic factors.	Data from the Mysore Parthenon study which included women who booked consecutively into the antenatal clinic of the Holdsworth Memorial Hospital, Mysore, between June 1997 and August 1998 and had a singleton pregnancy, <32 weeks of gestation ager determined by last menstrual period date or	Head circumference z-scores [at birth].	Intelligence cognition: three core tests from the Kaufman Assessment Battery for children-second edition and additional tests that covered the domains of short-term memory, long-term memory and retrieval ability, visuospatial ability, and language production. All tests were standardized [9-10 years].	Sex, gestational age and current age (Model 1) + socioeconomic level (Standard living index), maternal and paternal schooling (Model 2) + parity, maternal age, maternal height, maternal BMI, urban or rural residence and time of the day when cognitive tests were	Linear Regression (betas): Atlantis: Model 1: 0.2 (CI95%: 0.07; 0.26) p≤0.01, Model 2: 0.1 (CI95%:0.03; 0.21) p≤0.01, Model 3: 0.1 (CI95%: 0.04-0.23) p≤0.01, Model 4: 0.08 (CI95%:-0.02;0.19) p>0.05; Word order: Model 1: 0.1 (CI95%:0.01; 0.19) p≤0.05, Model 2: 0.05 (CI95%: -0.04;0.15) p>0.05, Model 3:

Title, author, year, country, study-design	Objective	Study sample [N]	Exposure	Outcomes	Confounders	Main Results
		a first trimester ultrasound scan and no prior history of diabetes. Children with major medical problem were excluded.			administered (Model 3) + child's current head circumference (Model 4).	0.07(CI95%:-0.02; 0.16) p>0.05, Model 4: 0.004 (CI95%:-0.10;0.11) p>0.05; Pattern reasoning: Model 1: 0.06 (CI95%: -0.04;0.15) p>0.05, Model 2: -0.01(CI95%:-0.09; 0.08) p>0.05, Model 3: 0.001 (IC 95%: -0.09; 0.09) p >0.05, Model 4: -0.06(CI95%:-0.16;0.04)p>0.05
The growth of very-low-birth-weight infants at 5 years old in Taiwan. **Wang, 2014** Taiwan Cohort	To compare the growth and effect of growth on cognitive performance at 5 years of age of a group of very-low-birth-weight infants and a group of healthy full-term infants.	Very low birth weight infants who were discharged from 3 hospitals located throughout the island of Taiwan (National Taiwan University Hospital, Mackay Memorial Hospital, and Women and Children's Campus, Taipei City Hospital) between January 1995 and June 1996 [N=224].	Head circumference z-scores, dicothomized intohead circumference <-2 SD (apparently in any measurement, this information is not clear in the text) and reference group [at 6, 12, 24 months, and 5 years].	Intelligence/Cognition: the Wechsler Preschool and Primary Scale of Intelligence [5 years].	No adjustment was performed.	Mean (T Test): total IQ - head circumference <-2 SD: 78.9; reference group: 91.6 p<0.05; verbal IQ - head circumference <-2 SD: 78.0; reference group: 88.6 p<0.05; performance IQ - head circumference <-2 SD: 81.6; reference group: 96.5 p<0.05;
Intelligence, reading achievement, physical size and social class. A study of St. Louis Caucasian boys aged 8-0 to 9-6 years, attending regular schools. **Weinberg, 1974** United States Cross-sectional	(1) To report the relationship of HC, height, weight, and skeletal age to measures of intellectual functioning (I.Q. and reading proficiency); (2) to note which of these variables correlates closely with social class; and (3) to investigate the effect of social class differences on the	Caucasian boys between the ages of eight years zero months and nine years six months, attending three St. Louis City and four St. Louis County regular schools [N=334].	Head circumference (centimeters) [8 - 9.5 years].	Intelligence cognition: Wechsler Intelligence Scale for Children, including the Vocabulary, Similarities, and Block Design subtests and (2) the Peabody Picture Vocabulary Test (PPVT). Academic Performance: Reading achievement was determined by the reading subtest of the Wide Range Achievement Test [8-9.5 years].	Socioeconomic level (the Hollingshead-Redlich Two Factor Index of Social Class Position, including the number of years of education and occupational achievement by the natural father)	Correlation (Pearson): Crude: IQ r=0.351 p<0.001; PPVT r=0.409 p<0.001; WRAT r= 0.247 p<0.001; Adjusted: IQ r=0.213 p<0.001; PPVT r=0.304 p<0.001; WRAT r= 0.087 p>0.05 Figure 1 shows the relationship between PC and IQ. In the text: "As can be clearly seen, mean IQ increases approximately 6 points with each increment of 1 cm in HC up to 54 cm,

Title, author, year, country, study-design	Objective	Study sample [N]	Exposure	Outcomes	Confounders	Main Results
	relationship of given variables in this heterogeneous group of nonreferred schoolboys attending regular schools.					with a leveling off at this point."
Head growth and neurocognitive outcomes. **Wright, 2015** United Kingdom Cohort	To describe the incidence of head centile shifting and the relationship between extremes of head size and later neurodevelopmental problems in the Avon Longitudinal Study of Parents and Children.	Data from the Avon Longitudinal Study of Parents and Children (ALSPAC) which recruited pregnant women who were resident in the former Avon Health Authority in southwest England in 1991–1992, resulting in a cohort of 15247 pregnancies and 14701 children who were alive at 12 months. This analysis included children with 2-3 head circumference measurements through the period [N=9279].	Head circumference percentiles; A summary infancy head z score was calculated for each child by using the average of the internally standardized SD scores at 8 weeks and 9 months plus the 18 or 24 month value where available. The variable was then categorized into head circumference <2nd percentile, from 2nd to 98th percentile (reference group) >98th percentile [8 weeks, 9 and 24 months].	Intelligence/Cognition: the Wechsler Intelligence Scale for Children, 3rd edition; A low IQ was defined as total IQ < 70 [11 years]. Academic Performance: Educational records linkage to the Pupil Level Annual Schools Censusdata set for 2003/2004 were used to identify all children recorded as receiving extra classroom support with a Statement of Special Educational Needs. A retrospective notes review for all of these children was then undertaken by a team of researchers led by an experienced developmental pediatrician to identify learning disability, classified as mild or moderate/severe [11 years].	No adjustment was performed.	Percentual (Chi-quared test): IQ <70: Reference group: 3.4; <2nd percentile: 10.9 p=0.021; >98th percentile: 11.1 p=0.006; Statement of Special Educational Needs: Reference group: 2.8; <2nd percentile: 6.6 p<0.001; >98th percentile: 1.8 p=0.24; learning disability - mild: reference group: 0.6; <2nd percentile: 4.9 p=0.005; >98th percentile: 3.3 p=0.33; moderate/severe: reference group: 0.2; <2nd percentile: 5.3 p=0.005; >98th percentile: 0 p=0.33;
Robust determinants of neurocognitive development in children: evidence from the Pune Maternal Nutrition Study.	To investigate the association of in utero, birth, and childhood conditions with offspring neurocognitive	Women in reproductive age group were recruited from the farming communities of 6 villages near Pune. Approximately 800 pregnancies were	Head circumference (centimeters) [at birth and 12 years].	Intelligence/Cognition: Raven’s Coloured Progressive Matrices that measures global neurocognitive ability, Block Design tests that measures visuo-spatial	Head circumference at birth: Gender, Length, Birth weight; Head circumference at 12 years: Height, Weight, head circumference,	Head circumference at birth - colour progressive matrices: −0.02 (CI95%:−0.14; 0.1); Picture completion: 0.14 (CI95%:0.02, 0.27); Digit span forward: 0.11

Title, author, year, country, study-design	Objective	Study sample [N]	Exposure	Outcomes	Confounders	Main Results
**Yajnik, 2022** India Cohort	development at age 12 years.	investigated in detail and 762 live births occurred from June 1994 to April 1996 [N=686].		processing, Picture Completion test that measures visual attention, Digit Span tests that measure attention and working memory, Colour Trail Making tests that measure focused and divided attention, and Auditory Verbal Learning Test that measures verbal learning and memory. The results were standardized [12 years].	haemoglobin, fasting glucose and insulin, cholesterol, triglycerides, vitamin B12, folate, homocysteine, and ferritin.	(CI95%:−0.02, 0.24); digit span backward: 0.08 (CI95%:−0.05, 0.2); colour trail making test A: −0.11 (CI95%:−0.24, 0.03); colour trail making test B: −0.05 (CI95%:−0.18, 0.08), auditory learning verbal test: 0.04 (CI95%:−0.09, 0.17); block design (total correct score): 0.09 (CI95%:−0.04, 0.22); Head circumference at 12 years - colour progressive matrices: 0.07 (CI95%:−0.01, 0.14); Picture completion: 0.02 (CI95%:−0.05, 0.1); Digit span forward −0.01 (CI95%:−0.09, 0.06); digit span backward: 0.004 (CI95%:−0.07, 0.08); colour trail making test A: −0.001 (CI95%:−0.08, 0.08); colour trail making test B 0.03 (CI95%:−0.04, 0.11); auditory learning verbal test: −0.01 (CI95%:−0.08, 0.07); block design (total correct score): −0.04 (CI95%:−0.12, 0.03).
Effect of first-month head-size growth trajectory on cognitive outcomes in preterm infants. **Yu, 2021** Taiwan Cohort	To examine whether the patterns of head-size growth trajectory in the first month after birth are associated with different susceptibility to cognitive impairment	Very preterm infants with gestational age ≤ 30 weeks, who were admitted to the NICU at National Cheng Kung University Hospital in southern Taiwan from January 2001 to December 2016 and followed-up up to the corrected age of 24	Head circumference (HC): weekly percentage of changes from week 1 to week 4 compared to the baseline at-birth head circumference, for which were	Intelligence/Cognition: Mental Developmental Index (MDI) from the Bayley Scales of Infant Development second edition (BSID-II - 47.4% of the sample); the cognitive and language composite (CLC) scores from the BSID-III (52.6% of the sample); The	Hemodynamically significant patent ductus arteriosus requiring intervention, respiratory distress syndrome requiring surfactant therapy, lower gestational age.	Logistic Regression (Odds ratio): Cognitive impairment: delayed catch-up vs. slow catch-up: 4.0 (CI95%: 1.8;8.5) p=0.001; fast catch-up vs. slow catch-up: 1.6 (CI95%:0.5;5.0) p=0.40; Borderline delay: delayed catch-up vs. slow catch-up: 1.5 (CI95% 0.9;2.5)

Title, author, year, country, study-design	Objective	Study sample [N]	Exposure	Outcomes	Confounders	Main Results
	outcomes at age 24 months.	months. Children with severe brain injuries (grade III-IV intraventricular hemorrhage, post-hemorrhagic hydrocephalus, and cystic periventricular leukomalacia), severe hearing or visual impairment, brain malformations, chromosomal anomalies or who were born small-for-gestational age were excluded from analysis [N=403].	defined three distinct trajectories: (1) the delayed catch-up which after the initial 4% decline in head circumference in the first two weeks after birth, barely regained their baseline HC at 4 weeks after birth; (2) the slow catch-up group which showed a 1% decline in HC in the first week, regained the baseline HC by second week, and then continued to increase in head size well above the baseline HC by 4 weeks; and (3) the fast catch-up group which did not show any decline in HC after birth and maintained their HC growth well above the baseline HC in the first 4 weeks after birth [at birth and weekly during the first month of life].	cognitive outcome was classified as normal with the MDI > 85 or CLC >91, borderline delay with MDI from 70 to 84 or CLC from 78 to 90, and impaired with MDI 69 or CLC [24 months of corrected age].		p=0.09; fast catch-up vs. slow catch-up: 0.8 (CI95%:0.4;1.5) p=0.52

Title, author, year, country, study-design	Objective	Study sample [N]	Exposure	Outcomes	Confounders	Main Results
Head circumference trajectories during the first two years of life and cognitive development, emotional, and behavior problems in adolescence: a cohort study. **Zhu, 2022** China Cohort	To identify head circumference growth–sensitive periods associated with cognitive development and emotional and behavioral problems in early adolescence (aged 10–14 years), and to estimate head circumference trajectories during the first 2 years of life, which may help to clarify the link between early-life head circumference growth and child developmental outcomes.	Data from a prospective birth cohort of children born to mothers who participated in a double-blind, cluster-randomized controlled trial in rural western China, which included pregnant women from every village in two counties between 1 August 2002 and 28 February 2006. A total of 1400 singleton births from the parent trial in 2004 were enrolled for long-term follow-up [N=403].		Intelligence/Cognition: the Wechsler Intelligence Scale for Children, Fourth Edition, age-standardized [10-14 years].	Socioeconomic level, maternal age, maternal schooling, parity, paternal age, paternal schooling, sex, parental job, randomized regimen by durations, type of delivery, small-for-gestational age.	General estimating equation linear models (beta): Data shown in figures. In text: for adolescent FSIQ, we observed that the point estimate of conditional head circumference growth increased up to 18 months of age and then decreased during the post- 18 months. Besides, the conditional HC growth between 3 and 6 months and between 12 and 18 months of age reached statistical significance, with an adjusted mean difference of 0.62 (95% CI 0.02, 1.22) and 0.66 (95% CI 0.04, 1.27) adolescent FSIQ points for each standardized residual increase. In addition, HC conditional growth between the periods of 0–6, 0–12, 0–18, and 0–24 months of age was statistically significantly associated with adolescent cognitive scores, but not for 18–24 months of age.
